# Genomic evidence for hybridization and introgression between blue peafowl and endangered green peafowl and molecular foundation of leucistic plumage of blue peafowl

**DOI:** 10.1093/gigascience/giae124

**Published:** 2025-02-19

**Authors:** Gang Wang, Xinye Zhang, Xiurong Zhao, Xufang Ren, Anqi Chen, Wenting Dai, Li Zhang, Yan Lu, Zhihua Jiang, Huie Wang, Yong Liu, Xiaoyu Zhao, Junhui Wen, Xue Cheng, Yalan Zhang, Zhonghua Ning, Liping Ban, Lujiang Qu

**Affiliations:** College of Animal Science and Technology, China Agricultural University, Beijing 100091, China; College of Animal Science and Technology, China Agricultural University, Beijing 100091, China; College of Animal Science and Technology, China Agricultural University, Beijing 100091, China; College of Animal Science and Technology, China Agricultural University, Beijing 100091, China; College of Animal Science and Technology, China Agricultural University, Beijing 100091, China; College of Grassland Science and Technology, China Agricultural University, Beijing 100091, China; Beijing Key Laboratory of Captive Wildlife Technologies, Beijing Zoo, Beijing 100091, China; Institute of Animal Husbandry and Veterinary Medicine, Beijing Academy of Agricultural and Forestry Sciences, Beijing 100097, China; Department of Animal Sciences, Washington State University, Pullman, WA 99164, USA; School of Animal Science and technology, Tarim University, Xinjiang 843300, China; Nongxiao Breeding Poultry Breeding Co., Ltd. Beijing 102400, China; Xingrui Technology Co., Ltd. Hebei 072557, China; College of Animal Science and Technology, China Agricultural University, Beijing 100091, China; College of Animal Science and Technology, China Agricultural University, Beijing 100091, China; College of Animal Science and Technology, China Agricultural University, Beijing 100091, China; College of Animal Science and Technology, China Agricultural University, Beijing 100091, China; College of Grassland Science and Technology, China Agricultural University, Beijing 100091, China; College of Animal Science and Technology, China Agricultural University, Beijing 100091, China

**Keywords:** peafowl, hybridization, introgression, conservation, leucistic plumage

## Abstract

**Introduction:**

The blue peafowl (*Pavo cristatus*) and the green peafowl (*Pavo muticus*) have garnered significant public affection due to their stunning appearance, although the green peafowl is currently endangered. The causative mutation that causes the leucistic plumage of the blue peafowl (also called white peafowl) remains unknown.

**Results:**

In this study, we generated a chromosome-level reference genome of the blue peafowl with a contig N50 of 30.6 Mb, including the autosomes, Z and W sex chromosomes, and a complete mitochondria DNA sequence. Data from 77 peafowl whole genomes, 76 peafowl mitochondrial genomes, and 33 peafowl W chromosomes genomes provided the first substantial genetic evidence for recent hybridization between green peafowls and blue peafowls. We found 3 hybrid green peafowls in zoo samples rather than in the wild samples, with a blue peafowl genomic content of 16–34%. Maternal genetic analysis showed that 2 of the hybrid female green peafowls contained complete blue peafowl mitochondrial genomes and W chromosomes. Some animal protection agencies release captive green peafowls in order to maintain the wild population of green peafowls. Therefore, to better protect the endangered green peafowl, we suggest that purebred identification must be carried out before releasing green peafowls from zoos into the wild in order to prevent the hybrid green peafowl from contaminating the wild green peafowl. In addition, we also found that there were historical introgression events of green peafowl to blue peafowl in 4 zoo blue peafowl individuals. The introgressed genomic regions contain *IGFBP1* and *IGFBP3* genes that could affect blue peafowl body size. Finally, we identified that the nonsense mutation (g.4:12583552G>A) in the *EDNRB2* gene is the genetic causative mutation for leucistic plumage of blue peafowl, preventing melanocytes from being transported into plumage, thereby inhibiting melanin deposition.

**Conclusion:**

Our research provides both theoretical and empirical support for the conservation of the endangered green peafowl. The high-quality genome and genomic data also provide a valuable resource for blue peafowl genomics-assisted breeding.

## Graphical Abstract

**Graphical Abstract:**
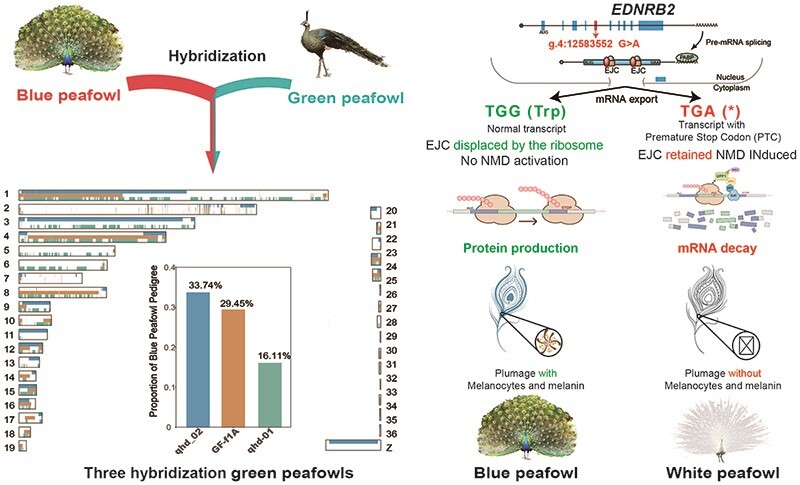

## Introduction

The peafowl (Aves, Galliformes, Phasianidae, *Pavo*), which includes the blue peafowl, *Pavo cristatus* (NCBI:txid9049), and green peafowl, *Pavo muticus* (NCBI:txid9050), are widely regarded as symbols of beauty, nobility, auspiciousness, and good luck in Asian culture [1, 2]. They are extensively used in art, religion, literature, and decoration [3]. Their irreplaceable symbolic significance has led to extensive research in the fields of ecology [4], archaeology, history [5], and genomics [6, 7]. Some studies have found the phylogenetic positions of the peafowl in Phasianidae, but the phylogenetic relationship of the peafowl to the chicken and turkey in the Phasianidae family is strongly controversial [5, 6, 8].

The blue peafowl is distributed in several South Asian countries and widely bred all over the world. It was designated as National Bird of India in 1963 and granted the utmost protection [9]. The green peafowl is larger in size than the blue peafowl [3]. Unfortunately, as the only native peafowl in China, the green peafowl is classified as Endangered on the International Union for Conservation of Nature (IUCN) Red List and categorized as Critically Endangered on China’s Biodiversity Red List [10, 11]. It was once widely and commonly distributed over subtropical and tropical forests in East and Southeast Asia [12]. However, the green peafowl has suffered a sharp population decline over the past 3 decades, and now the number of wild green peafowls is fewer than 500 in scattered habitats [13].

The endangered situation of the green peafowl is mainly attributed to habitat fragmentation due to climate change and agricultural activate, illegal poaching, and so on [10, 11, 12, 14]. However, a critical issue has been overlooked. The blue peafowl and green peafowl are closely related species, and although their wild habitats do not overlap, hybridization between them may still occur due to human breeding or other unknown factors [8, 15, 16]. The hybrid green peafowls are morphologically difficult to distinguish from purebred green peafowls. Hybridization with closely related species mixes gene pools and potentially loses genotypically distinct populations. These phenomena can be especially problematic for endangered species contacting more abundant ones and could contribute to the extinction of endangered species [17–19], which has been demonstrated in red wolf, plains bison, and endangered Java warty pig [20–23].

Introgression is the transfer of genetic material from one species into the gene pool of the other by the repeated backcrossing of an interspecific hybrid with one of its parent species [24]. Introgression is a long-term process, and it plays an extremely important role in species traits and environmental adaptation [25]. A typical finding of introgression is the historical introgression of *Homo sapiens* by Denisovans and Neanderthals to improve the immunity of *H. sapiens* [26]. At present, also due to the lack of genomic data and related studies, we do not know whether there is introgression between blue peafowl and green peafowl and whether introgression has an effect on peafowl traits.

Leucistic plumage blue peafowls (also called white peafowl) are blue peafowl plumage color mutants that are characterized by white plumage, but the eyes are black because they contain melanin [1]. Studies have confirmed that the leucistic plumage trait of blue peafowls conforms to the Mendelian law of autosomal segregation [27]. In chickens and geese, this white plumage mutant individual, caused by leucism rather than albinism, is explained by mutations in multiple genes involved in the differentiation and migration pathway of melanocytes [28, 29].

In this study, we first constructed the first chromosome-level genome of a blue peafowl, including autosomes, sex chromosomes, and mitochondrial DNA by using PacBio HiFi Circular Consensus Sequencing (CCS) and Hi-C sequencing. These genomic resources were utilized to analyze the position of the peafowl in the time-calibrated phylogenetic tree and determine the divergence time between blue peafowl and green peafowl. We also collected and reported datasets of genomic variation in wild and zoo green peafowls and blue peafowls. We aimed to systematically delineate the structure of Asian peafowl genetic diversity and to detect recent hybridization and historical introgression events between blue peafowl and green peafowl. In addition, we investigated the genetic basis of the leucistic plumage phenotype in peafowls and sought to ascertain whether the same gene determining leucistic plumage color in peafowls is the same as those in other birds, as well as the similarities and differences in the molecular mechanisms of the same phenotype. Our research provides not only evidence for understanding the evolution of peafowls but also new insights into saving the endangered green peafowl.

## Materials and Methods

### Sample collection

In this study, we collected 1 female blue peafowl (*P. cristatus*) from China Beijing Zoo for HiFi and Hi-C sequencing. We also collected 2 green peafowls (*P. muticu*s) from China Beijing Zoo, 12 blue peafowls from Hebei XingRui and the China Beijing Zoo, and 6 white peafowls (*P. cristatus*) from Beijing Agricultural Vocational College for whole-genome resequencing, while all resequenced peafowl individuals are artificially bred individuals. In addition, we collected samples of 54 green peafowls and 3 blue peafowls from NCBI databases and CNSA (China Nucleotide Sequence Archive) databases. Among all the peafowl samples from the database, 13 green peafowls were artificially bred individuals, 44 green peafowls were wild individuals, and all blue peafowls were artificially bred individuals. We obtained the plumage follicle tissues of 6 blue peafowls and 6 white peafowls from the peafowl breeding garden in Mentougou District, Beijing, for transcriptome sequencing and combined them with the transcriptome data of 20 blue peafowls published on NCBI for subsequent genome annotation and transcriptome analysis. The white plumage follicle tissue and blue plumage follicle tissue of the same pied peafowl (blue white-flight plumage color) were also used for transcriptome sequencing. In addition, to conduct population verification of genetic mutation of plumage color, we obtained blood samples from 11 white peafowls, 29 blue peafowls, and 1 pied peafowl from Beijing Agricultural Vocational College and the Peafowl Breeding Park in Mentougou District, Beijing. The full sample information can be found in Supplementary Table S1. All of the animals in this study were reviewed and approved by Ministry of Agriculture of China (Beijing, China), Animal Welfare Committee of China Agricultural University (Beijing, China).

### Blue peafowl HiFi sequencing and Hi-C sequencing

The genomic DNA extractions were performed on blood from a single female blue peafowl (*P. cristatus*) individual, using the DNAeasy Blood & Tissue Kit (QIAGEN) following the manufacturer’s instructions. The DNA was quantified using the NanoDrop ND-2000 Spectrophotometer (Thermo Fisher Scientific) with its standard protocol. The extracted DNA was used to construct the PacBio SMRTbell TM library prepared with the Sequel Sequencing Kit 3.0, according to the released protocol from PacBio. The library was processed for PacBio HiFi CCS sequencing on the PacBio Sequel Ⅱ machine by BGI Technologies. A total of 4,085,715,549 bp CCS data were generated. Average CCS length was over 14,503 bp, and the longest CCS read length achieved 44,082 bp.

For Hi-C sequencing, the blood was fixed using formaldehyde for 15 minutes at a concentration of 1%. The chromatin was cross-linked and digested using the restriction enzyme *HindIII*, then blunt end-repaired and tagged with biotin. The DNA was ligated with the T4 DNA ligation enzyme. After ligation, formaldehyde cross-links were reversed and the DNA purified from proteins. Biotin-containing DNA fragments were captured and used for the construction of the Hi-C library. The Hi-C library was sequenced on an Illumina HiSeq X Ten platform (RRID:SCR_016385), producing ∼101.15 Gbp clean data.

### Genome assembly and Hi-C scaffolding

Jellyfish (v2.3.0) (RRID:SCR_005491) was used to obtain a frequency distribution of *k*-mer counting with the clean reads, producing *k*-mer frequency distributions of 31-mers [30]. Then, GenomeScope 2.0 (RRID:SCR_017014) was used to evaluate the peafowl genome size [31]. We used hifiasm to assemble the long reads into contigs by using default parameters [32]. Reads shorter than 8,000 bp were discarded. Then, the yahs pipeline was used to join the contigs into chromosomes [33, 34]. The Hi-C contact map based on the draft chromosomal assembly was then visualized in Juicebox, which also allowed for manual adjustment of the orientations and order of contigs along the chromosomes (Supplementary Fig. S1a). Although the newly released genome of the green peafowl is relatively complete, it is poorly collinear with the chicken genome due to imperfect Hi-C scaffolding [7]. We downloaded the Hi-C sequencing data corresponding to the published green peafowl genome and used yash to improve the chromosome-level genome of green peafowl. The consistency and integrity of the assembled blue peafowl genome (WP-1) were separately assessed using BUSCO (RRID:SCR_015008), based on single-copy orthologs from the AVES (odb10) database. Merqury (v.1.3) (RRID:SCR_022964) was also used to evaluate assembly. [35] To obtain the blue peafowl mitochondrial genome, we assembled *de novo* using NOVOPlasty (v.4.3.1) (RRID:SCR_017335) with default parameters [36]. The mitochondrial sequence length is 16,694 bp (Supplementary Fig. S6).

### Blue peafowl genome annotation

To annotate the repeat content, we first used RepeatModeler2 to predict and classify TEs (Transposable Elements) throughout the genome [37]. The newly predicted families of TEs and tandem repeats were then combined with the Repbase (RRID:SCR_021169) library (RepBase17.01) to annotate repeats using RepeatMasker (v4.0.7) (RRID:SCR_012954). In addition, we used LTR_finder (RRID:SCR_015247) to identify long terminal repeat (LTR) sequences [38].

To predict mRNA-encoding genes in the blue peafowl genome, we performed *ab initio* gene prediction, transcriptome-based gene prediction, and homology-based prediction. For the homology-based predictions, we used protein data from 6 species (*H. sapiens, Mus musculus, P. muticus, Meleagris gallopavo, Gallus gallus, Oxyura jamaicensis*) that were retrieved from the Ensembl (release 64) database and identified candidate coding regions with miniport (v.0.13) [39]. For the transcriptome-based gene prediction, We mapped the collected RNA sequencing (RNA-seq) reads using HISAT2 (v.2.1.0) (RRID:SCR_015530) [40] and assembled the transcriptomes using StringTie (RRID:SCR_016323) [41]. TransDecoder (RRID:SCR_017647) was used for identifying candidate coding regions within transcript sequences. To make *de novo* predictions, BRAKER2 (v.2.1.6) (RRID:SCR_018964) was run to use the homology protein as hints to generate predicted gene models from AUGUSTUS (v.3.4.0) (RRID:SCR_008417) and to train the hidden Markov model (HMM) of GeneMark-ET (v.3.67_lic) [42–44]. EVidenceModeler (RRID:SCR_014659) software was used to integrate the gene set and generate a nonredundant and more complete gene set via integration of the 3 respective annotation files that were assigned different weights (*ab initio* prediction was “1,” homology-based prediction was “5,” and transcriptome-based prediction was “10”) [45]. Finally, PASA (Program to Assemble Spliced Alignments) was used to correct the annotation results of EVidenceModeler for the final gene set [46]. Functional annotation of the protein-coding genes was accomplished using eggNOG-Mapper (v.2) (RRID:SCR_021165) [47], a tool that enables rapid functional annotations of novel sequences on the basis of precomputed orthology assignments, against the EggNOG (v.5.0) database. The protein databases of SwissProt, NR, and Pfam were also used to annotate gene function [48–50]. We also mapped the reference genes to the Kyoto Encyclopedia of Genes and Genomes (KEGG) pathway database and identified the best match for each gene [51]. tRNAscan-SE (v.2.0.11) (RRID:SCR_008637) software was used to search for the transfer RNA (tRNA) sequence of genome [52], with INFERNAL (v.1.1.3) (RRID:SCR_011809) from Rfam to predict microRNAs and small nuclear (snRNA) of the genome [53]. In addition, barrnap (v.0.9) (RRID:SCR_015995) was used to annotated the ribosomal RNA (rRNA) sequence of the genome (Supplementary Fig. S1b).

For mitochondrial genome annotation, we submitted it to the AGORA web tool [54], with the protein-coding and rRNA genes of the *G. gallus* mitochondrial genome (accession number: NC_053,523.1) as a reference.

### Genome synteny and collinearity analysis

We used the MUMmer (v.4.0.0rc1) (RRID:SCR_018171) tool nucmer to perform the pairwise whole-genome alignment with the parameter “-b 400” [55]. The alignments were filtered to keep the one-to-one best hits using delta-filt from the MUMmer package. Unanchored scaffolds were excluded from the alignments. We also performed collinearity analysis using MCscanx (RRID:SCR_022067) with default parameters [56]. The NGenomeSyn (v.1.4.1) was used for synteny visualization [57].

### Phylogenetic tree and divergence time

The protein sequences of 15 species were used to search the orthologs using OrthoFinder2 (RRID:SCR_017118) [58]. The results showed that a total of 17,999 orthogroups were identified in 13 species, of which 10,323 single-copy orthologs were shared among these species. These single-copy orthologs were subsequently converted into coding sequence alignment by tracing the coding relationship using pal2nal.pl (v14) [59]. Then, we construct a phylogenetic tree using RAxML (v.8.0.0) (RRID:SCR_006086) with the following commands: “raxmlHPCAVX -N 100 -b 15,738 -f j -m GTR+G -s sample.phy -n all.tree” [60]. Additionally, the divergence time of all species was estimated and calibrated through the divergence time between *G. gallus* and *Cygus olor, Tympanuchus pallidicinctus* and *Centrocercus urophasianus*, and *Bambusicola thoracicus* and *G. gallus* from the TimeTree database [61]. We then used BASEML to estimate the overall mutation rate with the time calibration on the root node (86.34 Mya (Million Years Ago)). General reversible substitution model and discrete gamma rates were estimated by the maximum likelihood approach under a strict clock. The divergence time was then estimated using MCMCtree [62]. In addition, we also collected the mitochondrial sequences of 8 species from the NCBI and, combined with our assembled mitochondrial sequences, constructed a ultrametric tree of maternal inheritance.

We aligned the genomes of chickens, turkeys, and 2 species of peafowls, with swans as the out-group, to form 5-species whole-genome alignment data by using cactus (v.2.6) with default parameters [63]. For the phylogeny of local alignments of sliding windows among all compared genomes, we partitioned the data into nonoverlapping sliding windows with varying window sizes of 100 kb to reconstruct phylogenetic trees. All windows with more than 75% gaps were removed. RAxML was used to construct maximum likelihood trees from each alignment, and the low consensus trees (bootstrap <50%) were discarded [60].

### Estimates of the effective population size and divergence time

To characterize the historical demography of the wild green peafowl and blue peafowl, a strategy of combining 2 complementary algorithms was employed for cross-validation and comparison. In general, the popular individual genome-based pairwise sequentially Markovian coalescent (PSMC) approach was used to retrieve distant past history, while the SMC++ method based on various algorithms and data exploration were employed to better characterize more recent demography. For all of these analyses, the mutation rate was set to 1.33 * 10^−9^ per site per year estimated for the Indian peafowl and the generation time assumed to be 4 years to scale demographic events in calendar times, following a study on the blue peafowl [5, 64]. The PSMC analysis [65], which utilized LD (Linkage Disequilibrium) information, was conducted on autosomes of the high-coverage *de novo* assembly. We used bcftools to generate autosomal fasta files, respectively, according to a recommendation in the PSMC documentation. The PSMC 100 bootstraps were performed with parameters optimized for birds (N30 –t5 –r5 –p 4+30*2+4+6+10) to determine variance in *Ne* estimates [66]. We also plotted the PSMC by using python script. A recently published approach with higher resolution in the recent past compared to PSMC accuracy, SMC++ [67], was used to predict the demographic history (or population sizes and divergence times) of green peafowl and blue peafowl based on multiple unphased individuals. The short generation time of wild green peafowl and blue peafowl makes possible the reliable and precise estimation of effective population sizes in the recent past using the method of SMC++ (v.1.15.2) [68].

We also used DADI (Diffusion Approximations for Demographic Inference) to infer the divergence time between wild green peafowl and blue peafowl [69, 70]. We simulated 4 models with the same dataset under the 2-population model in DADI independently: model 1 (sym_mig), instantanous size change followed by exponential growth with no population split; model 2 (bottlegrowth_2d), instantanous size change followed by exponential growth, then split with migration; model 3 (bottlegrowth_split_mig), split into 2 populations of specified size, with migration; and model 4 (split_asym_mig), split into 2 populations of specified size, with asymmetric migration. To avoid the effect of selection on the demographic analysis, we focused on noncoding regions and extracted noncoding single nucleotide polymorphisms (SNPs). To avoid the linkage of SNPs, we thinned the noncoding SNPs to 1% and obtained a dataset of 135,873 SNPs [71]. Owing to the unknown ancestral state of each SNP, we folded the frequency spectrum. The projection value was selected based on the strategy of maximizing the number of segregated sites. The starting parameter values for the first round were randomly assigned, and the best parameters obtained after completion of a round were used as the starting parameters for the subsequent rounds. After the convergence of the parameters, we retained the model with the highest log-likelihood as the final simulation result. The parameters of the optimal model were converted into absolute units using the average mutation rate per generation and generation interval. Confidence intervals for the parameters were generated using the Godambe information matrix (GIM) with 100 bootstraps [72].

All blue peafowl and green peafowl populations used for SMC++ and DADI analyses excluded hybrid and introgressed individuals found in subsequent studies.

### Gene family construction and branch-specific positive selection

To define gene families, we used coding sequences of all 15 species and extracted the longest protein for each gene. Gene family size expansion and contraction analysis were performed by CAFE5 (v.5.0.0) (RRID:SCR_005983) [73], and the results from OrthoFinder2 and a phylogenetic tree with divergence times were used as inputs for CAFE5. These expanded gene families were annotated and classified through the analysis of Gene Ontology (GO) and KEGG pathways to further explore the impact of adaptive evolution on peafowl by using Kaobas (v.3.0) [74].

We obtained codon alignments for the single-copy orthologous groups by aligning coding sequence by using MAFFT. Nonsynonymous and synonymous substitution rates (Ka/Ks) were calculated using the codeml program in the PAML (v.4.5) package [62]. We used the branch site model and 2-ratio models to detect signatures of natural selection on coding genes of peafowl. Statistical significance was determined using likelihood ratio tests. Functional annotation of the obtained gene dataset was also performed using Kobas [74].

### Whole-genome resequencing and variant calling

We cleaned the Illumina next-generation sequencing (NGS) raw data to remove adaptors, trim low-quality bases, and remove “N” sites with fastp (v.0.20.0) (RRID:SCR_016962) [75]. Subsequently, clean reads were mapped to our blue peafowl genome using BWA (v.0.7.10-r789) with default parameters [76]. High-quality mapped reads (mapped, nonduplicated reads with mapping quality ≥20) were selected with SAMTools (v.1.3.1) and the following commands: “-view -F 4 -q 20” and “rmdup” [77]. For all samples, we used the “bamqc” module in Qualimap (v.2.2.1) to perform sequencing depth statistics [78]. Only high-quality mapped reads were used for variant calling with GATK (v.4.2.6.1) [79]. BAM files were sorted and marked as PCR duplications with Picard (v.2.27.5) (RRID:SCR_006525). There is no well-annotated SNP and short-indel database for blue peafowl, so it was not feasible to use the Base Quality Score Recalibrator (BQSR) and “IndelRealigner” options of GATK. To carry out variant calling, we used the command “HaplotypeCaller,” which calls SNPs and indels simultaneously via local *de novo* assembly of haplotypes in an active region. Applying the “hard filtering” method, we obtained an initial set of high-confidence SNPs and indels. The parameters of “hard filtering” were set by default: that is, for SNPs, we used QualByDepth (QD) < 2.0, FisherStrand (FS) > 200.0, StrandOddsRatio (SOR) > 10.0, MQRankSum < −12.5, and ReadPosRankSum < −8.0, while for short indels, we considered QD < 2.0, FS > 200.0, and ReadPosRankSum < −8.0. After the initial filtering step, the number of SNPs and short indels became 16,330,028 and 2,153,530, respectively. Notably, the ratios of Transitions (Ts)/Transversions (Tv) to 2.534 with filtering of the raw SNPs showcases the high quality of the SNP call sets. For many downstream analyses, the core set of SNPs and indels was acquired by setting the Minor Allele Frequency (MAF) cutoff at 0.05. We performed SNP and indel annotation according to the blue peafowl genome using SnpEff (v.5.1) (RRID:SCR_005191) [80]. The intergenic region of the genome encompasses about 45.73% of SNPs and 70.0% of indels. Around 1.573% of SNPs are located in the coding sequence, and the nonsynonymous-to-synonymous SNP ratio is 0.508%. In comparison, only 1.081% of indels are found in the coding sequence. In addition, we also used CNVcaller to detect copy number variations in *P. cristatus* for subsequent analysis of the plumage color of white peafowl [81].

### Population genetic structure

We chose the core set of SNPs (MAF greater than 0.05) for additional pruning. PLINK (v1.90b6.12) was used to remove SNPs having a high LD (*r*^2^ ≥ 0.5) within a continuous window of 50 SNPs (step size 5 SNPs) [82], which yielded 3,147,759 SNPs for both analyses. The parameter used in this procedure was “–indep-pair-wise 50 5 0.2.” The results obtained from the above procedure were used to perform principal component analysis (PCA) by using VCF2PCACluster. We used ADMIXTURE software to analyze the population structure of all peafowl samples with kinship (*K*) set from 2 to 5 [83]. Finally, we constructed the unrooted neighbor-joining (NJ) tree by MEGA software (v.11) (RRID:SCR_000667) [84]. The FigTree software (v.1.4.4) (RRID:SCR_008515) was used for visualization. We also constructed the maximum likelihood (ML) tree for all individuals using TreeMix (v.1.13) (RRID:SCR_021636) [85].

### Population genetic diversity

As for calculation of the nucleotide diversity and the linkage disequilibrium decay of each peafowl population, we used 2 software programs, VCFtools (RRID:SCR_001235) and PopLDdecay (v.3.42) (RRID:SCR_022509) with default parameters, respectively [86, 87]. In the study of nucleotide diversity and runs of homozygosity (ROHs), we used the same set of SNPs. Since LD analysis and nucleotide diversity analysis require the number of individuals in the peafowl population to be greater than 1, we removed populations with 1 individual and a hybrid individual. ROHs of each individual peafowl were identified using the homozyg option implemented in the PLINK, which slides a window of 50 SNPs (-homozyg-window-snp 50) across the genome estimating homozygosity. The following settings were performed for ROH identification: (i) required minimum density (-homozyg-density 50), (ii) number of heterozygotes allowed in a window (-homozyg-window-het 3), and (iii) the number of missing calls allowed in window (-homozyg-window-missing 5). The ROHs of each peafowl breed were counted and divided into 4 categories according to the length: 0.5 to 1 Mb, 1 to 2 Mb, 2 to 4 Mb, and >4 Mb [88].

### Phylogenetic analysis of peafowl mitochondria

The BAM alignments were converted to fastq and subsequently used with Mapping Iterative Assembler (v.1.0) to assemble a mitochondrial DNA (mtDNA) consensus sequence. We aligned our 76 mitochondrial genome sequences to a collection of 1 published *G. gallus* mitochondrial genome (accession number: NC_053,523.1). The “TIM3+F+G4” model of nucleotide substitution was selected by comparing the Bayesian information criterion (BIC) scores in jModelTest (RRID:SCR_015244) [89]. A phylogenetic tree was then inferred using ML methods. The ML analysis was performed with the program Raxml (v.8.0.0) [60], and approximate likelihood ratio tests were performed to establish statistical support of internal branches with *G. gallus* as out-group. The mitochondrial haplotypes were built by using DnaSP (v.6.12.03).

### Hybridization analysis between endangered green peafowls and blue peafowls

We used a chromosome painting approach with ancestry-informative sites to validate the delimitation of ancestry blocks detected by the HMM and to visualize patterns of introgression across the blue peafowl. This approach provides a lower level of resolution for ancestry block delimitation but with higher power to classify regions as derived from either parental genome. To identify introgressed intervals in the hybrid individual of green peafowl, we used Loter (v.1.0.1) to infer the haplotype fragments of blue peafowl among all autosomes of green peafowl genomes [90]. In addition, we identified alleles that were differentially fixed in green peafowl and blue peafowl parental populations and had no missing data using the script get_fixed_site_gts.rb. We thinned SNPs to be a minimum of 1 kb apart and mapped these ancestry-informative sites in the green peafowl samples using the scriptplot_fixed_site_gts.rb.

### Introgression analysis

TreeMix (v1.13) was used to confirm relationships between our focal populations and to visualize migration events between populations [85]. We first built the maximum likelihood tree (zero migration events) in TreeMix and then ran TreeMix sequentially with 1 through 10 migration events. We supplied this set of 5,671,362 biallelic SNPs to TreeMix, rooted with *G. gallus*, and estimated the covariance matrix between populations using blocks of 500 SNPs. Three samples (qhd-01, qhd_02, and GF-f1A) were excluded from this analysis because ADMIXTURE indicated that they were likely hybrid individuals. We calculated the variance explained by each model (0 through 10 migration events) using the R script treemixVarianceExplained.R.

To investigate the relationship of the blue peafowl to green peafowl populations, we used AdmixTools (v.7.0.2) (RRID:SCR_018495) for the phylogenetic analysis [91]. We also used 3-population test estimates (*f*3 statistics) to test for admixtures across all peafowl populations. Three-population tests consider population triplets (C, A, B), where C is the test population and A and B are the reference populations. Significantly negative *z* scores (*z* ≤ −3.80, after Bonferroni correction for multiple testing) indicate evidence of test population C containing an admixture of both reference populations of A and B.

The ABBA–BABA test, also known as the D statistic, was used to infer the existence of gene flow between the populations. This analysis of D statistic values was performed using Dsuite software [92], which can calculate D statistics at the genome scale across all combinations of populations with VCF input files. According to the principle of ABBA–BABA test calculation, (P1, P2, P3, O) represent 4 different groups. O is chicken (*G. gallus*) as the out-group. Dtrios was used to calculate the D and *f*4 ratio statistics for all trios of populations in the dataset, with a default value of 20,000 for the jackknife block size. Then, using the Dinvestigate program to calculate the D value for windows containing useable-size SNPs, the sliding window consisted of 2,500 SNPs and a step of 500 SNPs. The locations of the windows with the top 5% D values were obtained, and the genes in these windows were regarded as candidate genes for introgression. Functional annotation of the obtained gene dataset was performed using Kobas [74].

### Histological characterization of the genetic mechanism of leucistic plumage in white peafowls and elucidation of the molecular basis

To understand why white peafowl plumage turns white, we used the peafowl individual resequencing SNP dataset and used VCFtools to calculate *Fst* [86]. We calculated the genome-wide distribution of *Fst* between blue peafowl and white peafowl, using a sliding-window approach (1-kb windows with 200-bp increments). Absolute genetic divergence (Dxy) statistic was calculated to strengthen our results by using custom Python scripts [93]. The bedtools software (v.2.30.0) was used to annotate the selection regions.

For RNA-seq data analysis, the paired-end reads were mapped to the peafowl reference genome (WP-1) using the HISAT2 (v.2.6.1.0) software after quality control [40]. Transcripts were assembled and quantified with the StringTie (v.2.1.1) software [41]. The GFFcompare (v.0.12.6) was used to compare the alternative transcripts among individuals [94]. The differential expression analysis was performed using the DESeq2 (v.1.4.5) package [95]. Ultimately, we enriched candidate genes by the online website KOBAS [74], so as to grasp the functions of selection genes and differential expression genes (DEGs).

To identify the causative mutation that causes leucistic plumage in blue peafowl, we also examined the frequency of all (SNP, indel, and CNV (Copy Number Variation)) mutations within 10 kb before and after the candidate SNP.

DNA was extracted from blood samples with the DNAeasy Blood & Tissue Kit (QIAGEN). The *EDNRB2* gene SNP mutation (g.4:12,583,552 G>A) was genotyped by Sanger sequencing on an ABI 3730xl DNA Analyzer (Applied Biosystems) according to the manufacturer’s instructions. The primer sequences used were as follows: forward primer 5′- TGAAGAAGTGTAAGTCCCGCTG-3′ and reverse primer 5′-AGGTCTCGGTCCCAGTAGTT-3′.

Plumage samples stored in 4% paraformaldehyde were washed in phosphate-buffered saline (PBS), dehydrated with a gradient of alcohol solutions (50% → 70% → 80% → 95% → 100% → 100%), cleared with xylene, and infiltrated with paraffin wax. Samples were embedded in paraffin and sectioned into 4-µm-thick tissue sections. Both the plumage sample sections were stained with hematoxylin and eosin (H&E) and Masson–Fontana. We designed a fluorescence in situ hybridization (FISH) probe using the specific sequence of the CDS region of the *EDNRB2* gene. Sections were incubated with 4′,6-diamidino-2-phenylindole (DAPI) to stain the nucleus and were imaged with a fluorescence microscope.

## Results

### Sequencing, assembly, and annotation of the blue peafowl genome

The blue peafowl genome consists of 78 chromosomes, including at least 30 microchromosomes (76 autosomes + ZW sex chromosomes) according to the karyotype analysis [96]. To assemble a chromosome-level genome for blue peafowl, we generated 38 Gbp of PacBio CCS HiFi reads and 101.15 Gbp of chromatin conformation capture (Hi-C) reads. *K*-mer–based analyses of the Illumina paired-end sequencing reads (51.77 Gbp, 52.71× sequencing depth) estimated the size of the nuclear genome to be approximately 982.21 Mb (Supplementary Fig. S2 and Supplementary Table S1).

The initial assembly was 1.13 Gbp, consisting of 389 contigs with a N50 length of 30.6 Mb, indicating a high contiguity of the assembly. Contigs were then concatenated to the chromosome-level assembly by Hi-C reads. We ultimately obtained 36 pairs of autosomes (9 macrochromosomes, 27 microchromosomes) and ZW sex chromosomes with a genome size of 1.041 Gbp (Supplementary Fig. S3 and Supplementary Table S2). Our genome exhibits a 500-fold and 8-fold improvement, in the scaffold N50 (95 Mb), compared to those of the previously published peafowl genomes reported by Dhar et al. [6] and Liu et al. [1], respectively.

We next evaluated the quality of the genome assembly using BUSCO, Merqury, and Illumina short reads [35, 97]. The complete BUSCO of the blue peafowl genome assembly was 97.1%, indicating a high completeness of the gene space (Supplementary Fig. S4 and Supplementary Table S3). Merqury compares *k*-mers from the assembly to those found in unassembled HiFi reads to estimate the completeness and accuracy. The completeness and quality value (QV) of the genome were 96.34% and 60.47 (>99.99% accuracy), respectively. These results attest to the high accuracy and completeness of our assembly (Table 1). According to our results, repetitive sequences accounted for 13.38% of the blue peafowl genome (Supplementary Table S4), including 10.44% tandem repeats and 7.35% transposable element proteins. Among tandem repeats, long interspersed nuclear elements (LINEs) constitute the majority, accounting for 7.41%. Additionally, short interspersed nuclear elements (SINEs) account for 0.05% and LTRs account for 2.99%. We also identified 354 microRNAs, 308 tRNAs, 151 rRNAs, and 334 snRNAs (Supplementary Tables S2 and S5). For annotation, the collected mRNA sequencing (RNA-seq) data were aligned to the reference genome, and 37,401 putative protein-coding gene models were predicted.

**Table 1:** Quality metrics for the blue peafowl genome assembly generated in the current work and for other blue peafowl and green peafowl genome assemblies published in previous studies.

	Blue peafowl			Green peafowl	
	This study	Liu et al. [1]	Dhar et al. [6]	Zhang et al. [7]	Dong et al. [108]
Sequencing technology	PacBio (Hifi), Hi-C	Illumina NovaSeq 6000, PacBio (CLR), 10X Genomics, Chicoga	Illumina HiSeq, ONT	PacBio, Hi-C	Illumina HiSeq
Level	Chromosome	Scaffold	Scaffold	Chromosome	Scaffold
Total scaffolds	38	726	179,332	115	2446
Scaffolds N50 (Mb)	95	11.4	0.19	75.5	2
Contigs N50 (Mb)	30.6	6.18	0.1	25.4	0.161
Longest scaffold length (Mb)	199.9	38.6	2.5	113.2	13.4
Total sequence length (Gbp)	1.041	1.047	1.025	1.049	1.061
Total number of predicted protein-coding genes	37,401	19,465	23,153	14,935	15,584

### Peafowl genome rearrangement

**Figure 1:**
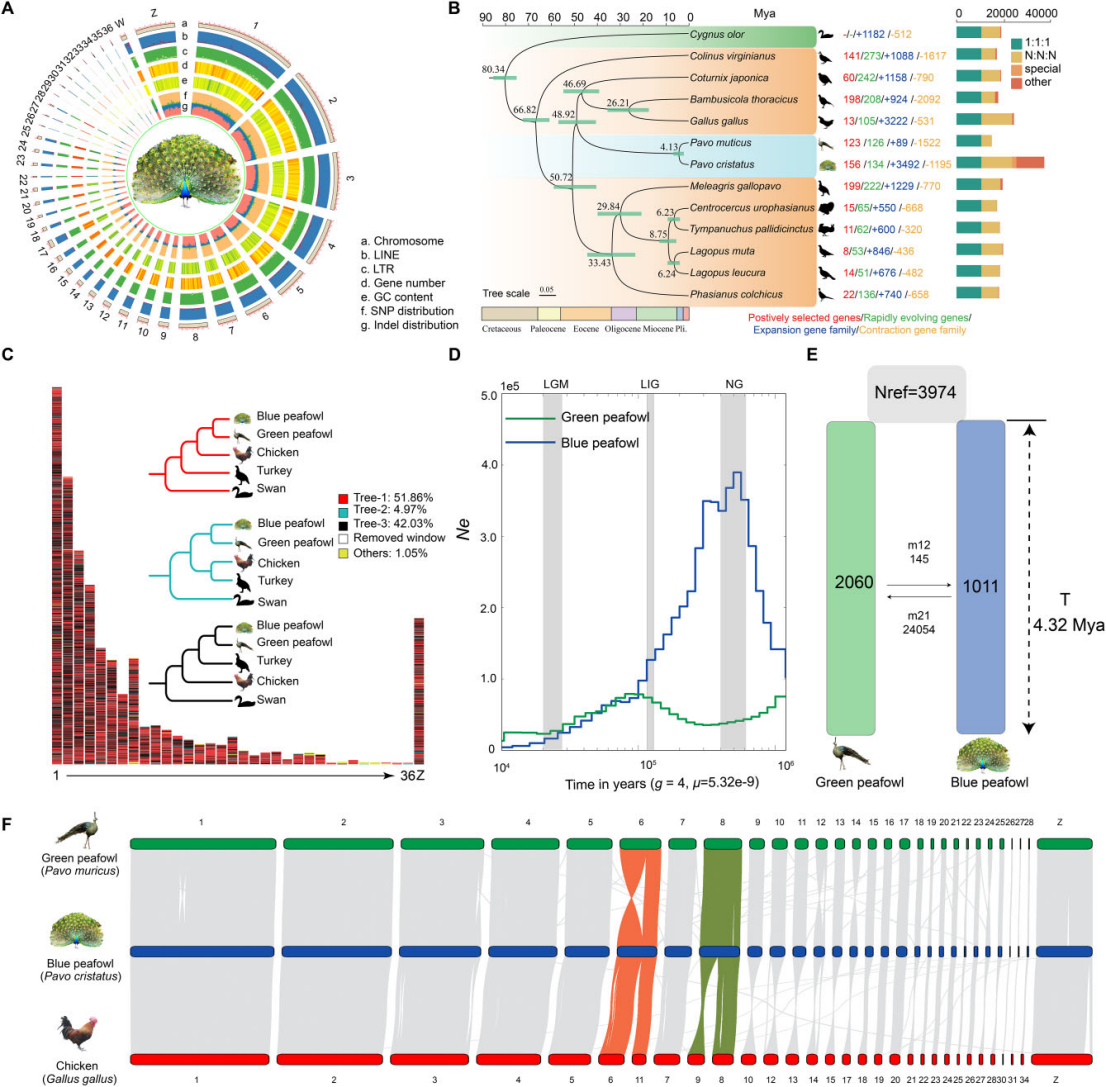
Assembly, composition, evolution and demographic history of the blue peafowl genome. (A) Blue peafowl genomic features. (B) Positively selected genes (PSGs), rapidly evolving genes (RGs), gene family, and phylogenetic and molecular clock dating analysis of the peafowl genomes with 11 other species, based on single-copy orthogroup data. The gray bars at the internodes represent 95% confidence intervals for divergence times. In the histogram, “1:1:1” indicates that the single-copy orthologs are shared by 13 species with 1 copy. “N:N:N” represents any other orthologous group (missing in 1 species). Species-specific shows the specific orthologs in each species. Other orthologs are unclustered into gene families. (C) Distribution of the genomic phylogenetic discordance across each chromosome of blue peafowl by 100-kb sliding windows. Different colors on the genome represent the corresponding phylogenetic topology. (D) Population size history inference of blue peafowl and green peafowl, respectively, across the last glacial maximum (LGM, approx. 26–19 Ka), the last interglacial period (LIG, approx. 132–112 Ka), and the Naynayxungla glaciation (NG, approx. 0.5–0.72 Mya). (E) Best-fit estimates of parameters by using DADI showed the divergence time of blue peafowl and green peafowl. (F) Genome synteny and collinearity among the blue peafowl and chicken.

Synteny and genome size have generally remained stable over the more than 100 million years of modern bird evolution [98–101]. Among the 12% of bird species with documented karyotypes, most have diploid numbers ranging from 76 to 82 [98, 100], and the peafowl has a typical bird karyotype with 2n=78 chromosomes, which is consistent with that of chicken. In this study, we assembled 10 pairs of large chromosomes of 2n=74 in total, which slightly conflicts with the results of the karyotype analysis showing that peafowls have 8 large chromosomes and 30 pairs of microchromosomes of 78 in total (Fig. 1A). The newly improved green peafowl genome has the same number of chromosomes as the blue peafowl.

To understand the similarities between the common peafowl and chicken at the chromosome level, we compared our assembly with the chicken reference genome (*G. gallus* 7.0). Most of the large scaffolds had their counterparts to the macrochromosomes in chicken except scaffolds 6 and 8 (Fig. 1F). Among them, chicken chromosomes 6 and 11 were both aligned to blue peafowl scaffold 6, whereas chromosomes 8 and 9 added up to scaffold 8. We also compared the assembly with the turkey reference genome (Turkey 5.0). Correspondingly, the first 9 scaffolds have their unique counterparts in the macrochromosomes of turkey (Supplementary Fig. S5).

In addition to observing fusion and fission of large chromosomes among blue peafowl, green peafowl, chicken, and turkey genomes, we also observed some microchromosome rearrangements (Fig. 1F). However, due to the GC-richness, specific repeats, high microchromosome mutation rate in birds [96], and lack of cytogenetic supports, determining whether these short scaffolds are bona fide chromosomal rearrangements or simply assembly errors is difficult, and further studies are required in this regard [102].

### Phylogenetic trees resolving the divergence time of blue peafowl and green peafowl

Peafowl is a general name for a type of bird of the order Galliformes and Phasianida, which is a consensus in past studies on both morphology and molecular biology [5]. However, local genomic phylogeny within extant Phasianidae, as well as the phylogenetic relationship of peafowls with turkeys and chickens, has always been controversial [5].

We obtained 17,999 gene families and 10,323 single-copy orthologous genes from blue peafowl, green peafowl, and other 11 species. We generated a whole-genome species tree with the Anseriformes as out-groups by using 5,649,884 sites from the single-copy orthologous gene sets of 13 species, yielding a species tree with 100% bootstrap support for all nodes (Fig. 1B).

According to our results, the Phasianidae family formed a group, with blue peafowls and green peafowls clustered in the Phasianidae branch (Supplementary Fig. S7). The phylogenetic tree built with 4-fold degenerate sites is consistent with the topology of the phylogenetic tree constructed using single-copy orthologous genes and dated the common ancestor of peafowls to approximately 48.92 million years ago (Mya). The blue peafowl and green peafowl diverged 4.13 Mya within the range of divergence (1.95–6.53 Mya). This divergence coincided with the transition between the Miocene and Pliocene epochs, a period characterized by the rapid evolution and replacement of older species by new ones. Additionally, the human ancestor Australopithecus appeared around this time, approximately 4.2 Mya [103]. Several species of Phasianidae, including *Centrocercus urophasianus, Tympanuchus pallidicinctus, Lagopus muta*, and *Lagopus leucura*, also evolved during this period.

Notably, peafowl were found to be closer to chicken than turkey in the Phasianidae family. The ML tree constructed from mitochondrial sequences also shows the same topology. These findings were consistent with the findings of those reported by Dhar et al. [6] and Kimball et al. [104], but there are several studies showing that peafowl is closer to turkey than chicken, including phylogenetic trees constructed using single-copy orthologous genes [1].

To investigate the reasons for these controversies, we constructed the topologies of locally aligned fragments across the whole genomes of 2 peafowls and chickens and turkeys with swan as the out-group. Our results found more than 40% phylogenetic discordance, which was evenly dispersed along the whole reference genome architecture (Fig. 1C).

As is well known, birds survived the Cretaceous–Paleogene extinction event (K-Pg) and underwent multiple species explosions [105]. These species explosion events resulting in incomplete lineage sorting (ILS) have led to a much greater degree of confusion between branches of bird species compared to mammals [106]. The speciation of peafowls coincided with the period of the pheasant family’s major explosion. In addition, gene flow between species may also be responsible for inconsistent species phylogenetic relationships between different datasets [107]. Therefore, we use the *f*4 admixture ratio (*f* statistic; closely related to Patterson’s D), computing *f*(A, B;C, O) for all species that fit the relationship ((A, B), C) in the 4-fold degenerate site tree, with the out-group fixed as swan. Significant gene flow between turkey and the ancestral nodes of peafowl was found (Supplementary Fig. S9). Therefore, we speculate that the high proportion of confusion in the genome-wide phylogenetic trees of peafowls, chickens, and turkey is caused by gene flow and ILS caused by rapid ancestral speciation.

### Green peafowl and blue peafowl have different demographic histories

To understand the demographic history in peafowls, we investigated each species with the PSMC approach and SMC++. PSMC model to infer the local time to the most recent common ancestor (TMRCA) as well as to assess changes in the historic effective population size (Ne) by taking into account whole-genome data from single deep-coverage (>25×) individuals. The SMC++ method was used to reconstruct the population history of the “core” groups of green and blue peafowls found in subsequent studies.

In our results, the Ne changes of wild green peafowl exhibited by the 2 methods are somewhat different during the Last Glacial Period (LGP) period. The result of the wild green peafowl PSMC is consistent with the results of Dong et al. [108], which showed early population decline from 800 to 210 Ka (Kilo annum), followed by a bounce to a peak Ne during the early LGP (ca. 70 Ka) and a more marked decrease (7-fold change) throughout the following part of the LGP (ca. 70–10 Ka) (Fig. 1D). SMC++ analyses showed that during the LGP period, the effective population size experienced a process of first decreasing (ca. 110–30 Ka) and then increasing (ca. 10–20 Ka), and suggested a peak Ne for the green peafowl after the LGP period (ca. 10 Ka). This may be due to differences in the individuals used in the analysis. Such a fluctuating population history broadly agrees with the 2 demographic patterns of most other threatened species, plausibly suggesting a common genetic consequence of late Pleistocene climatic oscillations.

However, blue peafowl showed an entirely different population demographic history. Both PSMC and SMC++ population-based demographic analyses congruently suggested that the Ne of the blue peafowl experienced a dramatic expansion from 500 to 1000 Ka and peaked around 500 Ka before the LGP period and then declined (with a small-scale recovery during the period). We speculate that the blue peafowl may not have adapted to the severe environmental changes during the last glacial period compared to the green peafowl.

We then used the simulation of diffusion approximations for demvographic inference (DADI) to calculate the divergence times between the “core” groups of the blue peafowl and the green peafowl in this study. Among the 4 models defined, model 4 (split_asym_mig) had the highest log-likelihood, indicating the best fit of model to the data (Fig. 1E, Supplementary Figs. S10–S11, and Supplementary Table S6). The demographic estimated using model 4 indicated that blue peafowl and the green peafowl diverged ∼4.32 Mya with a 95% confidence interval (CI) of ±31,180 years, which overlapped with the molecular divergence times of blue peafowl and the green peafowl by MCMCtree. The Ne values of blue peafowl and green peafowl were 1,011 (95% CI ± 10) and 2,060 (95% CI ± 22). Model 4 also indicated that nonsymmetric gene flow existed between blue peafowl and green peafowl after divergence.

### Genetic mechanisms underlying peafowl body size and immune system evolution

Like other birds groups [106], extant Phasianidae species exhibit a large range of body sizes, from *Coturnix japonica* (∼14 cm) at one end of the spectrum to peafowls (>2.3 m in some individuals with 1.5-m tail plumage) at the other [7]. Thus, Phasianidae body size has experienced significant divergence, particularly for the green peafowls with their substantial enlargement in body size.

We detected 123 positively selected genes (PSGs) and 126 rapidly evolving genes (RGs) in the green peafowls (Fig. 1B). Among these genes, *IGFBP4* and *IGF2*, which are related to insulin-like growth factors, may have contributed to the evolution of this trait. Several studies on other vertebrate species corroborate the critical role of *IGF2* in body growth and adult size determination [109]. *IGFBP4* is IGF’s binding partner protein, which is known to cause changed body size in mice and humans [110]. Blue peafowls are slightly smaller than green peafowls, but they are still among the largest species in the pheasant family [6]. We detected that *MSTN* has undergone rapid evolution in blue peafowls. *MSTN* is well known as a muscle growth inhibitory gene. Some cattle breeds (Belgian blue cattle) exhibit extremely exaggerated body size and muscle content due to mutations in this gene [111].

In birds, the rate of sequence divergence in immune-related genes is usually higher than in the other genes primarily because of the coevolution of host–pathogen interactions [112]. We performed enrichment analysis on 555 expanded gene families in the common ancestors of the peafowls and found that 8 genes (*IFNW1, ACTG1, NUP98, IFNA3, KPNA2, ACTB, RNASEL, NXF1*) were enriched in the influenza A KEGG pathway (gga05164), which related to immunity (Fig. 1B and Supplementary Fig. S12) [113]. In addition, we also found that the interleukin family gene *IL7R* and the *CD8BP* gene involved in the adaptive immune response were positively selected in the common ancestor of the peafowls (Supplementary Table S7). Although rapid evolution of immune genes was found in a wide range of birds [106], the number of PSG and RG genes involved in the cytokine–cytokine receptor interaction pathway is highest in blue peafowls (*NODA, IL13RA1, BMP8A, GDF5*) and green peafowls (*CCR10, CD40LG, ACVR2B, CCR7*) (Supplementary Figs. S18, S13 and Supplementary Table S9). It could suggest a key role of this pathway in the evolution of the peafowl immune system. Among these genes, major histocompatibility complex (MHC) genes, interleukin family genes, and NF-κB signaling pathway genes play important roles in immune responses.

### Genome resequencing

**Figure 2:**
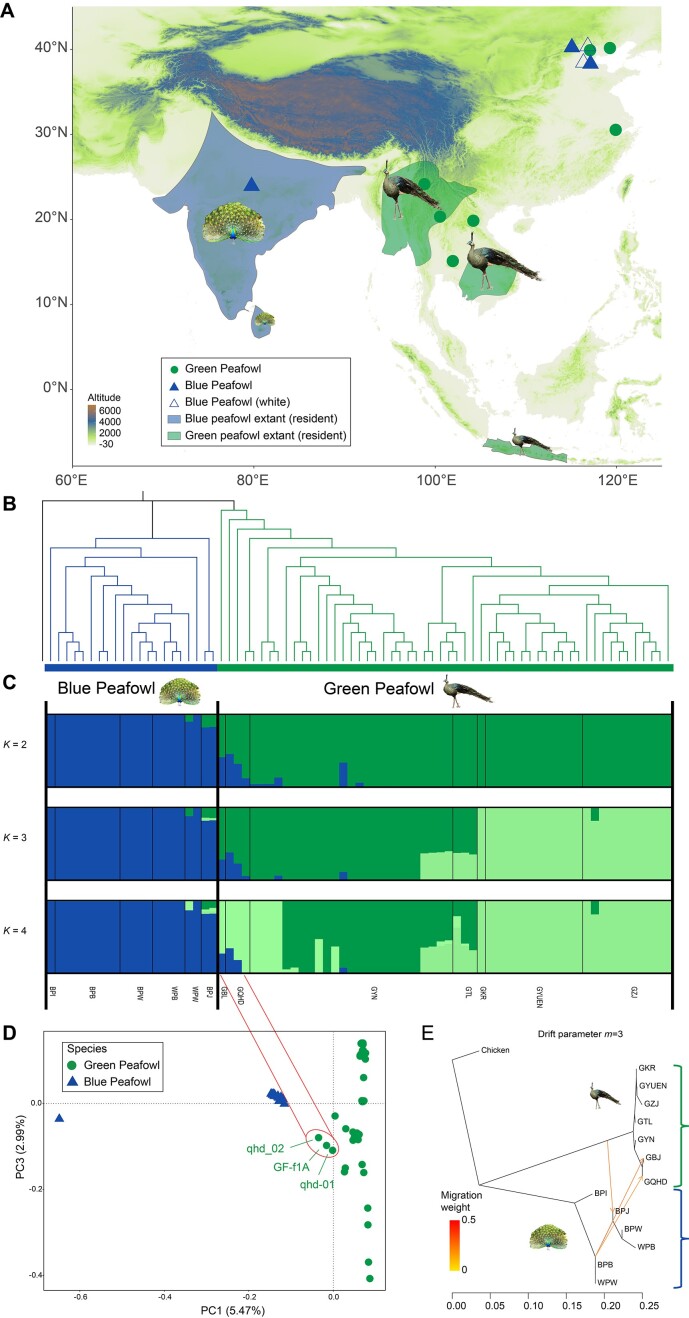
Population genetic structure of blue peafowls and green peafowls. (A) Location of the samples used for this study. A total of 77 peafowls, including blue peafowl (*n* = 22, *P. cristatus*) and green peafowl (*n* = 55, *P. muticus*), were used. (B) A NJ tree of all peafowls estimated based on high-quality autosomal SNPs. (C) Population genetic structure of peafowl. The length of each colored segment represents the proportion of the individual genome from the ancestral populations (*K* = 2–4); population names are at the bottom. (D) PCA plot of peafowl individuals. The 3 individuals in the red circle are the green peafowl that may have hybridized with the blue peafowl. (E) Genetic migration inferred using TreeMix with best draft parameter (*m* = 3), while the migration weight (proportion of admixed population received from source) is indicated on the arrow by the number and color.

In this study, 18 blue peafowl and 2 green peafowl different geographical locations in China were selected for genome resequencing (Fig. 2A). For a more comprehensive analysis of peafowls, we also combined our data with available whole-genome resequencing data for 3 blue peafowl individuals and 54 green peafowl individuals from 7 regions in Asia, giving a total of 77 individuals (Supplementary Tables S10 and S11). Whole-genome resequencing sequences were mapped to our assembled blue peafowl genome (WP-1). After quality control and filtering, 1,399.13 Gbp of high-quality sequences were obtained, with an average of 17.48 Gbp per individual. Across all samples, a total of 12,453,511,109 mapped reads were obtained, with an average depth of 18.53× and an average coverage of 92.07% per individual. After variant calling, a total of 27.78 million variants were obtained, including 16.33 million SNPs and 0.47 million indels. Among all variants, only 1.82% of SNPs and 1.08% of indels are located in exons. Most mutations are located in intergenic regions (Supplementary Fig. S14). In addition, we also collected a rooster as an out-group for subsequent analysis. Of these animals, 76 peafowl individuals were used for a mitochondrial sequence analysis, and 38 female peafowl individuals were used for a W-chromosome SNP analysis.

### Population genetic structure of peafowl using autosomal variants

Genomic SNPs of blue peafowl and green peafowl were used to analyze the population structure of peafowl, as well as to analyze the introgression between blue peafowl and green peafowl.

**Figure 3:**
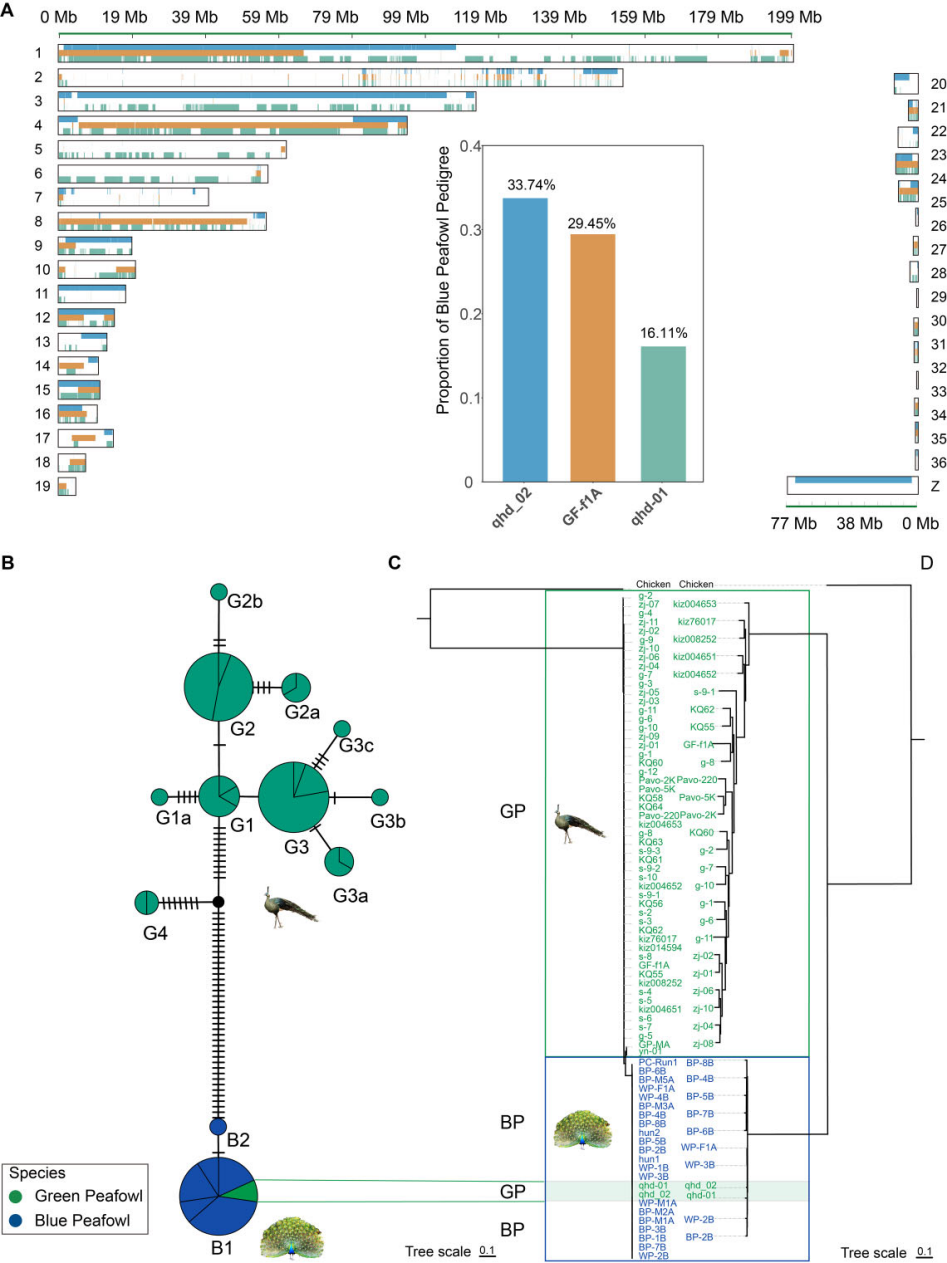
Lineage distribution of hybrid green peafowl individuals in autosomes, W chromosome, and mitochondrial DNA. (A) Genome-wide distribution of blue peafowl haplotypes in hybrid green peafowl individuals. The inset shows the proportion of autosomal blue peafowl ancestry in hybrid green peafowl individuals by using Loter. (B) Median-joining haplotype network from the 76 peafowl individual mitochondrial sequence. (C) Mitochondrial phylogenetic tree of 76 peafowl individuals constructed using the ML method. (D) W chromosome phylogenetic tree of 38 female peafowls constructed using the ML method. BP represents the blue peafowl and GP represents the green peafowl.

The NJ tree of SNPs filtered and trimmed for linkage disequilibrium using chickens as an out-group demonstrated a clear genetic structure with green peafowl and blue peafowl clustered into each clade (Fig. 3B). Admixture analysis (*K* from 2 to 9) of the genomic admixture with 2 population scenarios (*K* = 2) had the best likelihood, and the specimens were divided into 2 groups corresponding to the morphology-based species identification. The blue peafowl (BPB, BPI, BPJ, BPW, WPB, WPW) formed 1 cluster, while green peafowl (GKR, GTL, GYN, GYUEN, GZJ) formed the second cluster (Fig. 3C and Supplementary Fig.  S15). At *K* = 3, 3 clusters were observed; some individuals in the green peafowl (GKR, GYUEN, GZJ) had new clusters. The clustering of green peafowl populations shows clear geographical associations with their sampling locations. Notably, the 3 green peafowl samples (GF-f1A, qhd-01, qhd-02), equally composed of the blue peafowl and green peafowl alleles, were observed from *K* = 2 to 9. PCA analysis also recapitulated these findings. In the first principal component, the blue peafowl and green peafowl clusters were divided along the 2 eigenvectors without overlap. Three green peafowl individuals (GF-f1A, qhd-01, qhd-02) were positioned outside the discovered clusters, which deviated toward green peafowl in the first principal component (Fig. 3D).

Based on species and geographic location, we performed genetic diversity analysis of inferred nucleotide diversity, the linkage disequilibrium (LD) parameters (*r*^2^), and long ROHs for populations with sample sizes >2 (including GYUEN, GZJ, GYN, GTL, GQHD, WPB, BPW, BPB). Among the green peafowl, the GQHD population had the highest genetic diversity, while the GYUEN population had the lowest genetic diversity. BPB populations had the highest genetic diversity and WPB populations the lowest in blue peafowl. We also found that the nucleotide diversity of the green peafowl (π = 1.47^−3^) was lower than that of the blue peafowl (π = 1.99^−3^) (Supplementary Fig. S16). The LD decay rate of the blue peafowl was slower than the green peafowl, and the Yunnan green peafowl showed the fastest LD decay and the smallest *r*^2^, indicating that they had higher diversity in wild environment (Supplementary Fig. S17). The length of the long homozygous segment can reflect the degree of inbreeding of the population. Compared with the peafowl, the ROH length of all peafowl populations is short and the number is small. These results suggests that the degree of inbreeding of the green peafowl is much lower than that of the blue peafowl, which may be due to the long-term wild environment of the green peafowl (Supplementary Fig. S18).

### Maternal phylogenetic analyses of peafowl

Both W chromosome and mtDNA haplotypes represent strong foci in the investigations of Aves. Here, we used complete mitogenomes to construct a haplotype network and rooted tree (Fig. 3C, D). A total of 12 mitochondrial haplogroups (G1, G1a, G2, G2a, G2b, G3, G3a, G3b, G3c, G4 for green peafowl, and B1, B2 for blue peafowl) emerged. We also used 745 SNPs in the W chromosome to construct a phylogenetic tree (Fig. 3E). The evolutionary tree can clearly divide the blue peafowl and green peafowl into 2 branches.

Two green peafowl individuals caught our attention. The phylogenetic tree of all peafowl individuals constructed by mitochondrial sequences showed that 2 green peafowl individuals (qhd-01, qhd_02) were in the blue peafowl clade. This is contrary to the results of autochromatic NJ trees. Combining the results of autosomal admixture and PCA, we speculate that the 2 green peafowl individuals may be hybridized individuals between blue peafowl and green peafowl. The phylogenetic tree of the W chromosome is another strong evidence of studying the maternal genetic relationship of peafowls. We found that the 2 green peafowl individuals are located on the blue peafowl branch on the W chromosome ML tree of 33 peafowl individuals. This further confirms our speculation.

Although the maternal inheritance of the GF-f1A individual was in the green peafowl, we also assumed that it is also a hybrid individual because the autosomes show a similar ancestry distribution to the other 2 individuals.

### Hybrid green peafowl individuals in the zoo

In the wild, the habitats of the blue peafowl and the green peafowl do not overlap (Fig. 2A) [3, 114]. The number of wild green peafowls is extremely rare, with reports indicating that their total population is fewer than 500 [11]. Although blue peafowls are not reproductively isolated from green peafowls, no hybrid green peafowls have been reported in the wild. Through phylogenetic tree analysis of autosomal, mitochondrial, and W chromosomes sequence, we discovered 3 putative green peafowl individuals hybridizing blue peafowls and green peafowls. Next, we will further verify this hybridization event and analyze the proportion of blue peafowl blood in these samples.

Migration events between green peafowl and blue peafowl populations were estimated using TreeMix and constructing ML phylogenetic trees. When the migration event was set to an optimal value of 3 (*m* = 3), gene flow from blue peafowl and green peafowl occurred (Fig. 2E). To investigate the relationship between the 3 green peafowl individuals and the blue peafowl, we selected core groups of green peafowl respectively based on the genetic structure analysis using ADMIXTURE and a phylogenetic analysis using *f*3 statistics (Supplementary Tables S12 and S13). Then, we performed D statistics and *f*3 statistics for all individuals based on SNP frequency differences. In D statistical analysis, we set (P1, P2, P3, O) as (green peafowl individuals, putative hybrid individuals, blue peafowl individuals, chicken). The D statistics showed significant introgression events (*z* > 3, *P* < 0.001) from all putative hybrid individuals. The *f*3 statistics and D statistics also confirmed that the 3 green peafowl individuals shared the most derived polymorphisms with green peafowl. These results corroborated the PCA and ADMIXTURE results that demonstrated the existence of hybridization between blue peafowl and green peafowl.

**Figure 4:**
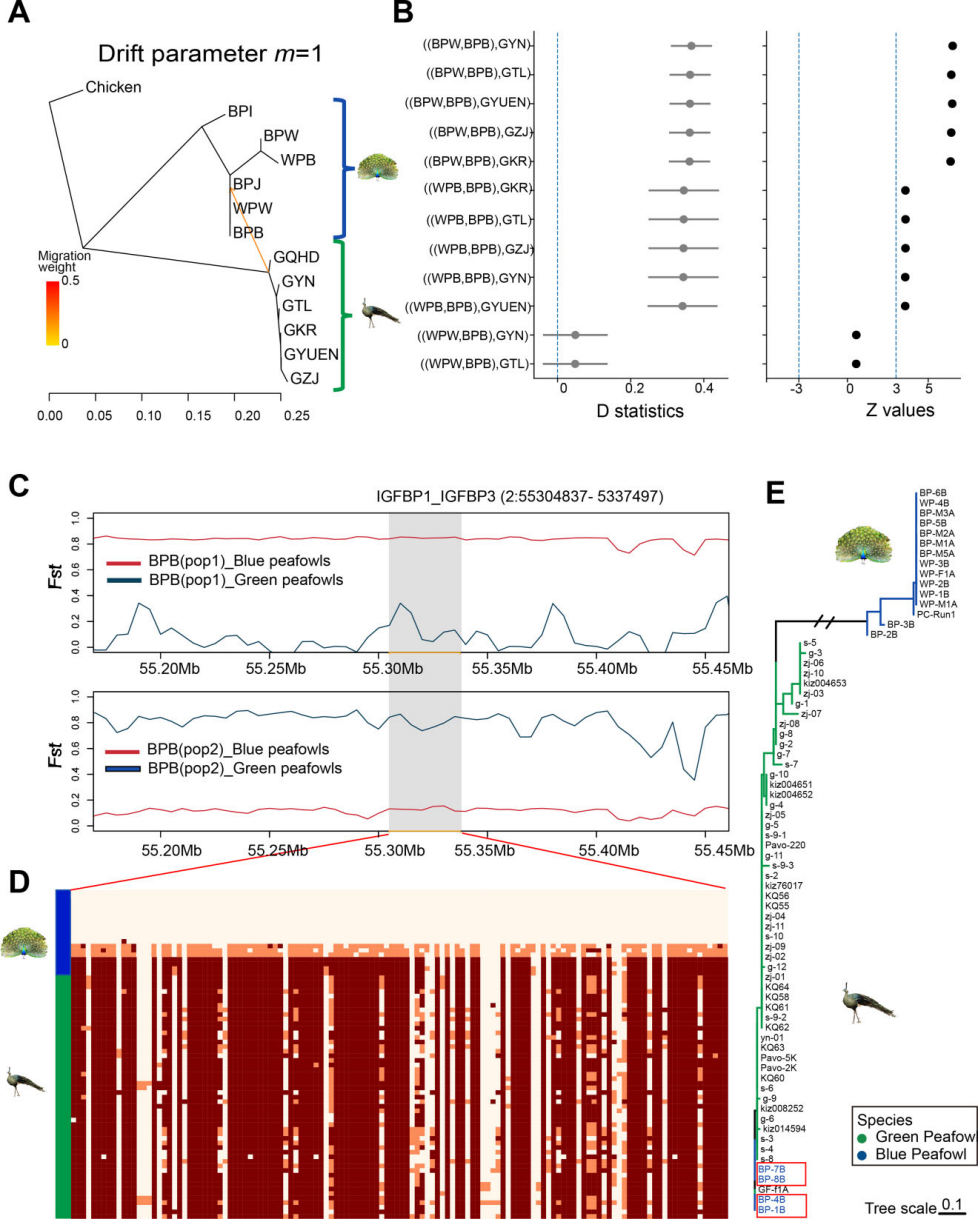
Introgression analysis of peafowl population. (A) Genetic migration inferred using TreeMix (*m* = 1). (B) D statistics for introgression between BPB blue peafowl population and green peafowl population. (C) *Fst* between BPB blue peafowl individuals and the green peafowl populations. The BPB blue peafowl samples were divided into 2 groups based on *Fst* values. BPB (pop1) includes BP-1B, BP-4B, BP-7B, and BP-8B; BPB (pop2) includes BP-2B, BP-3B, BP-5B, and BP-6B. The blue line represents *Fst* between BPB and green peafowl, and the red line represents *Fst* between BPB and blue peafowl. The gray region indicates the location of the *IGFBP1* and *IGFBP3* gene regions. (D) Introgression plot of the blue peafowl contacted based on shared allele SNPs in *IGFBP1* and *IGFBP3* gene regions across chromosome 2 (from 55,304,837 to 55,337,497 bp). (E) Phylogenetic tree of 77 peafowl individuals constructed by 109 SNPs in *IGFBP1* and *IGFBP3* gene regions using the ML method. The blue peafowl individuals in the red box have introgression in this region.

Using the core populations of the 2 species as a reference, we showed that hybrid green peafowl individuals share allele SNPs and haplotype region with the blue peafowl (Fig. 4A and Supplementary Fig. S19). Two individuals (qhd-01, qhd_02) had similar shared allele profiles and another individual (GF-f1A) differed from them, possibly due to differences in genetic background due to geographic location. We also used the same sample to calculate the proportion of ancestry of blue peafowl among the 3 hybrid individuals (Fig. 4A and Supplementary Fig. S20). Among them, the proportion of ancestry of blue peafowl in the qhd_02 individual accounted for the highest (33.74%), which indicated that this individual was a hybrid individual of 1 or 2 generations of blue peafowl to green peafowl. Although the mtDNA of the GF-f1A individual is in the green peafowl clade, the proportion of ancestry of blue peafowl in its genome reached 29.45%. Phylogenetic relationships inferred from ML trees constructed using mitochondrial SNPs and W chromosome SNPs (Fig. 4B–D) further supported these findings, indicating distinct clusters of green peafowl individuals and blue peafowl, whereas 2 green peafowl individuals (qhd-01, qhd_02) were located in the blue peafowl branch.

### Historical introgression regions influence blue peafowl body size and immunity

TreeMix analysis of all individuals showed not only the gene flow from blue peafowl to green peafowl but also the gene flow from green peafowl to Beijing blue peafowl (Fig. 2E). Therefore, blue peafowls and green peafowls may have historical introgression events. We removed the individuals identified as hybrids, and the calculated TreeMix showed the gene flow from green peafowl to Beijing blue peafowl occurred when the migration event was set to 1 (*m* = 1) (Fig. 4A). To further analyze introgression events, we performed *f*3 statistics and D statistics based on SNP frequency difference. Both methods showed significant introgression events (*z* > 3) (Fig. 4B).

The introgression region was further defined by identifying 267 genes in the top 5% D value window positions and presuming these to be candidates for introgression (Supplementary Fig. S21 and Supplementary Table S14). The functional categories that were enriched for significantly introgressed genes mainly included chemical synaptic transmission (GO:0,007,268), neurotransmitter receptor activity (GO:0,030,594), neuron projection (GO:0,043,005), and GABA-gated chloride ion channel activity (GO:0,022,851). These GO items are all related to neural responses, which may improve the stress response and survival ability of blue peafowls in the complex environment. The KEGG annotation showed that the largest groups of introgressed genes involved neuroactive ligand–receptor interaction (gga04080) (Supplementary Fig. S23). It also demonstrates the integral role of the introgressed gene in the blue peafowl’s nervous system and rapid responses for survival in the complex environment.

Among these genes, *IGFBP1* and *IGFBP3*, members of the insulin-like growth factor-binding protein (IGFBP) family, were highlighted due to their association with body size [115, 116]. These 2 genes are annotated to cellular senescence in KEGG and are involved in cell proliferation. The degree and direction of introgression in the BPB blue peafowl were further detected by calculating the *Fst* between BPB blue peafowl individuals and other blue peafowl, BPB blue peafowl individuals and green peafowl individuals, respectively, based on the results of D statistic analysis (Fig. 4B). We obtained a region located on chromesome 2 (from 55,304,837 to 55,337,497 bp), which contained the candidate genes *IGFBP1* and *IGPBP3*. In the region, the BPB pop1 (BP-1B, BP-4B, BP-7B, BP-8B) and green peafowl were poorly differentiated (*Fst* = 0), and BPB pop2 (BP-2B, BP-3B, BP-5B, BP-6B) and blue peafowl were highly differentiated (*Fst* = 0.89) (Fig. 4C). An ML tree was constructed using *IGFBP1* and *IGPBP3* gene sequences (Fig. 4E). In the ML tree, BP-1B, BP-4B, BP-8B, and BP-7B clustered with the green peafowl branch, and other blue peafowl formed separate branches. Haplotypes in this region also showed similar results (Fig. 4D, E). We also found several other genes currently known to be associated with body size in the introgressed region (*IGF2BP3, ISPD, MEOX2, GLI3*, and *MC4R*) (Supplementary Fig. S22).

In addition, we retrieved several immune response–related genes (*IL6, IL12B, IL25, NFATC1, DROSHA*, and *CUL1*) in introgressed regions that may help blue peafowl fight disease. Among all immune genes, *IL-6* gene is involved in multiple immune pathways and is a chicken heat shock protein [117, 118]. Studies have found that *IL-6* is involved in the innate immunity of poultry diseases such as Newcastle disease (ND) [119].

### Nonsense mutation in *EDNRB2* gene creates leucistic plumage individuals in blue peafowls

**Figure 5:**
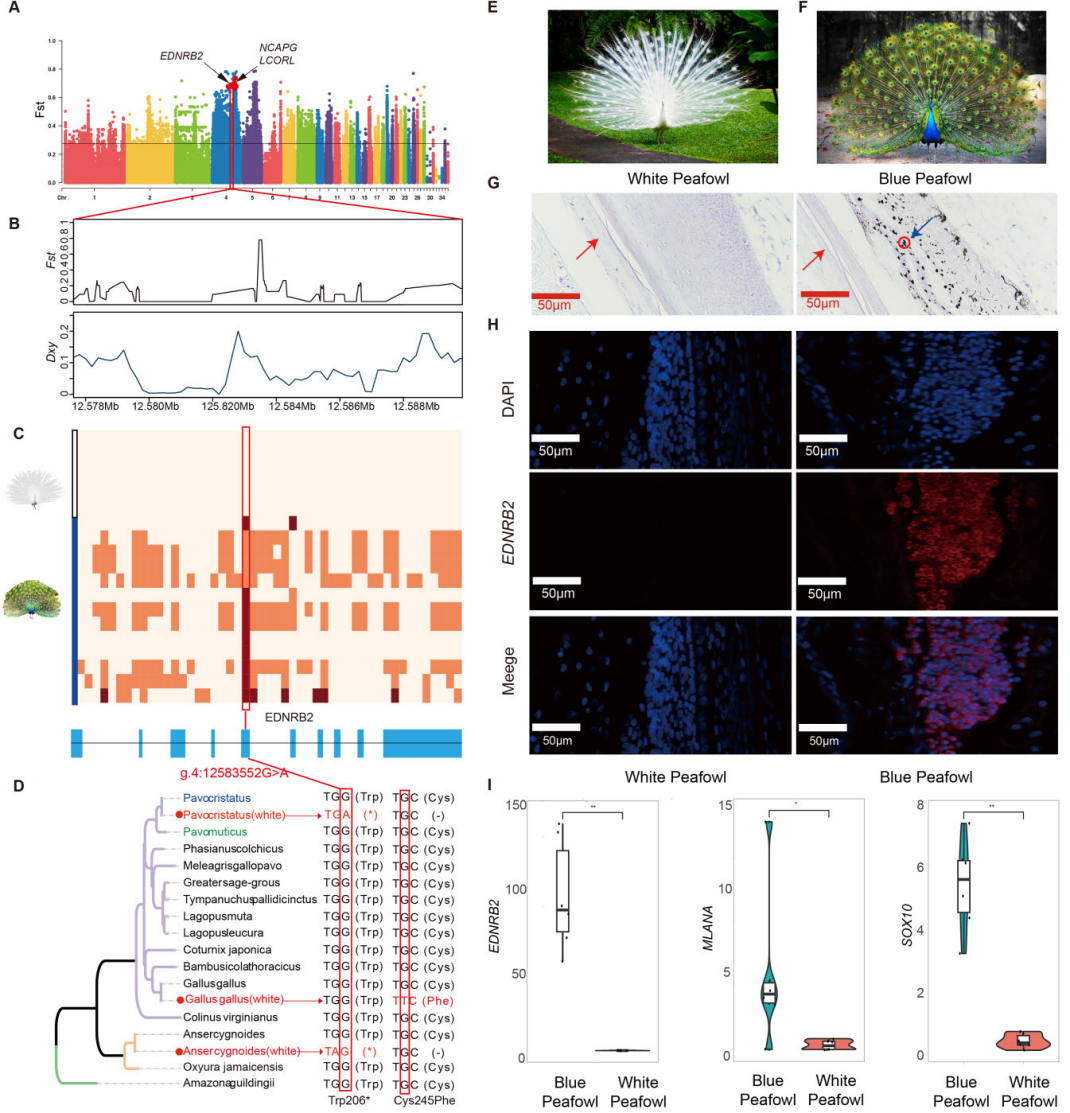
White peafowl leucistic plumage phenotype characteristics and molecular mechanism. (A) Whole-genome scan with *Fst*. The red arrow indicates the genome position of the strongest selective signal for the leucistic plumage phenotype. (B) *Fst* and Dxy corresponding to the leucistic plumage selective sweep on chromosome 4, which encompasses the *EDNRB2* gene. (C) Plot of the haplotype structure of SNPs around the *EDNRB2* gene in all-white peafowls and blue peafowls. Nucleotides with brown box represent blue peafowl homozygote genotypes, and those with an orange box represent heterozygote genotypes. The plot also shows the genetic structure of *EDNRB2*, and the blue box is the CDS region. The position of the red line indication is the nonsense mutation of the leucistic plumage phenotype (g.4:12583552G>A), which is located in the fifth CDS region. (D) The convergence evolution of *EDNRB2* codons in different leucistic plumage species comparison. The first convergent site is Trp206*(Stop codon). The white peafowl has a nonsense mutation at this position. White geese have a 14-bp insertion before the position, so that the codon is turned to be a stop codon in this position. The second convergent site is Cys245Phe, which only occurs on the leucistic plumage chickens. (E) A white peafowl with white plumage and black eyes. (F) A blue peafowl with black and blue plumage. (G) Micrographs of plumage sections from white and blue peafowls stained with Masson–Fontana. The red arrow is the plumage tube wall. Blue arrows are melanosome and melanocytes. (H) FISH was performed to observe the cellular location of *EDNRB2* in the plumage tube. Nuclei were stained with DAPI. Scale bar = 50 µm. (I) *EDNRB2* and melanocyte maker genes (*SOX10* and *MLANA*) are expressed in white peafowl and blue peafowl plumage, respectively. Genetic expression is measured by transcripts per million (TPM). Data are indicated as mean ± SEM (*n* = 3), ns *P* ≥ 0.05, *P < 0.05, **P < 0.01.

As early as 1868, Darwin reported the white peafowls and pied peafowl (blue-and-white flight plumage color), the mutant individuals of the blue peafowl’s plumage color, which shows that the white peafowl appeared at least 150 years ago (Fig. 5E, F) [120]. Under artificial breeding, white peafowls have reached a state of self-sustaining population. As a striking ornamental bird, research on the genetic mechanism of plumage color in white peafowls has never ended [1]. Studies have reported that the recessive allele that controls the leucistic plumage of peafowls is located on an autosomal chromosome, and its inheritance conforms to Mendel’s law of segregation [27]. However, the molecular mechanism of white peafowl plumage color has not been elucidated due to technical limitations [1].

In this study, we compared 11 blue peafowl samples and 5 white peafowl samples from different geographic regions to identify the genomic region responsible for the emergence of mutated white peafowl individuals. We used the *Fst* method to search for the causal mutations. The most significant region was located on the 12- to 13-Mb interval of chromosome 4 (Fig. 5A, B and Supplementary Table S15). The analysis, based on absolute genetic divergence (Dxy) and Tajima’s D, supported a strong selective signal in this region. Through genotypic analysis of 159 mutations (SNP, indel, and CNV) in this interval, we found that the only polymorphism completely associated with leucistic plumage in blue peafowl was a nonsense mutation site located in the *EDNRB2* coding region (g.4:12,583,552 G>A) (Fig. 5C and S24). *EDNRB2* is an important key gene in the formation of bird plumage color. *EDNRB2* is one of the receptors for Endothelin genes (EDNs), which are strong mitogens for melanoblasts. Kawasaki-Nishihara et al. [121] suggested that EDN3–EDNRB2 signaling is required for normal melanoblast migration in *Xenopus* embryos on the basis of *in vivo* experiments. It does not participate in the melanin synthesis pathway but regulates the differentiation, proliferation, and migration of melanocytes [122, 123]. Therefore, we speculated that this incorrect genetic information leads to RNA degradation, known as NMD (nonsense-mediated mRNA decay) [124]. The degradation of *EDNRB2* mRNA inhibits the differentiation, proliferation, and migration of peafowl melanocytes, resulting in the white peafowl individuals with white plumage and black eyes.

To confirm this assumption, we analyzed the genotypes of 11 white peafowls, 29 blue peafowls, and 1 pied peafowl. This SNP showed homozygous or heterozygous genotypes (G/G or G/A) in all blue peafowls and heterozygous genotypes (G/A) in pied peafowl, while all white peafowls were homozygous for the mutant (A/A) (Supplementary Table S16). These results indicate that the leucistic plumage phenotype emerged as a result of the nonsense mutation in the *EDNRB2* gene, and the white allele (A) is shown to be an incompletely dominant. RNA-seq data of 3 blue peafowl and 3 white peafowl plumage found that there was no expression of the *EDNRB2* gene in the white peafowl plumage, while the gene was highly expressed in the blue peafowl plumage. FISH experiments on this tissue showed the same results (Fig. 5H). In addition, we also found that the melanocyte marker genes (*SOX10* and *MLANA*) are not expressed in white peafowl plumage [125] but are highly expressed in blue peafowl plumage (Fig. 5I and Supplementary Fig. S25). The *MLANA* gene is not expressed at all in white plumage follicles of the pied peafowl (G/A), while it is expressed normally in blue plumage follicles of the same pied peafowl (Supplementary Fig. S26). The formation and deposition of melanin mainly occur on the amyloid fibers of melanosomes. Masson–Fontana staining found no melanin and melanocytes in white peafowl plumage tissue (Fig. 5G). The above results indicate that melanocytes do not exist in white peafowl plumage. This verified our conjecture that the nonsense mutation of the *EDNRB2* gene caused the NMD reaction in white peafowls, and the mRNA of the *EDNRB2* gene was degraded during translation, thus preventing melanocytes from being transported into plumage tissue.

### Convergent selection for the leucistic plumage in geese, chicken, and peafowls

The leucistic plumage trait in birds represents a common phenotypic convergence, particularly observable in the poultry breed [126, 127]. In these species, various colorful birds have been domesticated, leading to the emergence of leucistic plumage individuals due to the domestication syndrome [128]. However, the molecular mechanisms underlying this leucistic plumage trait differ across species. For instance, in waterfowl, leucistic plumage in duck results from alternative splicing of the *MITF* gene [129, 130], whereas in geese, it is caused by mutations in the *EDNRB2* gene [29]. In chickens, dominant leucistic plumage is governed by the *PMEL17* gene [126], while tyrosine-independent leucistic plumage is controlled by the *EDNRB2* gene [28]. Notably, both leucistic plumage chickens and geese regulated by the *EDNRB2* gene exhibit predominantly white plumage across their bodies but possess black eyes.

In this study, to explore the underlying molecular regulatory mechanism of this phenotypic convergence, we compared the *EDNRB2* gene sequences in 15 species of birds, including leucistic plumage chickens and leucistic plumage geese. In the case of Minohiki chickens, a nonsynonymous mutation (G>T) associated with the leucistic plumage phenotype was identified in the coding region (CDS) of the *EDNRB2* gene [28]. This mutation leads to a functional defect in *EDNRB2*’s ability to bind to EDN ligands, which in turn interferes with melanocyte differentiation, proliferation, and migration (Fig. 5D). In the Gang geese, a 14-bp insertion caused a frameshift mutation in the gene’s coding region, resulting in a premature stop codon (TAG) [29]. This insertion also triggered the NMD mechanism, leading to the absence of detectable mRNA in white geese individuals.

Although these findings highlight the involvement of the same causative gene (*EDNRB2*), the mutations responsible for leucistic plumage in the 3 species occurred independently after the species diverged. Notably, the mRNA expression of *EDNRB2* in geese is also lost due to nonsense mutations caused by frameshift mutations, thus indicating that the molecular mechanisms of leucistic plumage in peafowls and geese are very similar.

## Discussion

In this study, we report the first chromosome-level *de novo* genome assembly for the blue peafowl with the 95-Mb scaffold N50 of the assembly, which was substantially higher than those obtained in previous studies and the green peafowl genome [1, 5, 7]. We obtained more pairs of macrochromosomes than in the karyotype study of the blue peafowl, which had only 8 pairs of macrochromosomes [96]. The genome synteny and collinearity analysis with chicken and other birds and the evaluation of various algorithms prove that the current blue peafowl genome assembly is of high quality, with consistency, accuracy, and completeness. Although we achieved partial chromosome-level assembly, due to the GC-richness, specific repeats, high microchromosome mutation rate in birds [131], and lack of cytogenetic support, the peafowl microchromosomes and W chromosome require a combination of different data sources, including optical mapping and linked reads to improve the assembly quality [132, 133]. In general, this chromosome-level genome of blue peafowl strongly supports the subsequent comparative genomic and population structure analysis. The karyotypes of birds are relatively conserved, especially in the pheasant family [99, 134]. This pattern was confirmed in our analysis of the conserved synteny between turkey and chicken. We detected only chromosome fusions on chromosomes 6 and 8.

Regarding the relationship between peafowl and chicken and turkey, there are 2 views: (i) peafowl is closer to turkey than chicken [1, 135], and (ii) peafowl is closer to chicken than turkey [5, 6, 136]. To resolve previous inconsistencies concerning peafowl phylogenetic relationships, we reconstructed 3 phylogenomic trees from one-to-one ortholog datasets, 4-fold degenerate site datasets, and mitochondrial sequence datasets, respectively. All phylogenetic trees supported that peafowl was closer to chicken than turkey.

We found that the appearance of peafowls (∼48 Mya) coincided with the rapid speciation of pheasants based on multiple species fossil time correction or site spectrum. Rapid speciation has occurred many times during the evolution of birds, and these events have resulted in a large number of ILS among bird species. Combined with the timing of the peafowl’s emergence and a number of phylogenetic inconsistencies across the genome, we believe that ILS is one of the reasons for the huge controversy over the phylogenetic relationships of peafowls with chicken and turkey. Additionally, we also found evidence of gene flow between the ancestral nodes of peafowl and turkey, which indicates that gene flow also contributes to the difficulties in reconstructing the phylogenetic tree among these 3 species.

Our results indicated that blue peafowl and wild green peafowl have different population histories. The wild green peafowl experienced rapid population expansion, followed by a dramatic population decline during the LGP, which consistent with the results of Dong et al. [108]. The most other threatened species experienced a similar history of group dynamics [5, 66, 137–139]. The effective group size of blue peafowl is higher than that of blue peafowl before LIG. Moreover, the effective group size of the blue peafowl gradually became smaller after the LIG, which is very similar to the historical dynamics of domestic chickens [140]. The different group histories of the 2 species may be an important factor in the current divergent fate of the 2 species. Although the wild green peafowl experienced a significant decrease in genetic diversity in the past 50 years [108], the genetic diversity of wild green peafowl populations is still significantly higher than that of captive blue peafowl.

Habitat loss and fragmentation have long been considered the primary cause for biodiversity loss and wild animal extinction worldwide [141]. Over the past 3 decades, the endangered wild green peafowl, as the only peafowl native to China, has experienced sharp population declines and faced the problem of habitat fragmentation [10, 11]. In order to respond to the Convention on Biological Diversity and save the endangered wild green peafowl [142], the Chinese government has issued a number of protection policies, including banning poaching and captive breeding [13, 16]. The wild green peafowl can hybridize with blue peafowl and produce fertile offspring that can backcross with ancestral species [143]. However, since the habitats of the wild green peafowl and wild blue peafowl do not overlap, and the number of green peafowl is extremely small, no hybrid green peafowls have been reported in the wild [11, 114]. Some researchers in Southeast Asia have recognized the importance of preventing blue peafowls from interbreeding with wild green peafowls to protect the endangered green peafowl. They have suggested that the breeding of blue peafowl near the distribution area (including the potential distribution area) of the wild green peafowl should be prohibited. When blue peafowls and green peafowls are kept in captivity in zoos, their geographical isolation vanishes, significantly increasing the likelihood of interbreeding. In this study, through autosomal and mitochondrial analysis of peafowl populations and validation using multiple methods, we found 3 green peafowl individuals hybridizing with blue peafowl. The proportion of ancestry of blue peafowl among the 3 hybrid green peafowl individuals is less than 50%. Obviously, these 3 peafowls have undergone backcrossing after hybridization. Moreover, consistent with the description by Du et al. [143], hybrid green peafowls were indistinguishable from purebred green peafowls in appearance. Over the years, for endangered species, captive breeding for reintroduction is potentially an important measure for localized population restoration [143]. Yet, the peril to endangered animal conservation posed by hybridization with closely related species in captivity and their subsequent unregulated release has been disregarded, such as red wolf (*Canis rufus*) hybridizing with coyote (*Canis latrans*), bison (*Bison bison*) with domestic cattle (*Bos* spp.), or banded pig (*Sus scrofa vitattus*) with the endangered Java warty pig (*Sus verrucosus*) [20–23]. Hence, we advocate for segregating the habitats of green peafowls and blue peafowls during artificial breeding to deter hybridization. At the same time, we suggest that purebred identification must be carried out before releasing green peafowls to prevent genetic contamination.

In addition to recent hybridization, we also found historical introgression events between blue peafowl and green peafowl, with blue peafowl having gene flow from green peafowl. Our results showed introgression from green peafowl to blue peafowl, which contained 267 candidate protein-coding genes, including genes related to neurodevelopment, cell signaling, transcription, translation, and skeletal development. The sequence of *IGFBP1* and *IGFBP3* had the strong signals among these genes. Both genes that conserved in birds are members of the IGFBP family and encode a protein with an IGFBP domain and a thyroglobulin type I domain [115]. The IGFBP family mediates IGF effects by enhancing or dampening IGF signaling. This occurs by either increasing IGF-receptor affinity, physically sequestering it to prevent receptor binding, or extending IGF’s half-life in circulation [116]. Additionally, many IGFBPs can act independently to induce cellular activity [144]. A large number of studies have found that *IGFBP3* affects growth traits in common domestic animals, such as pig, cattle, and sheep [145–147]. Moreover, the IGFBPs system is highly conserved in chicken and is involved in the regulation of egg production, growth, and carcass traits. *IGFBP3* participates in myogenic cell proliferation and myoblast differentiation, and the SNPs in the *IGFBP3* promoter region were significantly associated with body weight, breast muscle weight, and leg muscle weight [148]. The genes (*IGF2BP3, ISPD, MEOX2, GLI3*, and *MC4R*) related to body size in blue peafowl were also found to have introgression areas from green peafowl. The size of the peafowl is larger in the pheasant family, and the size of the green peafowl is even larger than that of the blue peafowl. These body size–related genes may be an important reason for the current larger size of the blue peafowl, but whether it is a factor affecting the viability of the blue peafowl needs further research. Immune genes, such as interleukins *IL6, IL12B*, and *IL25*, located in the introgressed regions, play a crucial role in the immune response to most low pathogenic avian influenza strains [103, 149]. These genes are highly expressed in poultry infected with the pandemic H5N1 influenza virus, with *IL6* being directly linked to host morbidity and mortality [103]. Thus, the presence of these genes in the introgressed regions is likely due to adaptive introgression. Similar cases of immune gene introgression have been observed in cattle and chickens, significantly enhancing population survival [150, 151].

Molecular genetic studies in a variety of organisms highlight the repeatability of phenotypic change by alterations in the same genes [152, 153]. Among vertebrates, this trend is especially pronounced in studies of pigmentation diversity. One dramatic example of this repeatability is the dilution of the coloration typical of the Dun phenotype, which displays very similar microscopic and macroscopic features in Perissodactyla horses and donkeys [154]. *TBX3* is responsible for the Dun pattern of pigmentation in both species. The causal mutation of the non-Dun phenotype in donkeys is a 1-bp deletion with a probable regulatory effect. Similarly, in horses, the non-Dun phenotype is explained by 2 deletions with regulatory effects [155]. In another study, Lopes et al. [156] provided evidence that the yellow and red plumage pigmentation of canaries and finches is caused by C(4)-oxygenation of carotenoids by the cytochrome P450 enzyme *CYP2J19*. This chemical modification increases the length of the conjugated part of the carotenoid molecule, causing a red shift in its absorbance spectrum [157]. These examples also highlight an emerging pattern: not only are the same genes involved in convergent evolution among populations and species, but so are specific molecular regions [158]. Here, we showed that the *EDNRB2* gene was convergently selected in blue peafowls, chickens, and geese. The 3 types of leucistic plumage birds have different mutation types in the same gene. The reason for the leucistic plumage in chickens is that the protein molecular structure of the *EDNRB2* gene has changed [28]. Although the mutation sites in peafowls and geese are different, they ultimately cause premature termination of codons, which triggers the NMD molecular mechanism of organisms and prevents melanocytes from being transported to plumage [29]. Our results demonstrate that not only the same gene regions are involved in convergent evolution between populations and species, but also specific molecular response mechanisms resulting from mutations in this region are involved. Although this particular case cannot be generalized to other phenotypes, it emphasizes the need to precisely clarify the role of convergent evolution in the fixation of Mendelian phenotypes.

## Additional Files

**Supplementary Fig. S1**. Pipeline of the draft genome assembly and genome annotation of blue peafowl (WP-1).

**Supplementary Fig. S2**. Estimation of the genome size of the blue peafowl by *k*-mer analysis.

**Supplementary Fig. S3**. Hi-C–based chromosome-level assemblies of blue peafowl genome and green peafowl.

**Supplementary Fig. S4**. BUSCO assesses the completeness of published genomes and this assembled genome.

**Supplementary Fig. S5**. Genome synteny and collinearity among the blue peafowl and turkey.

**Supplementary Fig. S6**. Mitochondrial genome map of blue peafowl.

**Supplementary Fig. S7**. ML phylogenetic tree of 15 birds inferred using single-copy orthologous genes.

**Supplementary Fig. S8**. ML phylogenetic tree and molecular clock dating analysis of 8 birds based on mitochondria genomes.

**Supplementary Fig. S9**. Introgression between peafowl, chicken, and turkey calculated using Dsuite heuristic approach.

**Supplementary Fig. S10**. Population size history inference of blue peafowl (BP) and green peafowl (GP).

**Supplementary Fig. S11**. Group history dynamic simulation of blue peafowl and green peafowl by using DADI.

**Supplementary Fig. S12**. KEGG pathway enrichment analysis of the expansion gene family of peafowl.

**Supplementary Fig. S13**. KEGG pathway enrichment analysis of PSG and RGs of peafowl. KEGG pathways with *P* < 0.05 are shown.

**Supplementary Fig. S14**. Variation annotation information of all peafowl individuals.

**Supplementary Fig. S15**. Group structure of blue peafowl and green peafowl.

**Supplementary Fig. S16**. Nucleotide diversity of blue peafowl and green peafowl.

**Supplementary Fig. S17**. Linkage disequilibrium in peafowl groups with individuals greater than 2.

**Supplementary Fig. S18**. Runs of homozygosity (ROH) in peafowl groups with individuals greater than 2.

**Supplementary Fig. S19**. Each chromosome distribution of shared allele SNPs between blue peafowl (blue) and green peafowl (green) in hybrid green peafowl individuals.

**Supplementary Fig. S20**. Comparison of hybrid individual green peafowl and purebred green peafowl.

**Supplementary Fig. S21**. Manhattan plot of BPB blue peafowl group introgression with a window of 2,500 SNPs and a step size of 500 SNPs (P1, P2, P3, O).

**Supplementary Fig. S22**. Phylogenetic tree of 77 peafowl individuals constructed in *IL6, IGF2BP3, TGBR1, ISPD, MEOX2, GLI3*, and *MC4R* gene regions using the ML method.

**Supplementary Fig. S23**. Enrichment analysis of green peafowl introgression regions in the BPB blue peafowl group.

**Supplementary Fig. S24**. Caused mutation of the *EDNRB2* gene locus (chr4: g.12583552 G>A) for the leucistic plumage trait in white peafowls.

**Supplementary Fig. S25**. Bar plot of differential mRNA expression (log_2_-transformed fold change) and (transcripts per million [TPM]) of pigmentation-related genes expressed in the plumage of white peafowls (*n* = 6) versus blue peafowls (*n* = 6).

**Supplementary Fig. S26**. Bar plot of mRNA expression (transcripts per million [TPM]) of the white plumage follicle tissue and blue plumage follicle tissue of the same pied peafowl.

**Supplementary Table S1**. Statistics of genome assembly data of blue peafowl (WP-1).

**Supplementary Table S2**. Summary of *de novo* genome assembly of blue peafowl.

**Supplementary Table S3**. Assembly assessment of completeness using BUSCOs.

**Supplementary Table S4**. Statistics of repeats in our assembled genome.

**Supplementary Table S5**. Statistics of noncoding RNAs in the assembly of peafowl.

**Supplementary Table S6**. Four models simulate group history dynamic of blue peafowl and green peafowl by using DADI.

**Supplementary Table S7**. PGS (positive selection gene) of peafowl by using the branch-site models.

**Supplementary Table S8**. PSG (positive selection gene) and RGs (rapidly evolving genes) of green peafowl by using the branch-site models and branch models.

**Supplementary Table S9**. PSG (positive selection gene) and RGs (rapidly evolving genes) of blue peafowl by using the branch-site models and branch models.

**Supplementary Table S10**. All sample information.

**Supplementary Table S11**. Sequencing depth and coverage of all samples.

**Supplementary Table S12**. *f*3 statistics of peafowl groups.

**Supplementary Table S13**. “Core” group individuals of green peafowl and blue peafowl by using *f*3 statistics and ADMIXTURE.

**Supplementary Table S14**. D statistics of introgression regions (BPW, BPB, GYN, chicken).

**Supplementary Table S15**. *Fst* value of blue peafowl and white peafowl.

**Supplementary Table S16**. All variations within 10 kb before and after the SNP (chr4:g.12583552 G>A). No CNV was found in the interval.

**Supplementary Table S17**. Genotype distribution of the short deletion at chromosome 4 (chr4: g.12583552 G>A) in blue peafowl and white peafowl.

giae124_Supplemental_Files

giae124_GIGA-D-24-00290_Original_Submission

giae124_GIGA-D-24-00290_Revision_1

giae124_Response_to_Reviewer_Comments_Original_Submission

giae124_Reviewer_1_Report_Original_Submission huirong Mao -- 9/3/2024

giae124_Reviewer_2_Report_Original_Submission Subhradip Karmakar, PhD -- 9/16/2024

## Abbreviations

BIC: Bayesian information criterion; BQSR: Base Quality Score Recalibrator; BUSCO: Benchmarking Universal Single-Copy Orthologs; CCS: Circular Consensus Sequencing; CI: confidence interval; DADI: diffusion approximations for demographic inference; DAPI: 4′,6-diamidino-2-phenylindole; DEG: differential expression gene; FISH: fluorescence in situ hybridization; GIM: Godambe information matrix; GO: Gene Ontology; H&E: hematoxylin and eosin; HMM: hidden Markov model; IGFBP: insulin-like growth factor-binding protein; ILS: incomplete lineage sorting; IUCN: International Union for Conservation of Nature; KEGG: Kyoto Encyclopedia of Genes and Genomes; LGM: last glacial maximum; LINE: long interspersed nuclear element; LTR: long terminal repeat; MHC: major histocompatibility complex; ML: maximum likelihood; Mya: million years ago; NCBI: National Center for Biotechnology Information; ND: Newcastle disease; NG: Naynayxungla glaciation; NGS: next-generation sequencing; NJ: neighbor-joining; PBS: phosphate-buffered saline; PCA: principal component analysis; PSG: positively selected gene; PSMC: pairwise sequentially Markovian coalescent; QV: quality value; RG: rapidly evolving gene; RNA-seq: RNA sequencing; ROH: run of homozygosity; rRNA: ribosomal RNA; SINE: short interspersed nuclear element; SNP: single nucleotide polymorphism; snRNA: small nuclear RNA; TMRCA: time to the most recent common ancestor; tRNA: transfer RNA.

## Data Availability

The datasets presented in this study can be found in online repositories. The genome assembly and corresponding sequencing data were deposited in NCBI with BioProject accession number PRJNA1143721 and CNGBdb with accession number CNP0004612. The whole sequencing data and green peafowl Hi-C data collected were from BioProject accession numbers CNP0002498, PRJNA665082, PRJNA340135, and PRJNA644939. RNA sequencing data collected were from BioProject accession numbers PRJNA661158 and PRJNA271731. The codes for reproducing the results are also provided in the GitHub repository [159]. All additional supporting data are available in the *GigaScience* repository, GigaDB [160].
