# Supplementary material for: Genomic evidence for hybridization and introgression between blue peafowl and endangered green peafowl and molecular foundation of leucistic plumage of blue peafowl
Source: Gigascience. 2025 Feb 19;14:giae124. doi: 10.1093/gigascience/giae124 (PMC11835448; doi:10.1093/gigascience/giae124)

GigaScience

Genomic evidence for hybridization and introgression between blue peafowl and endangered green peafowl and molecular foundation of peafowl white plumage

--Manuscript Draft--

**Manuscript Number:**	GIGA-D-24-00290
**Full Title:**	Genomic evidence for hybridization and introgression between blue peafowl and endangered green peafowl and molecular foundation of peafowl white plumage
**Article Type:**	Research
**Funding Information:**	Beijing Agriculture Innovation Consortium (BAIC06-2023) — Prof. Lujiang Qu
**Abstract:**	Introduction<br><br>The blue peafowl (Pavo cristatus) and the green peafowl (Pavo muticus) have garnered significant public affection due to their stunning appearance, although the green peafowl is currently endangered. The causative mutation for the white plumage color of blue peafowl (also called white peafowl) remains unknown.<br><br>Results<br><br>In this study, we generated a chromosome-level reference genome of the blue peafowl with a contig N50 of 30.6 Mb, including the autosomes, Z and W sex chromosomes, as well as a complete mitochondrial DNA sequence. Data from 77 peafowl whole genomes, 76 peafowl mitochondrial genomes and 33 peafowl W chromosome genomes provided the first substantial genetic evidence for recent hybridization between green peafowls and blue peafowls. We found three hybrid green peafowls in zoo samples rather than in wild samples, with blue peafowl genomic content of 16-34%. Maternal genetic analysis showed that two of the hybrid female green peafowls contained complete blue peafowl mitochondrial genomes and W chromosomes. Some animal protection agencies release captive green peafowls to maintain the wild population of green peafowls. Therefore, to better protect the endangered green peafowl, we suggest that purebred identification must be carried out before releasing green peafowls from zoos into the wild in order to prevent the hybrid green peafowl from contaminating the wild green peafowl. In addition, we also found that there were historical introgression events of green peafowl to blue peafowl in four zoo blue peafowl individuals. The introgressed genomic regions contain IGFBP1 and IGFBP3 genes that could affect blue peafowl body size. Finally, we identified that the nonsense mutation (g.4:12583552G>A) in the EDNRB2 gene is the genetic causative mutation for white plumages color of blue peafowl, preventing melanocytes from being transported into plumages, thereby inhibiting melanin deposition.<br><br>Conclusion<br><br>Our research provides both theoretical and empirical support for the conservation of the endangered green peafowl. The high-quality genome and genomic data also provide valuable resource for blue peafowl genomics-assisted breeding.
**Corresponding Author:**	Lujiang Qu, Ph.D.<br>China Agricultural University<br>Beijing, CHINA
**Corresponding Author Secondary Information:**	
**Corresponding Author's Institution:**	China Agricultural University
**Corresponding Author's Secondary Institution:**	
**First Author:**	Gang Wang
**First Author Secondary Information:**	

Powered by Editorial Manager® and ProduXion Manager® from Aries Systems Corporation

Order of Authors:	Gang Wang
	Xinye Zhang
	Liping Ban
	Xiurong Zhao
	Xufang Ren
	Anqi Chen
	Li Zhang
	Yan Lu
	Zhihua Jiang
	Huie Wang
	Wenting Dai
	Yong Liu
	Zhonghua Ning
	Lujiang Qu, Ph.D.
Order of Authors Secondary Information:	
Additional Information:	
Question	Response
Are you submitting this manuscript to a special series or article collection?	No
**Experimental design and statistics**<br><br>Full details of the experimental design and statistical methods used should be given in the Methods section, as detailed in our Minimum Standards Reporting Checklist. Information essential to interpreting the data presented should be made available in the figure legends.<br><br>Have you included all the information requested in your manuscript?	Yes
**Resources**<br><br>A description of all resources used, including antibodies, cell lines, animals and software tools, with enough information to allow them to be uniquely identified, should be included in the Methods section. Authors are strongly encouraged to cite Research Resource Identifiers (RRIDs) for antibodies, model organisms and tools, where possible.	Yes

Powered by Editorial Manager® and ProduXion Manager® from Aries Systems Corporation

Have you included the information requested as detailed in our Minimum Standards Reporting Checklist?	
**Availability of data and materials**<br><br>All datasets and code on which the conclusions of the paper rely must be either included in your submission or deposited in publicly available repositories (where available and ethically appropriate), referencing such data using a unique identifier in the references and in the “Availability of Data and Materials” section of your manuscript.<br><br>Have you have met the above requirement as detailed in our Minimum Standards Reporting Checklist?	Yes

Powered by Editorial Manager® and ProduXion Manager® from Aries Systems Corporation

Manuscript
Click here to access/download;Manuscript;Manuscript-nocite.docx

Genomic evidence for hybridization and introgression between blue peafowl and endangered green peafowl and molecular foundation of peafowl white plumage

GangWang[1¶], Xinye Zhang[1], Liping Ban[1], Xiurong Zhao[1], Xufang Ren[1], Anqi Chen[1], Li Zhang[4], Yan Lu[3], Zhihua Jiang[5], Xiaoyu Zhao[8], Junhui Wen[9], Yalan Zhang[1], Xue Cheng[1], Huie Wang[6], Wenting Dai[1], Yong Liu[7], Zhonghua Ning[1], Lujiang Qu[1*]

[1] College of Animal Science and Technology, China Agricultural University, Beijing, China

[2] College of Grassland Science and Technology, China Agricultural University, Beijing, China

[3] Beijing Key Laboratory of Captive Wildlife Technologies, Beijing Zoo, Beijing, China

[4] Institute of Animal Husbandry and Veterinary Medicine, Beijing Academy of Agricultural and Forestry Sciences, Beijing, China Beijing

[5] Department of Animal Sciences, Washington State University, Pullman, USA

[6] School of Animal Science and technology, Tarim University, Xinjiang, China

[7] Nongxiao Breeding Poultry Breeding Co., Ltd. Beijing, China

[8] Xingrui Technology Co., Ltd. Hebei, China

[9] Institute of Animal Husbandry and Veterinary Medicine, Beijing Academy of Agricultural and Forestry Sciences, Beijing, China

*Correspondence address. Lujiang Qu, College of Animal Science and Technology, China Agricultural University, Beijing, China. E-mail: quluj@163.com.

Abstract

Introduction: The blue peafowl (*Pavo cristatus*) and the green peafowl (*Pavo muticus*) have garnered significant public affection due to their stunning appearance, although the green peafowl is currently endangered. The causative mutation for the white plumage color of blue peafowl (also called white peafowl) remains unknown.

Results: In this study, we generated a chromosome-level reference genome of the blue peafowl with a contig N50 of 30.6 Mb, including the autosomes, Z and W sex chromosomes, as well as a complete mitochondrial DNA sequence. Data from 77 peafowl whole genomes, 76 peafowl mitochondrial genomes and 33 peafowl W chromosome genomes provided the first substantial genetic evidence for recent hybridization between green peafowls and blue peafowls. We found three hybrid green peafowls in zoo samples rather than in wild samples, with blue peafowl genomic content of 16-34%. Maternal genetic analysis showed that two of the hybrid female green peafowls contained complete blue peafowl mitochondrial genomes and W chromosomes. Some animal protection agencies release captive green peafowls to maintain the wild population of green peafowls. Therefore, to better protect the endangered green peafowl, we suggest that purebred identification must be carried out before releasing green peafowls from zoos into the wild in order to prevent the hybrid green peafowl from contaminating the wild green peafowl. In addition, we also found that there were historical introgression events of green peafowl to blue peafowl in four zoo blue peafowl individuals. The introgressed genomic regions contain *IGFBP1* and *IGFBP3* genes that could affect blue peafowl body size. Finally, we identified that the nonsense mutation (g.4:12583552G>A) in the *EDNRB2* gene is the genetic causative mutation for white plumages color of blue peafowl,

preventing melanocytes from being transported into plumages, thereby inhibiting melanin deposition.

Conclusion: Our research provides both theoretical and empirical support for the conservation of the endangered green peafowl. The high-quality genome and genomic data also provide valuable resource for blue peafowl genomics-assisted breeding.

Keywords: Peafowl, Hybridization, Introgression, Conservation, White plumage

Introduction

The peafowl (Aves, Galliformes, Phasianidae, *Pavo*), which includes the blue peafowl, *Pavo cristatus*, and green peafowl, *Pavo muticus*, are widely regarded as symbols of beauty, nobility, auspiciousness, and good luck in Asian culture [1, 2]. They are extensively used in art, religion, literature, and decoration [3]. Their irreplaceable symbolic significance has led to extensive research in the fields of ecology [4], archaeology, history [5] and genomics [6, 7]. Some studies have found the phylogenetic positions of the peafowl in Phasianidae, but the phylogenetic relationship of the peafowl to the chicken and turkey in the Phasianidae family is strongly controversial [5, 6, 8].

The blue peafowl is distributed in several South Asian countries and widely bred all over the world. It was designated as National Bird of India in 1963 and granted the utmost protection [9]. The green peafowl is larger in size than the blue peafowl [3, 10]. Unfortunately, as the only native peafowl in China, the green peafowl is classified as Endangered on the International Union for Conservation of Nature (IUCN) Red List and categorized as Critically Endangered on China’s Biodiversity Red List [10-12]. It was once widely and commonly distributed over subtropical and tropical forests in East and Southeast Asia [13, 14]. However, the green peafowl suffered a sharp

population decline over the past three decades and now the number of wild green peafowls is less than 500 in scattered habitats [15].

The endangered situation of the green peafowl is mainly attributed to habitat fragmentation due to climate change and agricultural activate, illegal poaching, etc. [11, 12, 14, 16]. However, a critical issue has been overlooked. The blue peafowl and green peafowl are closely related species, and although their wild habitats do not overlap, hybridization between them may still occur due to human breeding or other unknown factors [8, 17, 18]. The hybrid green peafowls are morphologically difficult to distinguish from purebred green peafowls. Hybridization with closely related species mixes gene pools and potentially loses genotypically distinct populations. These phenomena can be especially problematic for endangered species contacting more abundant ones and could contribute to the extinction of endangered species [19-21], which has been demonstrated in red wolf, plains bison and endangered Java warty pig [22-25].

Introgression is the transfer of genetic material from one species into the gene pool of the other by the repeated backcrossing of an interspecific hybrid with one of its parent species [26]. Introgression is a long-term process, and it plays an extremely important role in species traits and environmental adaptation [27]. A typical finding of introgression is the historical introgression of *Homo sapiens* by Denisovans and Neanderthals to improve the immunity of Homo sapiens [28]. At present, also due to the lack of genomic data and related studies, we do not know whether there is introgression between blue peafowl and green peafowl and whether introgression has an effect on peafowl traits.

White peafowls are blue peafowl plumage colour mutants, which is characterized by the plumages are white but the eyes are black because they contain melanin [1]. Studies have confirmed

that the white plumage trait of blue peafowls conforms to the Mendelian law of autosomal segregation [29]. In chickens and geese, this white plumage mutant individual, caused by leucism rather than albinism, is explained by mutations in multiple genes involved in the differentiation and migration pathway of melanocytes [30, 31].

In this study, we firstly constructed the first chromosome-level genome of a blue peafowl including autosomes, sex chromosomes and mitochondrial DNA by using PacBio HiFi CCS and Hi-C sequencing. These genomic resources were utilized to analyze the position of the peafowl in the time-calibrated phylogenetic tree and determine the divergence time between blue peafowl and green peafowl. We also collected and reported datasets of genomic variation in wild and zoo green peafowls and blue peafowls. We aimed to systematically delineate the structure of Asian peafowl genetic diversity, and to detect recent hybridization and historical introgression events between blue peafowl and green peafowl. In addition, we investigated the genetic basis of the white plumage phenotype in peafowls and seeked to ascertain whether the same gene determining white feather color in peafowls are the same as those in other birds, and what are the similarities and differences in the molecular mechanisms of the same phenotype. Our research not only provide evidences for understanding the evolution of peafowls but new insights into saving the endangered green peafowl.

Materials and Methods

Sample collection

In this study, we collected one female blue peafowl (*Pavo.cristatus*) from China Beijing Zoo for HiFi and Hi-C sequencing. We also collected two green peafowls (*Pavo.muticus*) from China

Beijing Zoo, 12 blue peafowls from Hebei XingRui CO.LTD and the China Beijing Zoo, and 6 white peafowls (*Pavo.cristatus*) from Beijing Agricultural Vocational College for whole genome resequencing, while all resequenced peafowl individuals are artificially bred individuals. In addition, we collected samples of 54 green peafowls and 3 blue peafowls from NCBI databases and CNSA databases (https://db.cngb.org/cnsa/). Among all the peafowl samples from the database, 13 green peafowls were artificially bred individuals, 44 green peafowls were wild individuals, and all blue peafowls were artificially bred individuals. We obtained the feather follicle tissues of 6 blue peafowls and 6 white peafowls from the peafowl breeding garden in Mentougou District, Beijing for transcriptome sequencing, and combined them with the transcriptome data of 20 blue peafowls published on NCBI for subsequent genome annotation and Transcriptome analysis. In addition, in order to conduct population verification of genetic mutation of plumage color, we obtained blood samples from 11 white peafowls, 29 blue peafowls and 1 pied peafowl (blue white-flight plumage color) from Beijing Agricultural Vocational College and the Peafowl Breeding Park in Mentougou District, Beijing. The full sample information can be found in the Table S1. All of the animals in this study were reviewed and approved by Ministry of Agriculture of China (Beijing, China), Animal Welfare Committee of China Agricultural University (Beijing, China).

Blue peafowl HiFi sequencing and Hi-C sequencing

The genomic DNA extractions were performed on blood from a single female blue peafowl (*P.cristatus*) individual, using the DNAeasy Blood & Tissue Kit (QIAGEN, Valencia, CA) following the manufacturer’s instructions. The DNA was quantified using the NanoDrop ND-2000 Spectrophotometer (Thermo Fisher Scientific, Waltham, MA) with its standard protocol. The

extracted DNA was used to construct PacBio SMRTbell TM library prepared with the Sequel Sequencing Kit 3.0, according to the released protocol from the PacBio Company. The library was processed for PacBio HiFi CCS sequencing (https://github.com/PacificBiosciences/ccs) on the PacBio SequelIImachine by BGI Technologies company (GuangDong, China). A total of 4,085,715,549 bp CCS data was generated. Average CCS length was over 14,503 bp and the longest CCS read length achieved 44,082 bp.

For Hi-C sequencing, the blood was fixed using formaldehyde for 15 min at a concentration of 1%. The chromatin was cross-linked and digested using the restriction enzyme *HindIII*, then blunt end-repaired, and tagged with biotin. The DNA was religated with the T4 DNA ligation enzyme. After ligation, formaldehyde crosslinks were reversed and the DNA purified from proteins. Biotin-containing DNA fragments were captured and used for the construction of the Hi-C library. The Hi-C library was sequenced on an Illumina HiSeq X Ten platform, producing ~101.15 Gbp Clean Data.

Genome assembly and Hi-C scaffolding

Jellyfish (v2.3.0) was used to obtain a frequency distribution of k-mer counting with the clean reads, producing kmer frequency distributions of 31-mers [32]. Then, GenomeScope 2.0 was used to evaluate the peafowl genome size [33]. We used hifiasm to assemble the long reads into contigs by using default parameters [34]. Reads shorter than 8000 bp were discarded. Then, the yahs pipeline was used to join the contigs into chromosomes [35, 36]. The Hi-C contact map based on the draft chromosomal assembly was then visualized in Juicebox which also allowed for manual adjustment of the orientations and order of contigs along the chromosomes (Fig S1a). Although the newly released genome of the green peafowl is relatively complete, it is poorly collinear with the

chicken genome due to imperfect Hi-C scaffolding [7]. We downloaded the Hi-C sequencing data corresponding to the published green peafowl genome and used yash to improve the chromosome-level genome of green peafowl. The consistency and integrity of the assembled blue peafowl genome (WP-1) were separately assessed using BUSCO, based on single-copy orthologues from the AVES (odb10) database. And the Merqury (v.1.3) was also used to evaluate assembly. [37] To obtain the white peafowl mitochondrial genome, we assembled de novo using the NOVOPlasty (v.4.3.1) with default parameters [38]. The mitochondrial sequence length is 16,694bp (Fig S6).

Blue peafowl genome annotation

To annotate the repeat content, we first used RepeatModeler2 to predict and classify TEs throughout the genome [39]. The newly predicted families of TEs and tandem repeats were then combined with the Repbase library (http://www.repeatmasker.org/) (RepBase17.01) to annotate repeats using RepeatMasker (v4.0.7) (http://repeatmasker.org). In addition, we used LTR_finder to identify long terminal repeat (LTR) sequences [40].

To predict mRNA-encoding genes in the blue peafowl genome, we performed Ab initio gene prediction, transcriptome-based gene prediction and homology-based predictions. For the homology-based predictions, we used protein data from six species (*Homo sapiens*, *Mus musculus*, *Pavo muticus*, *Meleagris gallopavo*, *Gallus gallus*, *Oxyura jamaicensis*) that were retrieved from the Ensembl (release 64) database and identified candidate coding regions with miniport (v.0.13) [41]. For the transcriptome based gene prediction, We mapped the collected RNA-seq reads using HISAT2 (v.2.1.0) [42], and assembled the transcriptomes using StringTie [43]. TransDecoder (https://github.com/TransDecoder/TransDecoder) was used for identifying candidate coding regions

within transcript sequences. To make de novo predictions, BRAKER2 (v.2.1.6) was run to use the homology protein as hints to generate predicted gene models from AUGUSTUS (v.3.4.0) and to train the hidden Markov model (HMM) of GeneMark-ET (v.3.67_lic) [44-46]. EVidenceModeler software was used to integrate the gene set and generate a non-redundant and more complete gene set via integration of the three respective annotation files that were assigned different weights (ab initio prediction was "1", homology-based prediction was "5", and transcriptome-based prediction was "10"). [47] Finally, PASA was used to correct the annotation results of EVidenceModeler for the final gene set [48]. Functional annotation of the protein-coding genes was accomplished using eggNOG-Mapper (v.2) [49], a tool that enables rapid functional annotations of novel sequences on the basis of pre-computed orthology assignments, against the EggNOG (v.5.0) database. And the protein database of SwissProt, NR, Pfam were also used to annotate Gene function [50-52]. We also mapped the reference genes to the KEGG pathway database and identified the best match for each gene [53]. tRNAscan-SE (v.2.0.11) software was used to search for the transfer RNA (tRNA) sequence of genome [54], with INFERNAL (v.1.1.3) from Rfam to predict microRNAs and snRNA of genome [55]. And barrnap (v.0.9) (https://github.com/tseemann/barrnap) was used to annotated ribosomal RNA (rRNA) sequence of genome (Fig S1b).

For mitochondrial genome annotation, we submitted it to the AGORA web tool [56], with the protein-coding and rRNA genes of the Gallus gallus mitochondrial genome (accession number: NC_053523.1) as a reference.

Genome synteny and collinearity analysis

We used the MUMmer (v.4.0.0rc1) tool nucmer to perform the pairwise whole genome

alignment with the parameter "-b 400" [57]. The alignments were filtered to keep the oneto-one best hits using delta-filt from the MUMmer package. Unanchored scaffolds were excluded from the alignments. We also performed collinearity analysis using MCscanx with default parameters [58]. The NGenomeSyn (v.1.4.1) was used for synteny visualization [59].

Phylogenetic tree and divergence time

The protein sequences of 15 species were used to search the orthologues using OrthoFinder2 [60]. The results showed that a total of 17,999 orthogroups were identified in 13 species, of which 10,323 single-copy orthologues were shared among these species. These single-copy orthologues were subsequently converted into coding sequence alignment by tracing the coding relationship using pal2nal.v14 [61]. Then, we construct a phylogenetic tree using RAxML (v.8.0.0) with commands: "raxmlHPCAVX -N 100 -b 15738 -f j -m GTR+G -s sample.phy -n all.tree" [62]. Additionally, the divergence time of all species was estimated and calibrated through the divergence time between *Gallus Gallus* and *Cygus olor*, *Tympanuchus pallidicinctus* and *Centrocercus urophasianus*, and *Bambusicola Thoracicus* and *Gallus Gallus* from the TimeTree database [63]. We then used BASEML to estimate the overall mutation rate with the time calibration on the root node (86.34 MY). General reversible substitution model and discrete gamma rates were estimated by the maximum likelihood approach under strict clock. The divergence time was then estimated using MCMCtree [64]. In addition, we also collected the mitochondrial sequences of 8 species from the NCBI, combined with our assembled mitochondrial sequences, constructed a ultrametric tree of maternal inheritance.

We aligned the genomes of chickens, turkeys and two species of peafowls, with swans as the

outgroup to form a five-species whole genome alignment data by using cactus (v.2.6) with default parameters [65]. For the phylogeny of local alignments of sliding windows among all compared genomes, we partitioned the data into nonoverlapping sliding windows with varying window sizes of 100kb to reconstruct phylogenetic trees. All windows with more than 75% gaps were removed. RAxML was used to construct maximum likelihood trees from each alignment, and the low consensus trees (bootstrap < 50%) were discarded [62].

Estimates of the effective population size and divergence time

To characterize the historical demography of the wild green peafowl and blue peafowl, a strategy of combination of two complementary algorithms was employed for cross-validation and comparison. In general, the popular individual-genome-based PSMC approach was used to retrieve distant past history, while the SMC++ method based on various algorithms and data exploration were employed to better characterize more recent demography. For all of these analyses, the mutation rate was set to $1.33 * 10^{-9}$ per site per year estimated for the Indian peafowl and the generation time assumed to be four years to scale demographic events in calendar times, following a study on the blue peafowl [5, 66]. The PSMC analysis [67], which utilized LD information, was conducted on autosomes of the high-coverage de novo assembly. We used bcftools to generate autosomal fasta files, respectively, according to recommendation in the PSMC documentation. The PSMC 100 bootstraps were performed with parameters optimized for birds (N30 –t5 –r5 –p 4+30*2+4+6+10) to determine variance in *Ne* estimates [68]. And we plotted the PSMC by using python script (https://github.com/shengxinzhuan/easy_psmc_plot). A recently published approach with higher resolution in the recent past compared to PSMC accuracy, SMC++ [69], was used to

predict the demographic history (or population sizes and divergence times) of green peafowl and blue peafowl based on multiple unphased individuals. The short generation time of wild green peafowl and blue peafowl makes possible the reliable and precise estimation of effective population sizes in the recent past using the method of SMC++ (v.1.15.2) [70].

We also used DADI to infer the divergence time between wild green peafowl and blue peafowl [71, 72]. We simulated four models with the same dataset under the two-population model in DADI independently. Model 1 (sym_mig): Instantanous size change followed by exponential growth with no population split; Model 2 (bottlegrowth_2d): Instantanous size change followed by exponential growth then split with migration; Model 3 (bottlegrowth_split_mig): Split into two populations of specifed size, with migration; Model 4 (split_asym_mig): Split into two populations of specifed size, with asymetric migration. To avoid the effect of selection on the demographic analysis, we focused on non-coding regions and extracted non-coding SNPs. To avoid the linkage of SNPs, we thinned the non-coding SNPs to 1% and obtained a dataset of 135,873 SNPs [73]. Owing to the unknown ancestral state of each SNP, we folded the frequency spectrum. The projection value was selected based on the strategy of maximizing the number of segregated sites. The starting parameter values for the first round were randomly assigned, and the best parameters obtained after completion of a round were used as the starting parameters for the subsequent rounds. After the convergence of the parameters, we retained the model with the highest log-likelihood as the final simulation result. The parameters of the optimal model were converted into absolute units using the average mutation rate per generation and generation interval. Confidence intervals for the parameters were generated using the Godambe information matrix (GIM) with 100 bootstraps [74].

All blue peafowl and green peafowl populations used for SMC++ and DADI analyzes excluded

hybrid and introgressed individuals found in subsequent studies.

Gene family construction and Branch-specific positive selection

To define gene families, we used coding sequences of all 15 species and extracted the longest protein for each gene. Gene family size expansion and contraction analysis was performed by CAFE5 (v.5.0.0) [75], and the results from OrthoFinder2 and a phylogenetic tree with divergence times were used as inputs for CAFE. These expanded gene families were annotated and classified through the analysis of GO ontology and KEGG pathways to further explore the impact of adaptive evolution on peafowl by using Kaobas (v.3.0) [76].

We obtained codon alignments for the single-copy orthologous groups by aligning Coding sequence by using MAFFT. Nonsynonymous and synonymous substitution rates (Ka/Ks) were calculated using codeml program in PAML (v.4.5) package [64]. We used the branch site model and two-ratio models to detect signatures of natural selection on coding genes of peafowl. Statistical significance was determined using likelihood ratio tests. Functional annotation of the obtained gene dataset was also performed using Kobas (v.3.0) [76].

Whole-genome resequencing and variant calling

We cleaned the Illumina NGS raw data to remove adaptors, trim low-quality bases and remove "N" sites with fastp (v.0.20.0) [77]. Subsequently, clean reads were mapped to our blue peafowl genome using BWA (v.0.7.10-r789) with default parameters [78]. High-quality mapped reads (mapped, nonduplicated reads with mapping quality ≥ 20) were selected with SAMTools (v.1.3.1)

and the following commands: “-view -F 4 -q 20” and “rmdup”73 [79]. For all samples, we used the "bamqc" module in Qualimap (v.2.2.1) to perform sequencing depth statistics [80]. Only high-quality mapped reads were used for variant calling with GATK (v.4.2.6.1) [81]. BAM files were sorted and marked as PCR duplications with Picard (v.2.27.5, http://broadinstitute.github.io/picard/). There is no well-annotated SNP and short-indel database for blue peafowl, so it was not feasible to use the “Base Quality Score Recalibrator” (BQSR) and “IndelRealigner” options of GATK. To carry out variant calling, we used the command “HaplotypeCaller”, which calls SNPs and indels simultaneously via local de novo assembly of haplotypes in an active region. Applying the “hard filtering” method, we obtained an initial set of high-confidence SNPs and indels. The parameters of “hard filtering” were set by default, i.e., for SNPs we used QD < 2.0, FS > 200.0, SOR > 10.0, MQRankSum < −12.5, and ReadPosRankSum < −8.0, while for short indels, we considered QD < 2.0, FS > 200.0, and ReadPosRankSum < −8.0. After the initial filtering step, the number of SNPs and short Indels became 16,330,028 and 2,153,530, respectively. Notably, the ratios of Ts/Tv to 2.534 with filtering of the raw SNPs, which showcases the high quality of the SNP call sets. For many downstream analyses, the core set of SNPs and Indels were acquired by setting the MAF cut-off at 0.05. We performed SNP and Indel annotation according to the blue peafowl genome using the SnpEff (v.5.1) [82]. The intergenic region of the genome encompasses about 45.73% of SNPs and 70.0% of Indels. About 1.573% of SNPs are located in the coding sequence, and the nonsynonymous to synonymous SNP ratio is 0.508%. In comparison, only 1.081% of Indels are found in the coding sequence. In addition, we also used CNVcaller to detect copy number variations in *P.cristatus* for subsequent analysis of the feather color of white peafowl [83].

Population genetic structure

We chose the core set of SNPs (MAF greater than 0.05) for additional pruning. PLINK (v1.90b6.12) was used to remove SNPs having high LD ($r^2 \geqslant 0.5$) within a continuous window of 50 SNPs (step size 5 SNPs) [84], which yielded 3,147,759 SNPs for both analyses. The parameter used in this procedure was “--indep-pair-wise 50 5 0.2”. The results obtained from the above procedure was be used to perform principal component (PCA) analysis by using VCF2PCACluster (https://github.com/hewm2008/MingPCACluster). We used ADMIXTURE software to analyze the population structure of all peafowl samples with kinship (K) set from 2 to 5 [85]. Finally, we constructed the unrooted Neighbor-joining (NJ) tree by MEGA software (v.11) [86]. The FigTree software (v.1.4.4) was used for visualization. We also constructed the maximum likelihood (ML) tree for all individuals using treemix (v.1.13) [87].

Population genetic diversity

As for calculation of the nucleotide diversity and the linkage disequilibrium decay of each peafowl population, we used two softwares, VCFtools and PopLDdecay (v.3.42) with default parameters, respectively [88, 89]. In the study of nucleotide diversity and ROH, we used the same set of SNPs. Since LD analysis and nucleotide diversity analysis require the number of individuals in the peafowl population to be greater than 1, we removed populations with 1 individual and hybrid individual. Runs of homozygosity (ROH) of each individual peafowl were identified using the homozyg option implemented in the PLINK, which slides a window of 50 SNPs (-homozyg-window-snp 50) across the genome estimating homozygosity. The following settings were

performed for ROH identification: (1) required minimum density (-homozyg-density 50); (2) number of heterozygotes allowed in a window (-homozyg-window-het 3); (3) the number of missing calls allowed in window (-homozyg-window-missing 5). The ROH of each peafowl breed were counted and divided into four categories according to the length: 0.5-1Mb, 1-2Mb, 2-4Mb, >4Mb [90].

Phylogenetic analysis of peafowl mitochondria

The BAM alignments were converted to fastq and subsequently used with Mapping Iterative Assembler (v.1.0) (https://github.com/mpieva/mapping-iterative-assembler) to assemble a mtDNA consensus sequence. We aligned our 76 mitochondrial genome sequences to a collection of 1 published *Gallus gallus* mitochondrial genome (accession number: NC_053523.1). The "TIM3+F+G4" model of nucleotide substitution was selected by comparing the Bayesian information criterion (BIC) scores in jModelTest [91]. A phylogenetic tree was then inferred using ML methods. The ML analysis was performed with the program Raxml (v.8.0.0) [62]. and approximate likelihood-ratio tests were performed to establish statistical support of internal branches with the *Gallus gallus* as outgroup. The mitochondrial haplotypes were built by using DnaSP (v.6.12.03).

Hybridization analysis between endangered green peafowls and blue peafowls

We used a chromosome painting approach with ancestry-informative sites to validate the delimitation of ancestry blocks detected by the HMM and to visualize patterns of introgression

across the blue peafowl. This approach provides a lower level of resolution for ancestry block delimitation but with higher power to classify regions as derived from either parental genome. To identify introgressed intervals in Hybrid Individual of Pavo muticus, we used Loter (v.1.0.1) to infer the haplotype fragments of blue peafowl among all autosomes of Pavo muticus genomes [92]. In addition, we identified alleles that were differentially fixed in green peafowls and blue peafowls parental populations and had no missing data using the script get_fixed_site_gts.rb. We thinned SNPs to be a minimum of 1 kb apart and mapped these ancestry-informative sites in the green peafowl samples using the scriptplot_fixed_site_gts.rb.

Introgression analysis

Treemix (v1.13) was used to confirm relationships between our focal populations and to visualize migration events between populations [87]. We first built the maximum likelihood tree (zero migration events) in Treemix and then ran Treemix sequentially with one through ten migration events. We supplied this set of 5,671,362 biallelic SNPs to Treemix, rooted with Gallus gallus, and estimated the covariance matrix between populations using blocks of 500 SNPs. Three samples (qhd-01, qhd_02 and GF-f1A) were excluded from this analysis because ADMIXTURE indicated that they were likely hybrid individual. We calculated the variance explained by each model (zero through ten migration events) using the R script treemixVarianceExplained.R.

To investigate the relationship of the blue peafowl to green peafowl populations, we used AdmixTools (v.7.0.2) for the phylogenetic analysis [93]. We also used three-population test estimates (f3 statistics) to test for admixtures across all peafowl populations. Three-population tests consider population triplets (C; A, B), where C is the test population and A and B are the reference

populations. Significantly negative Z-scores (Z ≤ -3.80, after Bonferroni correction for multiple testing) indicate evidence of test population C containing an admixture of both reference populations of A and B.

The ABBA-BABA test, also known as the D-statistic, was used to infer the existence of gene flow between the populations. This analysis of D-statistic values was performed using Dsuite software [94], which can calculate D-statistics at the genome scale across all combinations of populations with VCF input files. According to the principle of ABBA-BABA test calculation, (P1, P2, P3, O) represent four different groups. And O is chicken (*Gallus gallus*) as the outgroup. Dtrios was used to calculate the D and f4-ratio statistics for all trios of populations in the dataset, with a default value of 20,000 for the Jackknife block size. Then, using the Dinvestigate program to calculate the D-value for windows containing useable-size SNPs, the sliding window consisted of 2500 SNPs and a step of 500 SNPs. The locations of the windows with the top 5% D values were obtained and the genes in these windows were regarded as candidate genes for introgression. Functional annotation of the obtained gene dataset was performed using Kobas (v.3.0) [76].

Histological characterization of the genetic mechanism of plumage color in white peafowls and elucidation of the molecular basis

In order to understand the reason of white peafowl (*P.cristatus*) plumages turning white, we used the peafowl individual resequencing SNP data set and used VCFtools to calculate *Fst* [88]. We calculated the genome-wide distribution of *Fst* between Blue peafowl (*P.cristatus*) and White peafowl (*P.cristatus*), using a sliding-window approach (1kb windows with 200bp increments).

Absolute genetic divergence (Dxy) statistic was calculated to strengthen our results by using custom python scripts [95]. The bedtools software (v.2.30.0) was used to annotate the selection regions.

For RNA-seq data analysis, the paired-end reads were mapped to the peafowl reference genome (WP-1) using the HISAT2 (v2.6.1.0) software after quality control [42] . Transcripts were assembled and quantified with the StringTie (v2.1.1) software [43]. The GFFcompare (v0.12.6) was used to compare the alternative transcripts among individuals [96]. The differential expression analysis was performed using the DESeq2 (v1.4.5) package [97]. Ultimately, we enriched candidate genes by the online website KOBAS (v3.0) [76], so as to grasp the functions of selection genes and Differential Expression Genes (DEGs).

To identify the causative mutation that causes blue peafowl feathers to turn white, we also examined the frequency of all (SNP, Indel and CNV) mutations within 10 kb before and after the candidate SNP.

DNA was extracted from blood samples with the DNAeasy Blood & Tissue Kit (QIAGEN, Valencia, CA). The *EDNRB2* gene SNP mutation (g.4:12583552 G>A) was genotyped by Sanger sequencing on an ABI 3730xl DNA Analyzer (Applied Biosystems, USA) according to the manufacturer's instructions. The primer sequences used were as follows: forward primer 5′-TGAAGAAGTGTAAGTCCCGCTG-3′ and reverse primer 5′-AGGTCTCGGTCCCAGTAGTT-3′.

Plumage samples stored in 4% paraformaldehyde were washed in phosphate buffered saline (PBS), dehydrated with a gradient of alcohol solutions (50% → 70% → 80% → 95% → 100% → 100%),

cleared with xylene and infiltrated with paraffin wax. Samples were embedded in paraffin and

sectioned into 4-micrometer-thick tissue sections. Both the plumage samples sections were stained with hematoxylin and eosin (H&E) and Masson-Fontana. We designed a fluorescent in situ hybridization (Fish) probe using the specific sequence of the CDS region of the EDNRB2 gene. Sections were incubated with 4′,6-diamidino-2-phenylindole (DAPI) to stain the nucleus and were imaged with a fluorescence microscope.

Results

Sequencing, assembly and annotation of the blue peafowl genome

The blue peafowl genome consists of 78 chromosomes, including at least 30 microchromosomes (76 autosomes + ZW sex chromosomes) according to the karyotype analysis [98]. In order to assemble a chromosome-level genome for blue peafowl, we generated 38 Gbp of PacBio Circular Consensus Sequencing (CCS) HiFi reads and 101.15 Gbp of chromatin conformation capture (Hi-C) reads. K-mer-based analyses of the Illumina paired-end sequencing reads (51.77 Gbp, 52.71 × sequencing depth) estimated the size of the nuclear genome to be approximately 982.21 Mb (Fig. S2 and Table. S1).

The initial assembly was 1.13 Gbp, consisting of 389 contigs with a N50 length of 30.6 Mb, indicating a high contiguity of the assembly. Contigs were then concatenated to the chromosome-level assembly by Hi-C reads. We ultimately obtained 36 pairs of autosomes (9 macrochromosomes, 27 microchromosomes) and ZW sex chromosomes with genome size (1.041Gbp) (Fig. S3 and Table. S2). Our genome exhibits a 500-fold and 8-fold improvement, in the scaffold N50 (95 Mb),

compared to those of the previously published peafowl genomes reported by Dhar et al. [6] and Liu et al. [1], respectively.

We next evaluated the quality of the genome assembly using Benchmarking Universal Single-Copy Orthologs (BUSCO), Merqury and Illumina short reads [37, 99]. The complete BUSCO of the blue peafowl genome assembly was 97.1%, indicating a high completeness of the gene space (Fig. S4 and Table. S3). Merqury compares k-mers from the assembly to those found in unassembled HiFi reads to estimate the completeness and accuracy. The completeness and quality value (QV) of the genome were 96.34% and 60.47 (>99.99% accuracy) respectively. These results attest to the high accuracy and completeness of our assembly (Table. 1). According to our results, repetitive sequences accounted for 13.38% of the blue peafowl genome (Table. S4), including 10.44% tandem repeats and 7.35% transposable element proteins. Among tandem repeats, Long Interspersed Nuclear Elements (LINEs) constitute the majority, accounting for 7.41%. Additionally, short interspersed nuclear elements (SINEs) 0.05% and long terminal repeats (LTRs) accounts for 2.99%. We also identified 354 microRNAs, 308 tRNAs, 151 ribosomal RNAs, and 334 small nuclear RNAs (Table. S2 and S5). For annotation, the collected mRNA sequencing (RNA-seq) data were aligned to the reference genome, and 37,401 putative protein-coding gene models were predicted.

Table. 1. Quality metrics for the blue peafowl genome assembly generated in the current work and for other blue peafowl and green peafowl genome assemblies published in previous studies.

	Blue peafowl			Green peafowl	
	This study	Liu et al. (2022)	Dhar et al. (2019)	Zhang. et al. (2022)	Dong. et.al. (2021)

Sequencing technology	PacBio (Hifi), Hi-C	Illumina NovaSeq 6000, PacBio (CLR), 10X Genomics, Chicoga	Illumina HiSeq, ONT	PacBio ,Hi-C	Illumina HiSeq
Level	Chromosome	Scaffold	Scaffold	Chromosome	Scaffold
Total scaffolds	38	726	179332	115	2446
Scaffolds N50 (Mb)	95	11.4	0.19	75.5	2
Contigs N50 (Mb)	30.6	6.18	0.1	25.4	0.161
Longest scaffold length (Mb)	199.9	38.6	2.5	113.2	13.4
Total sequence length (Gbp)	1.041	1.047	1.025	1.049	1.061
Total number of predicted protein-coding genes	37401	19465	23153	14935	15584

Peafowl genome rearrangement

Synteny and genome size have generally remained stable over the more than 100 million years' of modern bird evolution [100-103]. Among the 12% of bird species with documented karyotypes, most have diploid numbers ranging from 76 to 82 [100, 102] found that peafowl has a typical bird karyotype with 2n=78 chromosomes, which is consistent with that of chicken. In this study, we assembled 10 pairs of large chromosomes of 2n=74 in total, which slightly conflicts with the results of the karyotype analysis showing that peafowl have 8 large chromosomes and 30 pairs of

microchromosomes of 78 in total (Fig. 1A). Newly improved green peafowl genome has the same number of chromosomes as the blue peafowl.

To understand the similarities between the common peafowl and chicken at the chromosome level, we compared our assembly with the chicken reference genome (*Gallus gallus* 7.0). Most of the large scaffolds had their counterparts to the macrochromosomes in chicken except scaffolds 6 and 8 (Fig. 1F). Among them, chicken chromosomes 6 and 11 were both aligned to blue peafowl scaffolds 6, whereas chromosomes 8 and 9 added up to scaffolds 8. We also compared the assembly with the turkey reference genome (Turkey 5.0). Correspondingly, the first nine scaffolds have their unique counterparts in the macrochromosomes of turkey (Fig. S5).

In addition to observing fusion and fission of large chromosomes among blue peafowl, green peafowl, chicken, and turkey genomes, we also observed some microchromosomes rearrangements (Fig. 1F). Howerver, due to the GC-richness, specific repeats, high microchromosome mutation rate in birds [98], and lack of cytogenetic supports, determining whether these short scaffolds are bona fide chromosomal rearrangements or simply assembly errors is difficult, and further studies are required in this regard [104].

Phylogenetic Trees Resolving the Divergence Time of blue peafowl and green peafowl

Peafowl is a general name for a type of bird of the order Galliformes and Phasianida, which is a consensus in past studies on both morphology and molecular biology [5]. However, local genomic phylogeny within extant Phasianidae, the phylogenetic relationship of peafowls with turkeys and chickens has always been controversial [5].

We obtained 17,999 gene families and 10,323 single-copy orthologous genes from blue peafowl, green peafowl and other 11 species. We generated a whole-genome species tree with the Anseriformes as outgroups by using 5,649,884 sites from the single-copy orthologous genes sets of 13 species, yielding a species tree with 100% bootstrap support for all nodes (Fig. 1B).

According to our results, the Phasianidae family formed a group, with blue peafowls and green peafowls clustered in the Phasianidae branch (Fig. S7). The phylogenetic tree built with four-fold degenerate sites is consistent with the topology of the phylogenetic tree constructed using single-copy orthologous genes, and dated the common ancestor of peafowls to approximately 48.92 million years ago (Mya). The blue peafowl and green peafowl diverged 4.13 Mya within the range of divergence (1.95–6.53 Mya). This divergence coincided with the transition between the Miocene and Pliocene epochs, a period characterized by the rapid evolution and replacement of older species by new ones. Additionally, the human ancestor Australopithecus appeared around this time, approximately 4.2 Mya [105]. Several species of Phasianidae, including *Centrocercus urophasianus, Tympanuchus pallidicinctus*, *Lagopus muta and Lagopus leucura*, also evolved during this period.

Notably, peafowl were found to be closer to chicken than turkey in the Phasianidae family. And the ML tree constructed from mitochondrial sequences shows the same topology. These findings were consistent with the findings of those reported by Dhar and Kimball et al. [6, 106]. But there are several studies showing that peafowl is closer to turkey than chicken, including phylogenetic trees constructed using single-copy orthologous genes [1].

To investigate the reasons for these controversies, we constructed the topologies of locally aligned fragments across the whole genomes of two peafowls and chickens and turkeys with swan as outgroup. Our results found more than 40% phylogenetic discordance, which was evenly

dispersed along the whole reference genome architecture (Fig. 1C).

As is well known, birds survived the Cretaceous–Paleogene extinction event (K-Pg) and underwent multiple species explosions [107]. These species explosion events resulting in incomplete lineage sorting (ILS) have led to a much greater degree of confusion between branches of bird species compared to mammals [108]. The speciation of peafowls coincided with the period of the pheasant family's major explosion. In addition, gene flow between species may also be responsible for inconsistent species phylogenetic relationships between different data sets [109]. Therefore, we use the f4 admixture ratio (f statistic; closely related to Patterson's D), computing f(A, B ;C,O) for all species that fit the relationship ((A, B), C) in four-fold degenerate sites tree, with the outgroup fixed as swan. Significant gene flow between turkey and the ancestral nodes of peafowl was found (Fig. S9). Therefore, we speculate that the high proportion of confusion in the genome-wide phylogenetic trees of peafowls, chickens and turkey is caused by gene flow and ILS caused by rapid ancestral speciation.

Green peafowl and blue peafowl have different demographic histories

To understand the demographic history in peafowls, we investigated each species with the pairwise sequentially Markovian coalescent (PSMC) approach and SMC++. PSMC model to infer the local time to the most recent common ancestor (TMRCA) as well as to assess changes in the historic effective population size (Ne) by taking into account whole-genome data from single deep-coverage (>25×) individuals. The SMC++ method was used to reconstruct the population history of the "core" groups of green and blue peafowls found in subsequent studies.

In our results, the Ne changes of wild green peafowl exhibited by the two methods are somewhat different during the LGP period. The result of the wild green peafowl PSMC is consistent with the results of Dong et al., which showed early population decline from 800 to 210 ka, followed by a bounce to a peak Ne during early LGP (ca. 70 Ka), and a more marked decrease (seven-fold change) throughout the following part of LGP (ca.70–10 Ka) (Fig. 1D). SMC++ analyses showed that during the LGP period, the effective population size experienced a process of first decreasing (ca.110-30 ka) and then increasing (ca.10-20 ka), and suggested a peak Ne for the green peafowl after the LGP period (ca.10 ka). This may be due to differences in the individuals used in the analysis. Such a fluctuating population history broadly agrees with the two demographic patterns of most other threatened species, plausibly suggesting a common genetic consequence of late Pleistocene climatic oscillations.

However, blue peafowl showed an entirely different population demographic history. Both PSMC and SMC++ population-based demographic analyses congruently suggested that the Ne of the blue peafowl experienced a dramatic expansion from 500 to 1000Ka and peaked around 500Ka before the LGP period, and then declined (with a small-scale recovery during the period). We speculate that the blue peafowl may not have adapted to the severe environmental changes during the last glacial period compared to the green peafowl.

We then used the simulation of diffusion approximations for demographic inference (DADI) to calculate the divergence times between the “core” groups of the blue peafowl and the green peafowl in this study. Among the four models defınded, Model 4 (split_asym_mig) had the highest log-likelihood, indicating the best fit of model to the data (Fig. 1E, S10-S11 and Table. S6). The demographic estimated using model 4 indicated that blue peafowl and the green peafowl diverged

~4.32 Mya with a 95% confidence interval (CI) of ± 31180 years which overlapped with the molecular divergence times of blue peafowl and the green peafowl by MCMCtree. The Ne values of blue peafowl and the green peafowl were 1011 (95%CI ± 10) and 2060 (95%CI ± 22). Model 4 also indicated that nonsymmetric gene flow existed between blue peafowl and the green peafowl after divergencing.

Genetic mechanisms underlying peafowls body size and immune system evolution

Like other birds groups [108], extant Phasianidae species exhibit a large range of body sizes, from *Coturnix japonica* (~14 cm) at one end of the spectrum to peafowls (>2.3 m in some individuals with 1.5 m tail plumages) at the other [7]. Thus, phasianidae body size has experienced significant divergence, particularly for the green peafowls with their substantial enlargement in body size.

We detected 123 positively selected genes (PSG) and 126 rapidly evolving genes (RGs) in the green peafowls (Fig. 1B). Among these genes, *IGFBP4* and *IGF2*, which are related to insulin-like growth factors, may have contributed to the evolution of this trait. Several studies on other vertebrate species corroborate the critical role of *IGF-2* in body growth and adult size determination [110]. *IGFBP4* is IGFs binding partner proteins are known to cause changed body size in mouse and human [111]. Blue peafowls are slightly smaller than green peafowls, but it is still among the largest species in the pheasant family [6]. We detected that *MSTN* has undergone rapid evolution in blue peafowls. *MSTN* is well-known as muscle growth inhibitory gene. Some cattle breeds (Belgian blue cattle) exhibit extremely exaggerated body size and muscle content due to mutations in this gene [112].

In birds, the rate of sequence divergence in immune-related genes is usually higher than in the other genes primarily because of the co-evolution of host–pathogen interactions [113]. We performed enrichment analysis on 555 expanded gene families in the common ancestors of the peafowls and found that eight genes (*IFNW1*, *ACTG1*, *NUP98*, *IFNA3*, *KPNA2*, *ACTB*, *RNASEL*, *NXF1*) were enriched in Influenza A KEGG pathway (gga05164) which related to immunity (Fig. 1B and S12) [114]. In addition, we also found that the interleukin family gene *IL7R* and the *CD8BP* gene involved in the adaptive immune response were positively selected in the common ancestor of the peafowls (Table. S7). Although rapid evolution of immune genes was found in a wide range of birds [108], the number of PSGs and RGs genes involved in the Cytokine-cytokine receptor interaction pathway is highest in in blue peafowls (*NODA*, *IL13RA1*, *BMP8A*, *GDF5*) and green peafowls (*CCR10*, *CD40LG*, *ACVR2B*, *CCR7*) (Fig. S13, S8 and Table. S9). It could suggest a key role of this pathway in the evolution of peafowl immune system. Among these genes, major histocompatibility complex (MHC) genes, interleukin family genes, and NF-KB signaling pathway genes play important roles in immune responses.

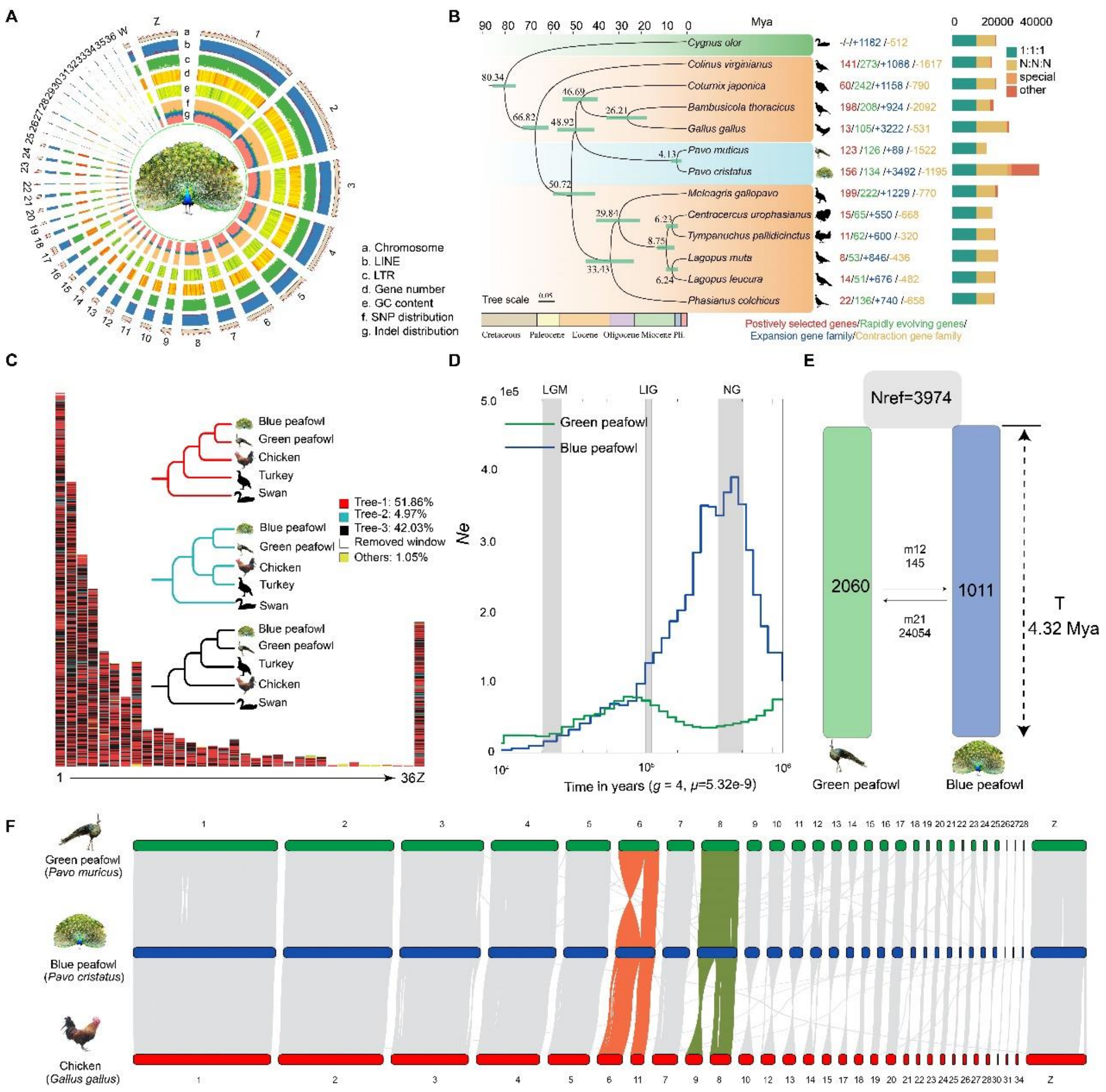

Fig. 1. Assembly, composition, evolution and demographic history of the blue peafowl genome.

(A) Blue peafowl genomic features. (B) Positively selected genes (PGS), rapidly evolving genes (RGs), Gene family, phylogenetic and molecular clock dating analysis of the peafowl genomes with 11 other species, based on single-copy orthogroup data. The grey bars at the internodes represent 95% confidence intervals for divergence times. In the histogram, “1:1:1” indicates that the single-copy orthologs are shared by 13 species with 1 copy. “N:N:N” represents any other orthologous group (missing in 1 species). Species-specific shows the specific orthologs in each species. Other orthologs are unclustered into gene families. (C) Distribution of the genomic phylogenetic

discordance across each chromosome of blue peafowl by 100-kb sliding windows. Different colors on the genome represent the corresponding phylogenetic topology. (D) Population size history inference of blue peafowl and green peafowl respectively, acrossing the last glacial maximum (LGM, approx. 26–19 Ka), the last interglacial period (LIG, approx. 132–112 Ka) and the naynayxungla glaciation (NG, approx. 0.5–0.72 Ma). (E) Best-fit estimates of parameters by using DaDi showed the divergence time of blue peafowl and green peafowl. (F) Genome synteny and collinearity among the blue peafowl and chicken.

Genome resequencing

In this study, 18 blue peafowl and 2 green peafowl different geographical locations in China were selected for genome resequencing (Fig. 2A). For a more comprehensive analysis of peafowls, we also combined our data with available whole-genome resequencing data for 3 blue peafowl individuals and 54 green peafowl individuals from 7 regions in Asia, giving a total of 77 individuals (Table. S10 and S11). Whole-genome resequencing sequences were mapped to our assembled genome (WP-1). After quality control and filtering, 1399.13 Gbp of high-quality sequences were obtained, with an average of 17.48 Gbp per individual. Across all samples, a total of 12,453,511,109 mapped reads was obtained, with an average depth of 18.53 × and an average coverage of 92.07% per individual. After variant calling, a total of 27.78 million variants was obtained, including 16.33 million SNPs and 0.47 million indels. Among all variants, only 1.82% of SNPs and 1.08% of Indels are located in exons. Most mutations are located in intergenic regions (Fig. S14). In addition, we also collected a rooster as an outgroup for subsequent analysis. Of these animals, 76 peafowl individuals were used for a mitochondrial sequence analysis and 38 female peafowl individuals used

for a W-chromosome SNP analysis.

Population genetic structure of peafowl using autosomal variants

Genomic SNPs of blue peafowl and green peafowl were used to analyze the population structure of peafowl, as well as to analyze the introgression between blue peafowl and green peafowl.

The NJ tree of SNPs filtered and trimmed for linkage disequilibrium using chickens as an outgroup demonstrated a clear genetic structure with green peafowl and blue peafowl clustered into each clade (Fig. 3B). Admixture analysis (K from 2 to 9) of the genomic admixture with two population scenarios (K=2) had the best likelihood, and the specimens were divided into two groups corresponding to the morphology-based species identification. The blue peafowl (BPB, BPI, BPJ, BPW, WPB, WPW) formed one cluster, while green peafowl (GKR, GTL, GYN, GYUEN, GZJ) formed the second cluster (Fig. 3C and S15). At K=3, three clusters were observed, some individuals in the green peafowl (GKR, GYUEN, GZJ) have new clusters. The clustering of green peafowl populations shows clear geographical associations with their sampling locations. Notably, the three green peafowl samples (GF-f1A, qhd-01, qhd-02) equally composed of the blue peafowl and green peafowl alleles were observed from k = 2 to 9. PCA analysis also recapitulated these findings. In the first principal component, the blue peafowl and green peafowl clusters were divided along the two eigenvectors without overlap. Three green peafowl individuals (GF-f1A, qhd-01, qhd-02) were positioned outside the discovered clusters, which deviated towards green peafowl in the first principal component (Fig. 3D).

Based on species and geographic location, we performed genetic diversity analysis inferred

nucleotide diversity, the linkage disequilibrium (LD) parameters (r2) and long runs of homozygosity (ROH) for populations with sample sizes >2 (including GYUEN, GZJ, GYN, GTL, GQHD, WPB, BPW, BPB). Among the green peafowl, the GQHD population had the highest genetic diversity, while the GYUEN population had the lowest genetic diversity. BPB populations have the highest genetic diversity and WPB populations the lowest in blue peafowl. We also found that the nucleotide diversity of the green peafowl (π=1.47^{-3}) was lower than that of the blue peafowl (π=1.99^{-3}) (Fig. S16). The LD decay rate of the blue peafowl was slower than green peafowl, the Yunnan green peafowl showed the fastest LD decay and the smallest r2, indicating that they had higher diversity in wild environment (Fig. S17). The length of the long homozygous segment can reflect the degree of inbreeding of the population. Compared with the peafowl, the ROH length of all peafowl populations is short and the number is small. These results suggests that the degree of inbreeding of the green peafowl is much lower than that of the blue peafowl, which may be due to the long-term wild environment of the green peafowl (Fig. S18).

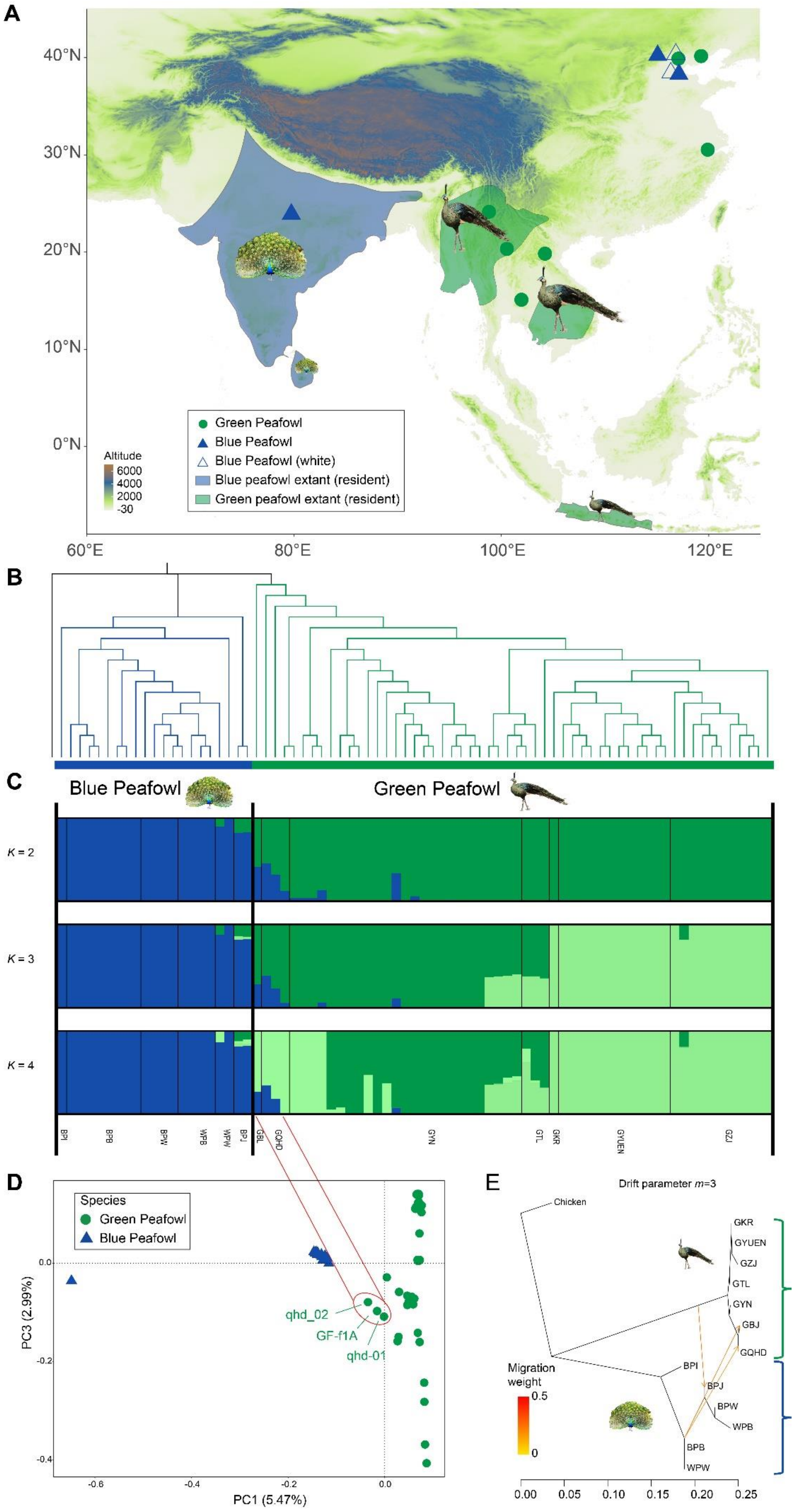
A
40°N
30°N
20°N
10°N
0°N
60°E
80°E
100°E
120°E
Altitude
6000
4000
2000
-30
Green Peafowl
Blue Peafowl
Blue Peafowl (white)
Blue peafowl extant (resident)
Green peafowl extant (resident)
B
C
Blue Peafowl
Green Peafowl
K = 2
K = 3
K = 4
BPI
BPB
BPW
WPB
WPW
BPJ
GBJ
GQHD
GYN
GTL
GKR
GYUEN
GZJ
D
Species
Green Peafowl
Blue Peafowl
PC3 (2.99%)
PC1 (5.47%)
qhd_02
GF-f1A
qhd-01
E
Drift parameter m=3
Chicken
GKR
GYUEN
GZJ
GTL
GYN
GBJ
GQHD
BPI
BPJ
BPW
WPB
BPB
WPW
Migration weight
0.5
0
0.00
0.05
0.10
0.15
0.20
0.25

Fig. 2. Population genetic structure of blue peafowls and green peafowls. (A) Location of the samples used for this study. A total of 77 peafowls, including blue peafowl (n = 22, *P.cristatus*) and green peafowl (n = 55, *P. muticus*), were used. (B) A NJ tree of all peafowls estimated based on high-quality autosomal SNPs. (C) Population genetic structure of peafowl. The length of each colored segment represents the proportion of the individual genome from the ancestral populations (K = 2–4), population names are at the bottom. (D) PCA plot of peafowl individuals. The three individuals in the red circle are the green peafowl that may have hybridized with the blue peafowl. (E) Genetic migration inferred using TreeMix with best draft parameter (m = 3), while the migration weight (proportion of admixed population received from source) is indicated on the arrow by the number and color.

Maternal phylogenetic analyses of peafowl

Both W-chromosome and mitochondrial DNA (mtDNA) haplotypes represent strong foci in the investigations of Aves. Here, we used complete mitogenomes to construct a haplotype network and rooted haplotype trees with chicken as outgroup (Fig. 3C and 3D). A total of 12 mitochondrial haplogroups (G1, G1a, G2, G2a, G2b, G3, G3a, G3b, G3c, G4 for green peafowl, and B1, B2 for blue peafowl) emerged. We also used 745 SNPs in the W-chromosome to construct a phylogenetic tree (Fig. 3E). The evolutionary tree can clearly divide the blue peafowl and green peafowl into two branches.

There are two green peafowl individuals that caught our attention. Mitochondrial phylogenetic tree of all peafowl individuals shown that two green peafowl individuals (qhd-01, qhd_02) were in the blue peafowl clade. This is contrary to the results of autochromatic NJ trees. Combining the

results of autosomal Admixture and PCA, we speculate that the two green peafowl individuals may be the individuals of the hybridized individuals between blue peafowl and green peafowl. The phylogenetic tree of the W chromosome is another strong evidence of studying the maternal genetic relationship of peafowls. We found that the two green peafowl individuals are located on the blue peafowl branch on the W chromosome ML tree of 33 peafowl individuals. This further confirms our speculation.

Although the maternal inheritance of the GF-f1A individual was in the green peafowl, we also assumed that it is also a hybrid individual because the autosomes show a similar ancestry distribution to the other two individuals.

Hybrid green peafowl individuals in the Zoo

In the wild, the habitats of the blue peafowl and the green peafowl do not overlap (Fig. 2A) [3, 115]. The number of wild green peafowls is extremely rare, with reports indicating that their total population is less than 500 [12]. Although blue peafowls are not reproductively isolated from green peafowls, no hybrid green peafowls have been reported in the wild. Through phylogenetic tree analysis of autosomal, mitochondrial and W chromosomes sequence, we discovered three putative green peafowl individuals hybridizing blue peafowls and green peafowls. Next, we will further verifiy this hybridization event and analyze the proportion of blue peafowl blood in these samples.

Migration events between green peafowls and blue peafowls populations were estimated using TreeMix and constructing ML phylogenetic trees. When the migration event was set to an optimal value of 3 ($m = 3$), gene flow from blue peafowl and green peafowl occurred (Fig. 2E). To investigate the relationship of the three green peafowl individuals to blue peafowl, we selected core groups of

green peafowl respectively based on the genetic structure analysis using ADMIXTURE and a phylogenetic analysis using f3 statistics (Table. S12 and S13). Then, we performed D statistics and f3 statistics for all individuals based on SNP frequency differences. In D statistical analysis, we set (P1, P2, P3, O) as (green peafowl individuals, putative hybrid individuals, blue peafowl individuals, chicken). The D statistics showed signifcantly introgression events ($Z > 3$, $P < 0.001$) from all putative hybrid individuals. The f3 statistics and D statistics also confirmed that the three green peafowl individuals shared the most derived polymorphisms with green peafowl. These results corroborated the PCA and ADMIXTURE results that demonstrated the existence of hybridization between blue peafowl and green peafowl.

Using the core populations of the two species as a reference, we showed that hybrid green peafowl individuals share allele SNPs and haplotype-region with the blue peafowl (Fig. 4A and S19). Two individuals (qhd-01, qhd_02) with similar shared allele profiles and another individual (GF-f1A) who differs from them, possibly due to differences in genetic background due to geographic location. We also used the same sample to calculate the proportion of ancestry of blue peafowl among the three hybrid individuals (Fig. 4A and S20). Among them, the proportion of ancestry of blue peafowl in qhd_02 individual accounted for the highest 33.74%, which indicated that this individual was a hybrid individual of one or two generations of blue peafowl to green peafowl. Although the mitochondrial DNA of GF-f1A individual is in the green peafowl clade, the proportion of ancestry of blue peafowl in its genome reached 29.45%. Phylogenetic relationships inferred from ML trees constructed using mitochondrial SNPs and W chromosome SNPs (Fig. 4B, 4C and 4D) further supported these findings, indicating distinct clusters of green peafowl individuals and blue peafowl, whereas two green peafowl individuals (qhd-01, qhd_02) is located

in the blue peafowl branch.

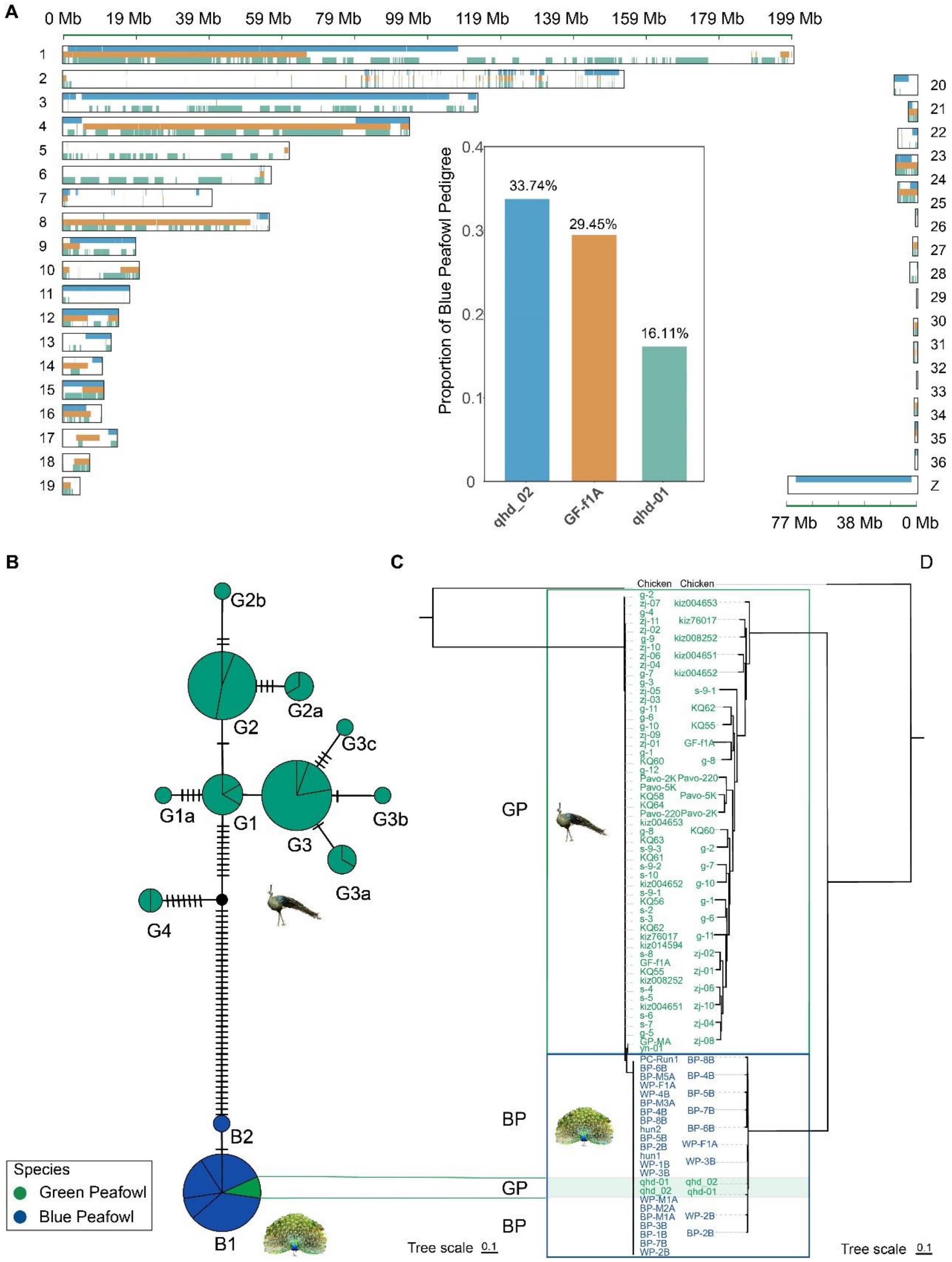

Fig. 3. Lineage distribution of hybrid green peafowl individuals in autosomes, W chromosome and mitochondrial DNA. (A) Genome-wide distribution of blue peafowl haplotypes in hybrid green peafowl individuals. The inset shows the proportion of autosomal blue peafowl ancestry in

hybrid green peafowl individuals by using Loter. (B) Median-joining haplotype network from the 76 peafowl individual mitochondrial sequence. (C) Mitochondrial phylogenetic tree of 76 peafowl individuals constructed using the ML method. (D) W chromosome phylogenetic tree of 38 female peafowls constructed using the ML method. BP represents the blue peafowl and GP represents the green peafowl.

Historical introgression regions influence blue peafowl body size and immunity

Treemix analysis of all individuals not only showed the gene flow from blue peafowl to green peafowl, but the gene flow from green peafowl to Beijing blue peafowl (Fig. 2E). Therefore, blue peafowls and green peafowls may have historical introgression events. We removed the individuals identified as hybrids, and the calculated treemix showed the gene flow from green peafowl to Beijing blue peafowl occurred when the migration event was set to 1(m = 1) (Fig. 4A). To further analyze introgression events, we performed f3 statistics and D statistic based on SNP frequency difference. Both of the two methods showed significant introgression events ($Z > 3$) (Fig. 4B).

The introgression region was further defined by identifying 267 genes in the top 5% D-value window positions and presuming these to be candidates for introgression (Fig. S21 and Table. S14). The GO and KEGG annotation showed that the introgression genes were enriched for significantly introgression genes mainly included cytoplasm (GO:0005737), cellular senescence KEGG PATHWAY (gga04218) (Fig. S22).

Among these genes, IGFBP1 and IGFBP3, members of the Insulin-like Growth Factor-Binding Protein (IGFBP) family, were highlighted due to their association with body size [116, 117].

The degree and direction of introgression in the BPB blue peafowl was further detected by calculating the *Fst* between BPB blue peafowl individuals and other blue peafowl, BPB blue peafowl individuals and green peafowl individuals, respectively, based on the results of D statistic analysis (Fig. 4B). We obtained a region located on chromesome 2 (from 55304837 bp to 55337497 bp), which contained the candidate gene *IGFBP1* and *IGPBP3*. In the region, the BPB pop1 (BP-1B, BP-4B, BP-7B, BP-8B) and green peafowl were poorly differentiated (*Fst*=0), BPB pop2 (BP-2B, BP-3B, BP-5B, BP-6B) and blue peafowl were highly differentiated (*Fst*=0.89) (Fig. 4C). An ML tree was constructed using *IGFBP1* and *IGPBP3* gene sequences (Fig. 4E). In the ML tree, BP-1B, BP-4B, BP-8B, BP-7B clustered with green peafowl branch and other blue peafowl formed separate branches. Haplotypes in this region also showed similar results (Fig. 4D and 4E).

In addition, we retrieved several immune response related genes (*IL6*, *IL12B*, *IL25*, *NFATC1*, *DROSHA* and *CUL1*) in introgressed regions that may help blue peafowl fight disease. Among all immune genes, *IL-6* gene is involved in multiple immune pathways and is a chicken heat shock protein [118, 119]. Studies have found that *IL-6* is involved in the innate immunity of poultry diseases such as Newcastle disease (ND) [120].

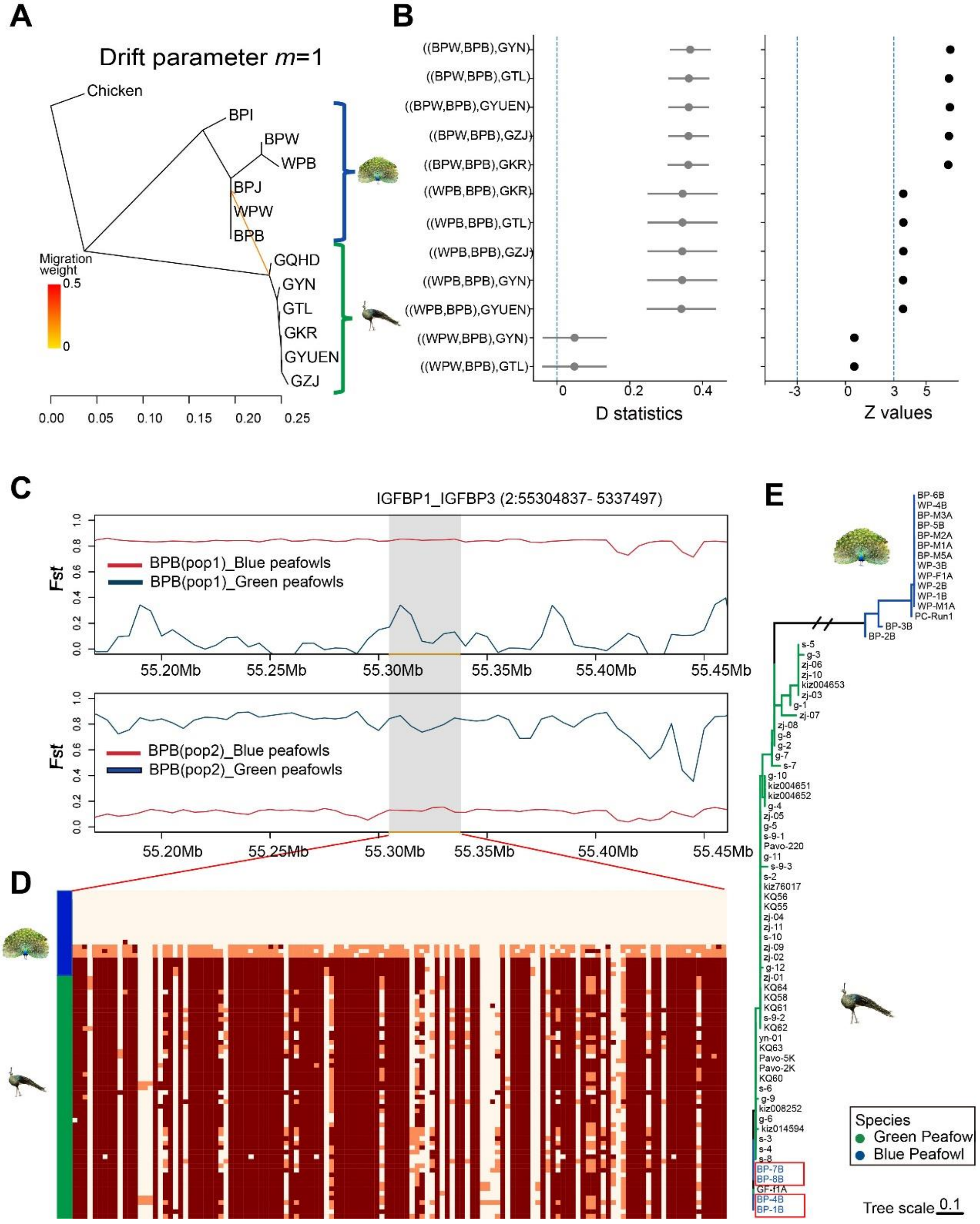

Fig. 4. Introgression analysis of peafowl population. (A) Genetic migration inferred using TreeMix (m = 1). (B) D-statistics for introgression between BPB blue peafowl population and green peafowl population. (C) *Fst* between BPB blue peafowl individuals and the green peafowl populations. The BPB blue peafowl samples were divided into two groups based on *Fst* values, BPB (pop1) includes BP-1B, BP-4B, BP-7B and BP-8B; BPB (pop2) includes BP-2B, BP-3B, BP-5B

and BP-6B. The blue line represents *Fst* between BPB and green peafowl, and red line represents *Fst* between BPB and blue peafowl. The gray region indicates the location of the *IGFBP1* and *IGFBP3* gene regions. (D) Introgression plot of the blue peafowl contacted based on shared allele SNPs in *IGFBP1* and *IGFBP3* gene regions across chromosome2 (from 55304837 bp to 55337497 bp). (E) Phylogenetic tree of 77 peafowl individuals constructed by 109 SNPs in *IGFBP1* and *IGFBP3* gene regions using ML method. The blue peafowl individuals in the red box have introgression in this region.

Nonsense mutation in *EDNRB2* gene creates white plumage individuals in blue peafowls

As early as 1868, Darwin reported the white peafowls and pied peafowl (blue white-flight plumage color), the mutant individuals of the blue peafowl's plumage color, which shows that the white peafowl appeared at least 150 years ago (Fig. 5E and 5F) [121]. Under artificial breeding, white peafowls have reached a state of self-sustaining population. As a striking ornamental bird, research on the genetic mechanism of plumage color in white peafowls has never ended [1]. Studies have reported that the recessive allele that controls the white feathers of peafowls is located on an autosomal chromosome, and its inheritance conforms to Mendel's law of segregation [29]. However, the molecular mechanism of white peafowl plumage color has not been elucidated due to technical limitations [1].

In this study, we compared 11 blue peafowl samples and 5 white peafowl samples from different geographic regions to identify the genomic region responsible for the emergence of mutated white peafowl individuals. We used *Fst* method to search for the causal mutations. The

most significant region was located on the 12–13 Mb interval of chromosome 4 (Fig. 5A, 5B and Table. S15). The analysis based on Absolute genetic divergence (Dxy) and Tajima's D supported a strong selective signal in this region. Through genotypic analysis of 159 mutations (SNP, Indel and CNV) in this interval, we found that the only polymorphism completely associated with peafowl white plumages was a nonsense mutation site located in the *EDNRB2* coding region (g.4:12583552 G>A) (Fig. 5C and S23). *EDNRB2* is an important key gene in the formation of bird feather color. *EDNRB2* is one of the receptors for EDNs, which is strong mitogens for melanoblasts. Kawasaki-Nishihara et al. suggested that EDN3–EDNRB2 signaling is required for normal melanoblast migration in Xenopus embryos on the basis of in vivo experiments [122]. It does not participate in the melanin synthesis pathway, but regulates the differentiation, proliferation, and migration of melanocytes [123, 124]. Therefore, we speculated that this incorrect genetic information leads to RNA degradation, known as NMD (nonsense-mediated mRNA decay) [125]. The degradation of *EDNRB2* mRNA inhibits the differentiation, proliferation and migration of peafowl melanocytes, resulting in the white peafowl individuals with white plumages and black eyes.

To confirm this assumption, we analyzed the genotypes of 11 white peafowls, 29 blue peafowls, and one pied peafowl. This SNP showed homozygous or heterozygous genotypes (G/G or G/A) in all blue peafowls and heterozygous genotypes (G/A) in pied peafowl, while all white peafowls were homozygous for the mutant (A/A) (Table. S16). These results indicate that the white plumage phenotype emerged as a result of the nonsense mutation in the *EDNRB2* gene and the white allele (A) is shown to be an incompletely dominant. RNA-seq data of three blue peafowls and three white peafowls plumage found that there was no expression of the *EDNRB2* gene in the white peafowl plumage, while the gene was highly expressed in the blue peafowl feathers. FISH (Fluorescent in

situ hybridization) experiments on this tissue showed the same results (Fig. 5H). In addition, we also found that the melanocyte marker genes (*SOX10* and *MLANA*) are not expressed in white peafowl plumage [126], but are highly expressed in blue peafowl plumage (Fig. 5I and S24). The formation and deposition of melanin mainly occurs on the amyloid fibres of melanosomes. Masson-Fontana staining found no melanin and melanocytes in white peafowl plumage tissue (Fig. 5G). The above results indicate that melanocytes do not exist in white peafowl feathers. This verified our conjecture that the nonsense mutation of the *EDNRB2* gene caused the NMD reaction in white peafowls, and the mRNA of the *EDNRB2* gene was degraded during translation, thus preventing melanocytes from being transported into plumage tissue.

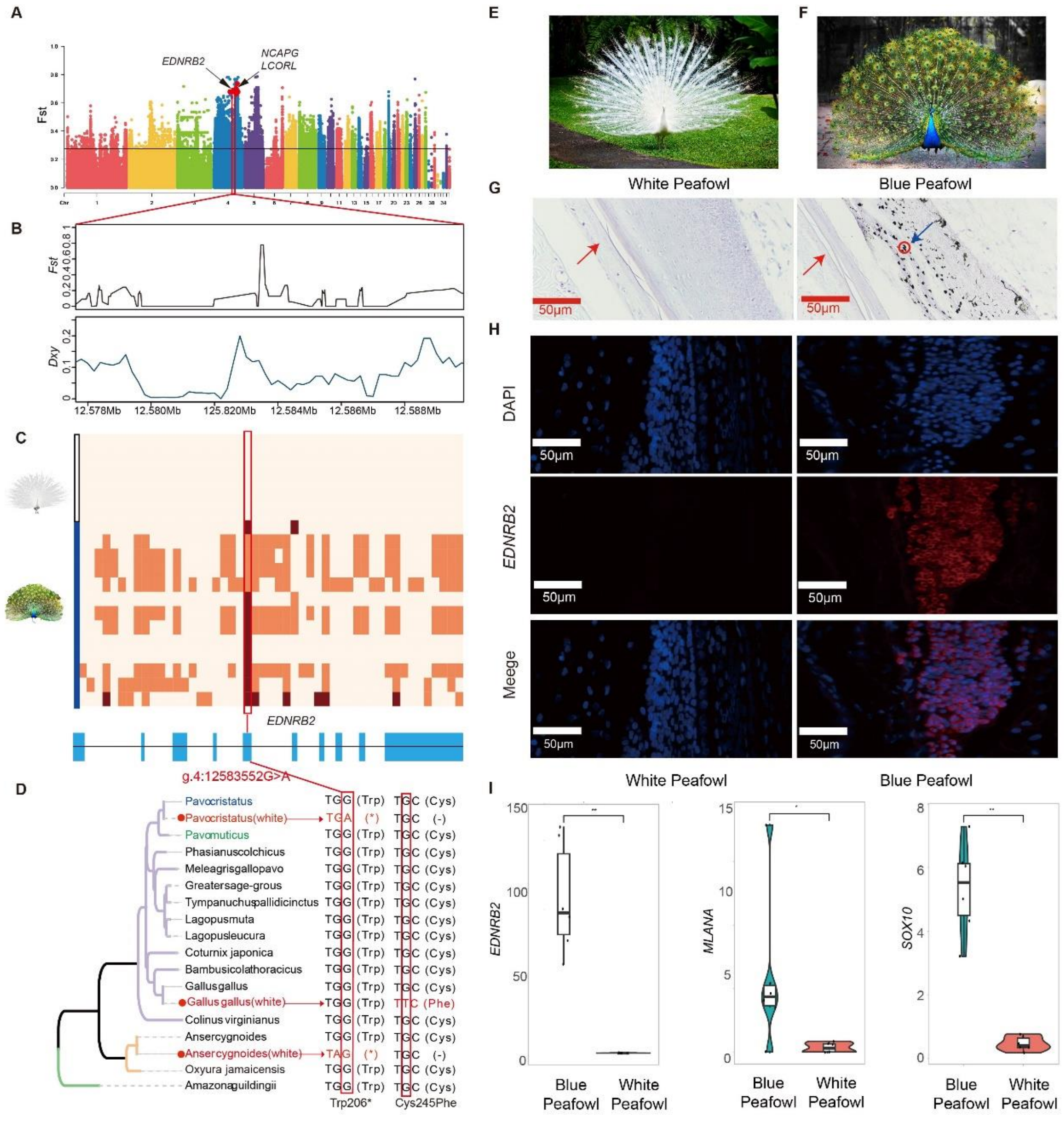

Fig. 5. White peafowl white plumage phenotype characteristics and molecular mechanism. (A) Whole genome scan with *Fst*. The red arrow indicates the genome position of the strongest selective signal for the white plumage phenotype. (B) *Fst* and Dxy corresponding to the white plumage selective sweep on chromosome 4 which encompasses the *EDNRB2* gene. (C) Plot of the haplotype structure of SNPs around the *EDNRB2* gene in all white peafowls and blue peafowls. Nucleotides with brown box represent blue peafowl homozygote genotypes, and with orange box represent heterozygote genotypes. The plot also showed the genetic structure of *EDNRB2*, and the blue box is the CDS region. The position of the red line indication is the nonsense mutation of the w white

plumage phenotype (g.4:12583552G>A), which is located in the fifth CDS region. (D) The convergence evolution of *EDNRB2* codons in different white plumage species comparison. The first convergent site is Trp206*(Stop codon). The white peafowl has a nonsense mutation at this position. White geese have a 14-BP insertion before the position, so that the codon is turned to be a stop codon in this position. The second convergent site is Cys245Phe, which only occurs on the white plumage chickens. (E) A white peafowl with white plumages and black eyes. (F) A Blue peafowl with black and blue plumages. (G) Micrographs of plumage sections from white and blue peafowls stained with the Masson-Fontana. The red arrow is the plumage tube wall. Blue arrows are melanosome and melanocytes. (H) FISH was performed to observed the cellular location of *EDNRB2* in plumage tube. Nuclei were stained with DAPI. Scale bar = 50 μm. (I) *EDNRB2* and melanocyte maker genes (*SOX10* and *MLANA*) are expressed in white peafowl and blue peafowl plumages, respectively. Genetic expression is measured by transcripts per million (TPM). Data were indicated as mean ± SEM (n = 3), ns $P \geqslant 0.05$, * $P < 0.05$, * * $P < 0.01$.

Convergent selection for the White feather in geese, chicken and peafowls

The white plumage trait in birds represents a common phenotypic convergence, particularly observable in poultry breed [127, 128]. In these species, various colorful birds have been domesticated, leading to the emergence of white plumage individuals due to the domestication syndrome) [129]. However, the molecular mechanisms underlying this white plumage trait differ across species. For instance, in waterfowl, white plumage in duck results from alternative splicing of the *MITF* gene [130, 131]. whereas in geese, it is caused by mutations in the *EDNRB2* gene [31].

In chickens, dominant white plumage is governed by the *PMEL17* gene [127], while Tyrosine-Independent white plumage is controlled by the *EDNRB2* gene [30]. Notably, both white plumage chickens and geese regulated by the *EDNRB2* gene exhibit predominantly white plumage across their bodies but possess black eyes.

In this study, in order to explore the underlying molecular regulatory mechanism of this phenotypic convergence, we compared the *EDNRB2* gene sequences in 15 species of birds, including white plumage chickens and white plumage geese. In the case of Minohiki chickens, a nonsynonymous mutation (G>T) associated with the white feather phenotype was identified in the coding region (CDS) of the *EDNRB2* gene [30]. This mutation leads to a functional defect in *EDNRB2*'s ability to bind to EDN ligands, which in turn interferes with melanocyte differentiation, proliferation, and migration (Fig. 5D). In the Gang geese, a 14-base pair insertion caused a frameshift mutation in the gene's coding region, resulting in a premature stop codon (TAG) [31]. This insertion also triggered the NMD (nonsense-mediated decay) mechanism, leading to the absence of detectable mRNA in white geese individuals.

Although these findings highlight the involvement of the same causative gene (*EDNRB2*) is involved, the mutations responsible for white feather coloration in the three species occurred independently after the species diverged. Notably, the mRNA expression of *EDNRB2* in geese is also lost due to nonsense mutations caused by frameshift mutations, thus indicating that the molecular mechanisms of white feathers in peafowls and geese are very similar.

Discussion

In this study, we report the first chromosome-level de novo genome assembly for the blue

peafowl with the 95 Mb Scaffold N50 of the assembly which was substantially higher than those obtained in previous studies and green peafowl genome [1, 5, 7]. We obtained more pairs of macrochromosomes than in karyotype study of the blue peafowl, which only had 8 pairs of macrochromosomes [98]. The genome synteny and collinearity analysis with chicken and other birds and the evaluation of various algorithms prove that the current blue peafowl genome assembly is of high quality, with consistency, accuracy and completeness. Although we achieved partial chromosome-level assembly, due to the GC-richness, specific repeats, high microchromosome mutation rate in birds [132], and lack of cytogenetic support, the peafowl microchromosomes and W chromosome require a combination of different data sources including optical mapping and linked reads to improve the assembly quality [133, 134]. In general, this chromosome-level genome of blue peafowl strongly supports the subsequent comparative genomic and population structure analysis. The karyotypes of birds are relatively conserved, especially in the pheasant family [101, 135]. This pattern was confirmed in our analysis of the conserved synteny between turkey and chicken. We only detected chromosome fusions on chromosomes 6 and 8.

Regarding the relationship between peafowl and chicken and turkey, there are two views: (1) Peafowl is closer to turkey than chicken [1, 136]; (2) Peafowl is closer to chicken than turkey [5, 6, 137]. To resolve previous inconsistencies concerning peafowl phylogenetic relationships, we reconstructed three phylogenomic trees from one-to-one orthologs datasets, four-fold degenerate sites datasets and mitochondrial sequence datasets, respectively. All phylogenetic trees supported that peafowl was closer to chicken than turkey.

We found that the appearance of peafowls (~48 Mya) coincided with the rapid speciation of pheasants based on multiple species fossil time correction or site spectrum. Rapid speciation has

occurred many times during the evolution of birds, and these events have resulted in a large number of incomplete lineage sorting (ILS) among bird species. Combined with the timing of the peafowl's emergenc and a number of phylogenetic inconsistencies across the genome, we believe that ILS is one of the reasons for the huge controversy over the phylogenetic relationships of peafowls with chicken and turkey. Additionally, we also found evidence of gene flow between the ancestral nodes of peafowl and turkey, which indicates that gene flow also contributes to the difficulties in reconstructing the phylogenetic tree among these three species.

Our results indicated that blue peafowl and wild green peafowl have different population histories. The wild green peafowl experienced rapid population expansion followed by a dramatic population decline during the LGP which consistent with the results of Dong et al [138]. And the most other threatened species experienced a similar history of group dynamics [5, 68, 139-141]. The effective group size of blue peafowl is higher than that of blue peafowl before LIG. Moreover, the effective group size of the blue peafowl gradually became smaller after the LIG, which is very similar to the historical dynamics of domestic chickens [142]. The different group histories of the two species may be an important factor in the current divergent fate of the two species. Although the wild green peafowl experienced a significant decrease of genetic diversity in the last 50 years [138], the genetic diversity of wild green peafowl populations is still significantly higher than that of captive blue peafowl.

Habitat loss and fragmentation has long been considered the primary cause for biodiversity loss and wild animal extinction worldwide [143]. Over the past three decades, the endangered wild green peafowl as the only peafowl native to China, has experienced sharp population declines and faced the problem of habitat fragmentation [11, 12]. In order to respond to the Convention on

Biological Diversity and save the endangered wild green peafowl [144], the Chinese government has issued a number of protection policies, including banning poaching and captive breeding [15, 18]. The wild green peafowl can hybridize with blue peafowl and produce fertile off-spring that can backcross with ancestral species [145]. However, since the habitats of the wild green peafowl and wild blue peafowl do not overlap, and the number of green peafowl is extremely small, no hybrid green peafowls have been reported in the wild [12, 115]. Some researchers in Southeast Asia have recognized the importance of preventing breeding blue peafowls from interbreeding with wild green peafowls to protect the endangered green peafowl. They have suggested that the breeding of blue peafowl near the distribution area (including the potential distribution area) of the wild green peafowl should be prohibited [146]. When blue peafowls and green peafowls are kept in captivity in zoos, their geographical isolation vanishes, significantly increasing the likelihood of interbreeding. In this study, through autosomal and mitochondrial analysis of peafowl populations and validation using multiple methods, we found three green peafowl individuals hybridizing with blue peafowl. The proportion of ancestry of blue peafowl among the three hybrid green peafowl individuals is less than 50%. Obviously, these three peafowls have undergone backcrossing after hybridization. Moreover, consistent with the description of Du et al., hybrid green peafowls were indistinguishable from purebred green peafowls in appearance [145]. Over the years, for endangered species, captive breeding for reintroduction is potentially an important measure for localized population restoration [145]. Yet, the peril to endangered animal conservation posed by hybridization with closely related species in captivity and their subsequent unregulated release has been disregarded, such as red wolf Canis rufus hybridizing with coyote Canis latrans, bison Bison bison with domestic cattle (Bos spp.) or banded pig (Sus scrofa vitattus) with the endangered Java

warty pig (Sus verrucosus) [22-25]. Hence, we advocate for segregating the habitats of green peafowls and blue peafowls during artificial breeding to deter hybridization. At the same time, we suggest that purebred identification must be carried out before releasing green peafowls to prevent genetic contamination.

In addition to recent hybridization, we also found historical introgression events between blue peafowl and green peafowl, with blue peafowl having gene flow from green peafowl. Our results showed introgression from green peafowl to the blue peafowl, which contained 267 candidate protein-coding genes, including gene related to neurodevelopment, cell signaling, transcription, translation, and skeletal development. The sequence of *IGFBP1* and *IGFBP3* had the strong signals among these genes. Both of the genes which conserved in birds are member of the insulin-like growth factor binding protein (IGFBP) family and encodes a protein with an IGFBP domain and a thyroglobulin type-I domain [116]. The IGFBP family mediates IGF effects by enhancing or dampening IGF signaling. This occurs by either increasing IGF-receptor affinity, physically sequestering it to prevent receptor binding, or extending IGF's half-life in circulation [117]. Additionally, many IGFBPs can act independently to induce cellular activity [147]. A large number of studies have found that *IGFBP3* affects growth traits in common domestic animals, such as pig, cattle and sheep [148-150]. Moreover, the IGFBPs system is highly conserved in chicken, and is involved in the regulation of egg production, growth, and carcass traits. *IGFBP3* participates in myogenic cell proliferation and myoblast differentiation, and the SNPs in the *IGFBP3* promoter region were significantly associated with body weight, breast muscle weight, and leg muscle weight [151]. The genes (*IGF2BP3*, *TGBR1*, *ISPD*, *MEOX2*, *GLI3* and *MC4R*) related to body size in blue peafowl were also found to have introgression areas from green peafowl. The size of the peafowl is

larger in the pheasant family, and the size of the green peafowl is even larger than that of the blue peafowl [152]. These body size-related genes may be an important reason for the current larger size of the blue peafowl, but whether it is a factor affecting the viability of the blue peafowl needs further research. The immune-related genes found in introgressed regions are likely the result of adaptive introgression which possibly improved the viability of the blue peafowl, consistent with studies in common domesticated animals such as chickens and cattle [153, 154].

Molecular genetic studies in a variety of organisms highlight the repeatability of phenotypic change by changes in the same genes [155, 156]. Among vertebrates, this trend is especially pronounced in studies of pigmentation diversity. One dramatic example of this repeatability is the dilution of the coloration typical of the Dun phenotype displays very similar microscopic and macroscopic features in the Perissodactyla horses and donkeys [157]. *TBX3* is responsible for the Dun pattern of pigmentation in both species. The causal mutation of the non-Dun phenotype in donkeys is a 1 bp deletion with a probable regulatory effect. Similarly, in horses, the non-Dun phenotype is explained by two deletions with regulatory effects [158]. In another study, Lopes et al. provided evidence that the yellow and red plumages pigmentation of canaries and finches is caused by C(4)-oxygenation of carotenoids by the cytochrome P450 enzyme *CYP2J19* [159]. This chemical modification increases the length of the conjugated part of the carotenoid molecule, causing a red shift in its absorbance spectrum [160]. These examples also highlight an emerging pattern: Not only are the same genes involved in convergent evolution among populations and species, but so are specific molecular regions [161]. Here, we showed that the *EDNRB2* gene was convergently selected in blue peafowls, chickens and geese. The three types of white-feathered birds have different mutation types in the same gene. The reason for the white feathers in chickens is that the

protein molecular structure of the *EDNRB2* gene has changed [30]. Although the mutation sites in peafowls and geese are different, they ultimately cause premature termination of codons which triggering the NMD molecular mechanism of organisms and preventing melanocytes from being transported to plumages [31]. Our results demonstrate that not only the same gene regions are involved in convergent evolution between populations and species, but also specific molecular response mechanisms resulting from mutations in this region are involved. Although this particular case cannot be generalized to other phenotypes, it emphasizes the need to precisely clarify the role of convergent evolution in the fixation of Mendelian phenotypes.

Acknowledgments

We would like to thank Professor Judith Mank, Department of Zoology, University of British Columbia, for her valuable comments and suggestions on this paper. Thanks to the Beijing Zoo for providing the peafowl blood samples and photos. Thanks for the supporting by High-performance Computing Platform of China Agricultural University.

Authors’ Contributions

Formal analysis: Gang Wang.

Writing and editing: Gang Wang.

Investigation: Zhonghua Ning, Xufang Ren.

Data curation: Gang Wang, Xinye Zhang,

Collect samples: Liping Ban, Xiurong Zhao, Xufang Ren, Anqi Chen, Li Zhang, Yan Lu, Zhihua Jiang, Xiaoyu Zhao, Junhui Wen, Yalan Zhang, Xue Cheng, Huie Wang, Wenting Dai, Yong Liu.

Methodology: Xiurong Zhao, Xufang Ren.

Project administration: Lujiang Qu.

Supervision: Lujiang Qu.

Validation and Writing—review: Lujiang Qu.

Declarations of interets

The authors declare no competing interests.

Funding

This research was funded by the Beijing Agriculture Innovation Consortium (BAIC06-2023).

Supplementary Information

Fig. S1. Pipeline of the draft genome assembly and genome annotation of blue peafowl (WP-1).

Fig. S2. Estimation of the genome size of the blue peafowl by K-mer analysis.

Fig. S3. Hi-C-based chromosome-level assemblies of blue peafowl genome and green peafowl.

Fig. S4. BUSCO assesses the completeness of published genomes and this assembled genome.

Fig. S5. Genome synteny and collinearity among the blue peafowl and Turkey.

Fig. S6. Mitochondrial genome map of blue peafowl.

Fig. S7. ML phylogenetic tree of 15 birds inferred using single-copy orthologous genes.

Fig. S8. ML phylogenetic tree and molecular clock dating analysis of 8 birds based on mitochondria genomes.

Fig. S9. Introgression between peafowl, chicken and turkey calculated using Dsuite heuristic approach.

Fig. S10. Population size history inference of blue peafowl (BP) and green peafowl (GP).

Fig. S11. Group history dynamic simulation of blue peafowl and green peafowl by using DaDi.

Fig. S12. KEGG pathway enrichment analysis of the expansion gene family of peafowl.

Fig. S13. KEGG pathway enrichment analysis of PSG and RGs of peafowl. KEGG pathways with P-value < 0.05 were shown.

Fig. S14. Variation annotation information of all peafowl individual.

Fig. S15. Group Structure of blue peafowl and green Peafowl.

Fig. S16. Nucleotide diversity of blue peafowl and green peafowl.

Fig. S17. Linkage disequilibrium in peafowl groups with individuals greater than two.

Fig. S18. Runs of homozygosity (ROH) in peafowl groups with individuals greater than two.

Fig. S19. Each chromosome distribution of shared alleles SNPs between blue peafowl (blue) and green peafowl (green) in hybrid green peafowl individuals.

Fig. S20. Comparison of hybrid individual green peafowl and purebred green peafowl.

Fig. S21. Manhattan plot of BPB blue peafowl group introgression with a window of 2500 SNPs and a step size of 500 SNPs (P1, P2, P3, O).

Fig. S22. Enrichment analysis of green peafowl introgression regions in BPB blue peafowl group.

Fig. S23. Caused mutation of the EDNRB2 gene locus (chr4: g.12583552 G>A) for the white plumage trait in white peafowls.

Fig. S24. Bar plot of differential mRNA expression (log2-transformed fold change) and

(Transcripts per million (TPM)) of pigmentation-related genes expressed in the plumage of white peafowls (n = 6) versus blue peafowls (n = 6).

Table. S1. Statistics of genome assembly data of blue peafowl (WP-1).

Table. S2. Summary of de novo genome assembly of blue peafowl.

Table. S3. Assembly assessment of completeness using BUSCOs.

Table. S4. Statistics of repeats in our assembled genome.

Table. S5. Statistics of non-coding RNAs in the assembly of peafowl.

Table. S6. Four model paraments simulated group history dynamic of blue peafowl and green peafowl by using DaDi.

Table. S7. PGs (positive selection gene) of peafowl by using the branch-site models.

Table. S8. PSG (positive selection gene) and RGs (rapidly evolving genes) of green peafowl by using the branch-site models and branch models.

Table. S9. PSG (positive selection gene) and RGs (rapidly evolving genes) of blue peafowl by using the branch-site models and branch models.

Table. S10. All samples information.

Table. S11. Sequencing depth and coverage of all samples.

Table. S12. *f*3-statictis of peafowl groups.

Table. S13. "core" group individuals of green peafowl and blue peafowl by using f3 statistics and ADMIXTURE.

Table. S14. D statistics of introgression regions (BPW, BPB, GYN, chicken).

Table. S15. *Fst* value of blue peafowl and white peafowl.

Table. S16 Table.All variations within 10kb before and after the SNP (chr4:g.12583552 G>A).

No CNV was found in the interval.

Table. S17. Genotype distribution of the short deletion at chromosome 4 (chr4: g.12583552 G>A) in Blue and White peafowls.

Data and code availability

The datasets presented in this study can be found in online repositories. The genome assembly and corresponding sequencing data were deposited at CNGB Sequence Archive (CNSA) of China National GeneBank DataBase (CNGBdb) with accession number CNP0004612. The whole sequencing data and green peafowl Hi-C data collected were from the BioProject accession number CNP0002498, PRJNA665082, PRJNA340135 and PRJNA644939. RNA sequencing data collected were from the BioProject accession number PRJNA661158 and PRJNA271731.

The codes for reproducing the results are also provided in an online repository: https://github.com/hebuzailikaideshihou/Peafowl_Analysis.

Any additional information required to reanalyze the data reported in this paper is available from the lead contact upon request.

References

1. Liu S, Chen H, Ouyang J, Huang M, Zhang H, Zheng S, et al. A high-quality assembly reveals genomic characteristics, phylogenetic status, and causal genes for leucism plumage of Indian peafowl. Gigascience. 2022;11 doi:https://doi.org/10.1093/gigascience/giac018.
2. Gadagkar R. Is the peacock merely beautiful or also honest? Current Science. 2003:1012-20. doi:https://www.jstor.org/stable/24108784.
3. Desai A. Cry, the peacock. Orient paperbacks; 1983.
4. Hernowo JB, Mardiastuti ANI, Alikodra HS and Kusmana C. Behavior ecology of the javan green peafowl (Pavo muticus muticus Linnaeus 1758) in Baluran and Alas Purwo national park, East Java. HAYATI Journal of Biosciences. 2011;18 4:164-76.

doi:https://doi.org/10.4308/hjb.18.4.164.

5. Jaiswal SK, Gupta A, Saxena R, Prasoodanan VPK, Sharma AK, Mittal P, et al. Genome Sequence of Peacock Reveals the Peculiar Case of a Glittering Bird. Frontiers in genetics. 2018;9:392-. doi:https://doi.org/10.3389/fgene.2018.00392.

6. Dhar R, Seethy A, Pethusamy K, Singh S, Rohil V, Purkayastha K, et al. De novo assembly of the Indian blue peacock (Pavo cristatus) genome using Oxford Nanopore technology and Illumina sequencing. Gigascience. 2019;8 5 doi:https://doi.org/10.1093/gigascience/giz038.

7. Zhang X, Lin C, Li H, Liu S, Wang Q, Yang S, et al. Chromosome-Level Genome Assembly of the Green Peafowl (Pavo muticus). Genome Biol Evol. 2022;14 2 doi:https://doi.org/10.1093/gbe/evac015.

8. Harrison PW, Wright AE, Zimmer F, Dean R, Montgomery SH, Pointer MA and Mank JE. Sexual selection drives evolution and rapid turnover of male gene expression. Proc Natl Acad Sci U S A. 2015;112 14:4393-8. doi:https://doi.org/10.1073/pnas.1501339112.

9. Ramesh K and McGowan P. On the current status of Indian Peafowl Pavo cristatus (Aves: Galliformes: Phasianidae): keeping the common species common. Journal of Threatened Taxa. 2009;1 2:106-8. doi:https://doi.org/10.11609/JoTT.o1845.106-8.

10. McGowan P, Kirwan G, Del Hoyo J, Elliott A, Sargatal J, Christie DA and de Juana E. Handbook of the birds of the world alive. 2019.

11. Kong D, Wu F, Shan P, Gao J, Yan D, Luo W and Yang X. Status and distribution changes of the endangered Green Peafowl (Pavo muticus) in China over the past three decades (1990s–2017). Avian Research. 2018;9 1:1-9. doi:https://doi.org/10.1186/s40657-018-0110-0.

12. Tang W, Wang X, Yan M, Zeng G and Liang J. China's dams threaten green peafowl. Science. 2019;364 6444:943. doi:https://doi.org/10.1126/science.aax4779.

13. Pang B. Birds in``Compendium of Materia Medica''. Chinese Journal of Zoology. 1976;2:35-7.

14. McGowan PJK, Duckworth JW, Xianji W, Van Balen B, Xiaojun Y, Khan KM, et al. A review of the status of the Green Peafowl Pavo muticus and recommendations for future action. Bird Conservation International. 1998;8 4:331-48. doi:https://doi.org/10.1017/S0959270900002100.

15. Wu F, Kong DJ, Shan PF, Wang J, Kungu GN, Lu GY and Yang XJ. Ongoing green peafowl protection in China. Zool Res. 2019;40 6:580-2. doi:https://doi.org/10.24272/j.issn.2095-8137.2019.069.

16. Arenas M, Ray N, Currat M and Excoffier L. Consequences of range contractions and range shifts on molecular diversity. Mol Biol Evol. 2012;29 1:207-18. doi:https://doi.org/10.1093/molbev/msr187.

17. Zhou TC, Sha T, Irwin DM and Zhang YP. Complete mitochondrial genome of the Indian peafowl (Pavo cristatus), with phylogenetic analysis in phasianidae. Mitochondrial DNA. 2015;26 6:912-3. doi:https://doi.org/10.3109/19401736.2013.863287.

18. Gu B and Wang F. A review on the ecology and conservation biology of green peafowl (Pavo muticus). Biodiversity Science. 2021;29 11:1554. doi:https://doi.org/10.17520/biods.2021144.

19. Nei M, Maruyama T and Chakraborty R. THE BOTTLENECK EFFECT AND

GENETIC VARIABILITY IN POPULATIONS. Evolution. 1975;29 1:1-10. doi:https://doi.org/10.1111/j.1558-5646.1975.tb00807.x.

20. Rhymer JM and Simberloff D. Extinction by hybridization and introgression. Annual review of ecology and systematics. 1996;27 1:83-109. doi:https://doi.org/10.1146/annurev.ecolsys.27.1.83.

21. Lacy RC. Importance of genetic variation to the viability of mammalian populations. Journal of mammalogy. 1997;78 2:320-35. doi:https://doi.org/10.2307/1382885.

22. Adams JR, Kelly BT and Waits LP. Using faecal DNA sampling and GIS to monitor hybridization between red wolves (Canis rufus) and coyotes (Canis latrans). Molecular Ecology. 2003;12 8:2175-86. doi:https://doi.org/10.1046/j.1365-294x.2003.01895.x.

23. Freese CH, Aune KE, Boyd DP, Derr JN, Forrest SC, Gates CC, et al. Second chance for the plains bison. Biological Conservation. 2007;136 2:175-84. doi:https://doi.org/10.1016/j.biocon.2006.11.019.

24. Drygala F, Rode-Margono J, Semiadi G, Wirdateti and Frantz AC. Evidence of hybridisation between the common Indonesian banded pig (Sus scrofa vitattus) and the endangered Java warty pig (Sus verrucosus). Conservation Genetics. 2020;21:1073-8. doi:https://doi.org/10.1007/s10592-020-01304-3.

25. Adavoudi R and Pilot M. Consequences of hybridization in mammals: A systematic review. Genes. 2022;13 1:50. doi:https://doi.org/10.3390/genes13010050.

26. Harrison RG and Larson EL. Hybridization, introgression, and the nature of species boundaries. J Hered. 2014;105 Suppl 1:795-809. doi:https://doi.org/10.1093/jhered/esu033.

27. Poelstra JW, Vijay N, Bossu CM, Lantz H, Ryll B, Muller I, et al. The genomic landscape underlying phenotypic integrity in the face of gene flow in crows. Science. 2014;344 6190:1410-4. doi:https://doi.org/10.1126/science.1253226.

28. Racimo F, Sankararaman S, Nielsen R and Huerta-Sanchez E. Evidence for archaic adaptive introgression in humans. Nat Rev Genet. 2015;16 6:359-71. doi:https://doi.org/10.1038/nrg3936.

29. Somes Jr RG and Burger RE. Inheritance of the white and pied plumage color patterns in the Indian peafowl (Pavo cristatus). Journal of Heredity. 1993;84 1:57-62. doi:https://doi.org/10.1093/oxfordjournals.jhered.a111277.

30. Kinoshita K, Akiyama T, Mizutani M, Shinomiya A, Ishikawa A, Younis HH, et al. Endothelin receptor B2 (EDNRB2) is responsible for the tyrosinase-independent recessive white (mo(w)) and mottled (mo) plumage phenotypes in the chicken. PLoS One. 2014;9 1:e86361. doi:https://doi.org/10.1371/journal.pone.0086361.

31. Xi Y, Wang L, Liu H, Ma S, Li Y, Li L, et al. A 14-bp insertion in endothelin receptor B-like (EDNRB2) is associated with white plumage in Chinese geese. BMC Genomics. 2020;21 1:162. doi:https://doi.org/10.1186/s12864-020-6562-8.

32. Marcais G and Kingsford C. A fast, lock-free approach for efficient parallel counting of occurrences of k-mers. Bioinformatics. 2011;27 6:764-70. doi:https://doi.org/10.1093/bioinformatics/btr011.

33. Ranallo-Benavidez TR, Jaron KS and Schatz MC. GenomeScope 2.0 and Smudgeplot for reference-free profiling of polyploid genomes. Nat Commun. 2020;11 1:1432. doi:https://doi.org/10.1038/s41467-020-14998-3.

34. Cheng H, Concepcion GT, Feng X, Zhang H and Li H. Haplotype-resolved de novo assembly using phased assembly graphs with hifiasm. Nat Methods. 2021;18 2:170-5. doi:https://doi.org/10.1038/s41592-020-01056-5.

35. Hassani ZS, Salehi-Abargouei A, Mirzaei M, Nadjarzadeh A and Hosseinzadeh M. The association between dietary approaches to stop hypertension diet and mediterranean diet with metabolic syndrome in a large sample of Iranian adults: YaHS and TAMYZ Studies. Food Sci Nutr. 2021;9 7:3932-41. doi:https://doi.org/10.1002/fsn3.2387.

36. Zhou C, McCarthy SA and Durbin R. YaHS: yet another Hi-C scaffolding tool. Bioinformatics. 2023;39 1 doi:https://doi.org/10.1093/bioinformatics/btac808.

37. Rhie A, Walenz BP, Koren S and Phillippy AM. Merqury: reference-free quality, completeness, and phasing assessment for genome assemblies. Genome Biol. 2020;21 1:245. doi:https://doi.org/10.1186/s13059-020-02134-9.

38. Dierckxsens N, Mardulyn P and Smits G. NOVOPlasty: de novo assembly of organelle genomes from whole genome data. Nucleic Acids Res. 2017;45 4:e18. doi:https://doi.org/10.1093/nar/gkw955.

39. Flynn JM, Hubley R, Goubert C, Rosen J, Clark AG, Feschotte C and Smit AF. RepeatModeler2 for automated genomic discovery of transposable element families. Proc Natl Acad Sci U S A. 2020;117 17:9451-7. doi:https://doi.org/10.1073/pnas.1921046117.

40. Xu Z and Wang H. LTR_FINDER: an efficient tool for the prediction of full-length LTR retrotransposons. Nucleic Acids Res. 2007;35 Web Server issue:W265-8. doi:https://doi.org/10.1093/nar/gkm286.

41. Li H. Protein-to-genome alignment with miniprot. Bioinformatics. 2023;39 1 doi:10.1093/bioinformatics/btad014.

42. Kim D, Paggi JM, Park C, Bennett C and Salzberg SL. Graph-based genome alignment and genotyping with HISAT2 and HISAT-genotype. Nat Biotechnol. 2019;37 8:907-15. doi:https://doi.org/10.1038/s41587-019-0201-4.

43. Shumate A, Wong B, Pertea G and Pertea M. Improved transcriptome assembly using a hybrid of long and short reads with StringTie. PLoS Comput Biol. 2022;18 6:e1009730. doi:https://doi.org/10.1371/journal.pcbi.1009730.

44. Bruna T, Hoff KJ, Lomsadze A, Stanke M and Borodovsky M. BRAKER2: automatic eukaryotic genome annotation with GeneMark-EP+ and AUGUSTUS supported by a protein database. NAR Genom Bioinform. 2021;3 1:lqaa108. doi:https://doi.org/10.1093/nargab/lqaa108.

45. Stanke M, Diekhans M, Baertsch R and Haussler D. Using native and syntenically mapped cDNA alignments to improve de novo gene finding. Bioinformatics. 2008;24 5:637-44. doi:https://doi.org/10.1093/bioinformatics/btn013.

46. Bruna T, Lomsadze A and Borodovsky M. GeneMark-EP+: eukaryotic gene prediction with self-training in the space of genes and proteins. NAR Genom Bioinform. 2020;2 2:lqaa026. doi:https://doi.org/10.1093/nargab/lqaa026.

47. Haas BJ, Salzberg SL, Zhu W, Pertea M, Allen JE, Orvis J, et al. Automated eukaryotic gene structure annotation using EVidenceModeler and the Program to Assemble Spliced Alignments. Genome Biol. 2008;9 1:R7. doi:https://doi.org/10.1186/gb-2008-9-1-r7.

48. Rhind N, Chen Z, Yassour M, Thompson DA, Haas BJ, Habib N, et al. Comparative

functional genomics of the fission yeasts. Science. 2011;332 6032:930-6. doi:https://doi.org/10.1126/science.1203357.

49. Cantalapiedra CP, Hernandez-Plaza A, Letunic I, Bork P and Huerta-Cepas J. eggNOG-mapper v2: Functional Annotation, Orthology Assignments, and Domain Prediction at the Metagenomic Scale. Mol Biol Evol. 2021;38 12:5825-9. doi:https://doi.org/10.1093/molbev/msab293.

50. Bairoch A and Apweiler R. The SWISS-PROT protein sequence database and its supplement TrEMBL in 2000. Nucleic Acids Res. 2000;28 1:45-8. doi:https://doi.org/10.1016/j.landusepol.2015.09.002.

51. O'Leary NA, Wright MW, Brister JR, Ciufo S, Haddad D, McVeigh R, et al. Reference sequence (RefSeq) database at NCBI: current status, taxonomic expansion, and functional annotation. Nucleic Acids Res. 2016;44 D1:D733-45. doi:https://doi.org/10.1093/nar/gkv1189.

52. El-Gebali S, Mistry J, Bateman A, Eddy SR, Luciani A, Potter SC, et al. The Pfam protein families database in 2019. Nucleic Acids Res. 2019;47 D1:D427-D32. doi:https://doi.org/10.1093/nar/gky995.

53. Kanehisa M and Goto S. KEGG: kyoto encyclopedia of genes and genomes. Nucleic Acids Res. 2000;28 1:27-30. doi:https://doi.org/10.1093/nar/28.1.27.

54. Lowe TM and Eddy SR. tRNAscan-SE: a program for improved detection of transfer RNA genes in genomic sequence. Nucleic Acids Res. 1997;25 5:955-64. doi:https://doi.org/10.1093/nar/25.5.955.

55. Nawrocki EP and Eddy SR. Infernal 1.1: 100-fold faster RNA homology searches. Bioinformatics. 2013;29 22:2933-5. doi:https://doi.org/10.1093/bioinformatics/btt509.

56. Jung J, Kim JI, Jeong YS and Yi G. AGORA: organellar genome annotation from the amino acid and nucleotide references. Bioinformatics. 2018;34 15:2661-3. doi:https://doi.org/10.1093/bioinformatics/bty196.

57. Riva G and Mauri M. MuMMER: How Robotics Can Reboot Social Interaction and Customer Engagement in Shops and Malls. Cyberpsychol Behav Soc Netw. 2021;24 3:210-1. doi:https://doi.org/10.1089/cyber.2021.29210.ceu.

58. Wang Y, Tang H, Debarry JD, Tan X, Li J, Wang X, et al. MCScanX: a toolkit for detection and evolutionary analysis of gene synteny and collinearity. Nucleic Acids Res. 2012;40 7:e49. doi:https://doi.org/10.1093/nar/gkr1293.

59. He W, Yang J, Jing Y, Xu L, Yu K and Fang X. NGenomeSyn: an easy-to-use and flexible tool for publication-ready visualization of syntenic relationships across multiple genomes. Bioinformatics. 2023;39 3 doi:https://doi.org/10.1093/bioinformatics/btad121.

60. Emms DM and Kelly S. OrthoFinder: phylogenetic orthology inference for comparative genomics. Genome Biol. 2019;20 1:238. doi:https://doi.org/10.1186/s13059-019-1832-y.

61. Suyama M, Torrents D and Bork P. PAL2NAL: robust conversion of protein sequence alignments into the corresponding codon alignments. Nucleic Acids Res. 2006;34 Web Server issue:W609-12. doi:https://doi.org/10.1093/nar/gkl315.

62. Stamatakis A. RAxML version 8: a tool for phylogenetic analysis and post-analysis of large phylogenies. Bioinformatics. 2014;30 9:1312-3.

doi:https://doi.org/10.1093/bioinformatics/btu033.

63. Kumar S, Suleski M, Craig JM, Kasprowicz AE, Sanderford M, Li M, et al. TimeTree 5: An Expanded Resource for Species Divergence Times. Mol Biol Evol. 2022;39 8 doi:https://doi.org/10.1093/molbev/msac174.

64. Yang Z. PAML 4: phylogenetic analysis by maximum likelihood. Mol Biol Evol. 2007;24 8:1586-91. doi:https://doi.org/10.1093/molbev/msm088.

65. Armstrong J, Hickey G, Diekhans M, Fiddes IT, Novak AM, Deran A, et al. Progressive Cactus is a multiple-genome aligner for the thousand-genome era. Nature. 2020;587 7833:246-51. doi:https://doi.org/10.1038/s41586-020-2871-y.

66. Wright AE, Harrison PW, Zimmer F, Montgomery SH, Pointer MA and Mank JE. Variation in promiscuity and sexual selection drives avian rate of Faster-Z evolution. Mol Ecol. 2015;24 6:1218-35. doi:https://doi.org/10.1111/mec.13113.

67. Li H and Durbin R. Inference of human population history from individual whole-genome sequences. Nature. 2011;475 7357:493-6. doi:https://doi.org/10.1038/nature10231.

68. Nadachowska-Brzyska K, Li C, Smeds L, Zhang G and Ellegren H. Temporal Dynamics of Avian Populations during Pleistocene Revealed by Whole-Genome Sequences. Curr Biol. 2015;25 10:1375-80. doi:https://doi.org/10.1016/j.cub.2015.03.047.

69. Terhorst J, Kamm JA and Song YS. Robust and scalable inference of population history from hundreds of unphased whole genomes. Nat Genet. 2017;49 2:303-9. doi:https://doi.org/10.1038/ng.3748.

70. You M, Ke F, You S, Wu Z, Liu Q, He W, et al. Variation among 532 genomes unveils the origin and evolutionary history of a global insect herbivore. Nat Commun. 2020;11 1:2321. doi:https://doi.org/10.1038/s41467-020-16178-9.

71. Gutenkunst RN, Hernandez RD, Williamson SH and Bustamante CD. Inferring the joint demographic history of multiple populations from multidimensional SNP frequency data. PLoS Genet. 2009;5 10:e1000695. doi:https://doi.org/10.1371/journal.pgen.1000695.

72. Huang X, Fortier AL, Coffman AJ, Struck TJ, Irby MN, James JE, et al. Inferring Genome-Wide Correlations of Mutation Fitness Effects between Populations. Mol Biol Evol. 2021;38 10:4588-602. doi:https://doi.org/10.1093/molbev/msab162.

73. Wen J, Li H, Wang H, Yu J, Zhu T, Zhang J, et al. Origins, timing and introgression of domestic geese revealed by whole genome data. J Anim Sci Biotechnol. 2023;14 1:26. doi:https://doi.org/10.1186/s40104-022-00826-9.

74. Coffman AJ, Hsieh PH, Gravel S and Gutenkunst RN. Computationally Efficient Composite Likelihood Statistics for Demographic Inference. Mol Biol Evol. 2016;33 2:591-3. doi:https://doi.org/10.1093/molbev/msv255.

75. Mendes FK, Vanderpool D, Fulton B and Hahn MW. CAFE 5 models variation in evolutionary rates among gene families. Bioinformatics. 2021;36 22-23:5516-8. doi:https://doi.org/10.1093/bioinformatics/btaa1022.

76. Bu D, Luo H, Huo P, Wang Z, Zhang S, He Z, et al. KOBAS-i: intelligent prioritization and exploratory visualization of biological functions for gene enrichment analysis. Nucleic Acids Res. 2021;49 W1:W317-W25. doi:https://doi.org/10.1093/nar/gkab447.

77. Chen S, Zhou Y, Chen Y and Gu J. fastp: an ultra-fast all-in-one FASTQ preprocessor. Bioinformatics. 2018;34 17:i884-i90.

doi:https://doi.org/10.1093/bioinformatics/bty560.

78. Li H and Durbin R. Fast and accurate long-read alignment with Burrows-Wheeler transform. Bioinformatics. 2010;26 5:589-95. doi:https://doi.org/10.1093/bioinformatics/btp698.

79. Danecek P, Bonfield JK, Liddle J, Marshall J, Ohan V, Pollard MO, et al. Twelve years of SAMtools and BCFtools. Gigascience. 2021;10 2 doi:https://doi.org/10.1093/gigascience/giab008.

80. Okonechnikov K, Conesa A and Garcia-Alcalde F. Qualimap 2: advanced multi-sample quality control for high-throughput sequencing data. Bioinformatics. 2016;32 2:292-4. doi:https://doi.org/10.1093/bioinformatics/btv566.

81. McKenna A, Hanna M, Banks E, Sivachenko A, Cibulskis K, Kernytsky A, et al. The Genome Analysis Toolkit: a MapReduce framework for analyzing next-generation DNA sequencing data. Genome Res. 2010;20 9:1297-303. doi:https://doi.org/10.1101/gr.107524.110.

82. Cingolani P, Patel VM, Coon M, Nguyen T, Land SJ, Ruden DM and Lu X. Using Drosophila melanogaster as a Model for Genotoxic Chemical Mutational Studies with a New Program, SnpSift. Front Genet. 2012;3:35. doi:https://doi.org/10.3389/fgene.2012.00035.

83. Wang X, Zheng Z, Cai Y, Chen T, Li C, Fu W and Jiang Y. CNVcaller: highly efficient and widely applicable software for detecting copy number variations in large populations. Gigascience. 2017;6 12:1-12. doi:https://doi.org/10.1093/gigascience/gix115.

84. Purcell S, Neale B, Todd-Brown K, Thomas L, Ferreira MA, Bender D, et al. PLINK: a tool set for whole-genome association and population-based linkage analyses. Am J Hum Genet. 2007;81 3:559-75. doi:https://doi.org/10.1086/519795.

85. Alexander DH and Lange K. Enhancements to the ADMIXTURE algorithm for individual ancestry estimation. BMC Bioinformatics. 2011;12:246. doi:https://doi.org/10.1186/1471-2105-12-246.

86. Tamura K, Stecher G and Kumar S. MEGA11: Molecular Evolutionary Genetics Analysis Version 11. Mol Biol Evol. 2021;38 7:3022-7. doi:https://doi.org/10.1093/molbev/msab120.

87. Pickrell JK and Pritchard JK. Inference of population splits and mixtures from genome-wide allele frequency data. PLoS Genet. 2012;8 11:e1002967. doi:https://doi.org/10.1371/journal.pgen.1002967.

88. Danecek P, Auton A, Abecasis G, Albers CA, Banks E, DePristo MA, et al. The variant call format and VCFtools. Bioinformatics. 2011;27 15:2156-8. doi:https://doi.org/10.1093/bioinformatics/btr330.

89. Zhang C, Dong SS, Xu JY, He WM and Yang TL. PopLDdecay: a fast and effective tool for linkage disequilibrium decay analysis based on variant call format files. Bioinformatics. 2019;35 10:1786-8. doi:https://doi.org/10.1093/bioinformatics/bty875.

90. Curik I, Ferenčaković M and Sölkner J. Inbreeding and runs of homozygosity: A possible solution to an old problem. Livestock Science. 2014;166:26-34. doi:https://doi.org/10.1016/j.livsci.2014.05.034.

91. Darriba D, Taboada GL, Doallo R and Posada D. jModelTest 2: more models, new heuristics and parallel computing. Nat Methods. 2012;9 8:772. doi:https://doi.org/10.1038/nmeth.2109.

92. Dias-Alves T, Mairal J and Blum MGB. Loter: A Software Package to Infer Local Ancestry for a Wide Range of Species. Mol Biol Evol. 2018;35 9:2318-26. doi:https://doi.org/10.1093/molbev/msy126.

93. Patterson N, Moorjani P, Luo Y, Mallick S, Rohland N, Zhan Y, et al. Ancient admixture in human history. Genetics. 2012;192 3:1065-93. doi:https://doi.org/10.1016/j.gde.2016.07.002.

94. Malinsky M, Matschiner M and Svardal H. Dsuite - Fast D-statistics and related admixture evidence from VCF files. Mol Ecol Resour. 2021;21 2:584-95. doi:https://doi.org/10.1111/1755-0998.13265.

95. Moran RL, Jaggard JB, Roback EY, Kenzior A, Rohner N, Kowalko JE, et al. Hybridization underlies localized trait evolution in cavefish. iScience. 2022;25 2:103778. doi:https://doi.org/10.1016/j.isci.2022.103778.

96. Pertea G and Pertea M. GFF Utilities: GffRead and GffCompare. F1000Res. 2020;9 doi:https://doi.org/10.12688/f1000research.23297.2.

97. Love MI, Huber W and Anders S. Moderated estimation of fold change and dispersion for RNA-seq data with DESeq2. Genome Biol. 2014;15 12:550. doi:https://doi.org/10.1186/s13059-014-0550-8.

98. Saski M, Ikechi T and Makino S. A feather pulp culture technique for avian chromosomes, with notes on the chromosomes of the peafowl and the ostrich. Experientia. 1968;24 12:1292-3. doi:https://doi.org/10.1007/bf02146680.

99. Manni M, Berkeley MR, Seppey M, Simao FA and Zdobnov EM. BUSCO Update: Novel and Streamlined Workflows along with Broader and Deeper Phylogenetic Coverage for Scoring of Eukaryotic, Prokaryotic, and Viral Genomes. Mol Biol Evol. 2021;38 10:4647-54. doi:https://doi.org/10.1093/molbev/msab199.

100. Ellegren H. Evolutionary stasis: the stable chromosomes of birds. Trends Ecol Evol. 2010;25 5:283-91. doi:https://doi.org/10.1016/j.tree.2009.12.004.

101. Zhang G, Li C, Li Q, Li B, Larkin DM, Lee C, et al. Comparative genomics reveals insights into avian genome evolution and adaptation. Science. 2014;346 6215:1311-20. doi:https://doi.org/10.1126/science.1251385.

102. Kapusta A, Suh A and Feschotte C. Dynamics of genome size evolution in birds and mammals. Proc Natl Acad Sci U S A. 2017;114 8:E1460-E9. doi:https://doi.org/10.1073/pnas.1616702114.

103. Kretschmer R, Ferguson-Smith MA and de Oliveira EHC. Karyotype Evolution in Birds: From Conventional Staining to Chromosome Painting. Genes (Basel). 2018;9 4 doi:https://doi.org/10.3390/genes9040181.

104. He C, Zhao L, Xiao L, Xu K, Ding J, Zhou H, et al. Chromosome level assembly reveals a unique immune gene organization and signatures of evolution in the common pheasant. Mol Ecol Resour. 2021;21 3:897-911. doi:https://doi.org/10.1111/1755-0998.13296.

105. Asfaw B, White T, Lovejoy O, Latimer B, Simpson S and Suwa G. Australopithecus garhi: a new species of early hominid from Ethiopia. Science. 1999;284 5414:629-35. doi:10.1126/science.284.5414.629.

106. Kimball RT, Hosner PA and Braun EL. A phylogenomic supermatrix of Galliformes (Landfowl) reveals biased branch lengths. Mol Phylogenet Evol. 2021;158:107091.

doi:10.1016/j.ympev.2021.107091.

107. Suh A, Smeds L and Ellegren H. The Dynamics of Incomplete Lineage Sorting across the Ancient Adaptive Radiation of Neoavian Birds. PLoS Biol. 2015;13 8:e1002224. doi:https://doi.org/10.1371/journal.pbio.1002224.

108. Jarvis ED, Mirarab S, Aberer AJ, Li B, Houde P, Li C, et al. Whole-genome analyses resolve early branches in the tree of life of modern birds. Science. 2014;346 6215:1320-31.

109. Malinsky M, Svardal H, Tyers AM, Miska EA, Genner MJ, Turner GF and Durbin R. Whole-genome sequences of Malawi cichlids reveal multiple radiations interconnected by gene flow. Nat Ecol Evol. 2018;2 12:1940-55. doi:10.1038/s41559-018-0717-x.

110. Hyun S. Body size regulation and insulin-like growth factor signaling. Cell Mol Life Sci. 2013;70 13:2351-65. doi:https://doi.org/10.1007/s00018-013-1313-5.

111. Maridas DE, DeMambro VE, Le PT, Nagano K, Baron R, Mohan S and Rosen CJ. IGFBP-4 regulates adult skeletal growth in a sex-specific manner. J Endocrinol. 2017;233 1:131-44. doi:https://doi.org/10.1530/joe-16-0673.

112. McPherron AC and Lee SJ. Double muscling in cattle due to mutations in the myostatin gene. Proc Natl Acad Sci U S A. 1997;94 23:12457-61. doi:https://doi.org/10.1073/pnas.94.23.12457.

113. Ekblom R, French L, Slate J and Burke T. Evolutionary analysis and expression profiling of zebra finch immune genes. Genome Biol Evol. 2010;2:781-90. doi:https://doi.org/10.1093/gbe/evq061.

114. Ramakrishnan B, Viswanathan K, Tharakaraman K, Dancik V, Raman R, Babcock GJ, et al. A Structural and Mathematical Modeling Analysis of the Likelihood of Antibody-Dependent Enhancement in Influenza. Trends Microbiol. 2016;24 12:933-43. doi:https://doi.org/10.1016/j.tim.2016.09.003.

115. Kushwaha S and Kumar A. A review on Indian peafowl (Pavo cristatus) Linnaeus, 1758. J Wildl Res. 2016;4 4:42-59.

116. Hwa V, Oh Y and Rosenfeld RG. The insulin-like growth factor-binding protein (IGFBP) superfamily. Endocrine reviews. 1999;20 6:761-87. doi:https://doi.org/10.1210/edrv.20.6.0382.

117. Gui Y and Murphy LJ. Insulin-like growth factor (IGF)-binding protein-3 (IGFBP-3) binds to fibronectin (FN): demonstration of IGF-I/IGFBP-3/fn ternary complexes in human plasma. The Journal of Clinical Endocrinology & Metabolism. 2001;86 5:2104-10. doi:https://doi.org/10.1210/jcem.86.5.7472.

118. Kaiser P, Rothwell L, Goodchild M and Bumstead N. The chicken proinflammatory cytokines interleukin-1β and interleukin-6: differences in gene structure and genetic location compared with their mammalian orthologues. Animal genetics. 2004;35 3:169-75. doi:https://doi.org/10.1111/j.1365-2052.2004.01121.x.

119. Prakasam R, Fujimoto M, Takii R, Hayashida N, Takaki E, Tan K, et al. Chicken IL-6 is a heat-shock gene. FEBS letters. 2013;587 21:3541-7. doi:https://doi.org/10.1016/j.febslet.2013.09.012.

120. Zhang D, Ding Z and Xu X. Pathologic Mechanisms of the Newcastle Disease Virus. Viruses. 2023;15 4:864. doi:https://doi.org/10.3390/v15040864.

121. Darwin C. The variation of animals and plants under domestication. J. murray; 1868.

122. Kawasaki-Nishihara A, Nishihara D, Nakamura H and Yamamoto H. ET3/Ednrb2 signaling is critically involved in regulating melanophore migration in Xenopus. Dev Dyn. 2011;240 6:1454-66. doi:https://doi.org/10.1002/dvdy.22649.

123. Lahav R, Ziller C, Dupin E and Le Douarin NM. Endothelin 3 promotes neural crest cell proliferation and mediates a vast increase in melanocyte number in culture. Proc Natl Acad Sci U S A. 1996;93 9:3892-7. doi:https://doi.org/10.1073/pnas.93.9.3892.

124. Lecoin L, Sakurai T, Ngo MT, Abe Y, Yanagisawa M and Le Douarin NM. Cloning and characterization of a novel endothelin receptor subtype in the avian class. Proc Natl Acad Sci U S A. 1998;95 6:3024-9. doi:https://doi.org/10.1073/pnas.95.6.3024.

125. Kurosaki T, Popp MW and Maquat LE. Quality and quantity control of gene expression by nonsense-mediated mRNA decay. Nat Rev Mol Cell Biol. 2019;20 7:406-20. doi:https://doi.org/10.1038/s41580-019-0126-2.

126. Hu C, Li T, Xu Y, Zhang X, Li F, Bai J, et al. CellMarker 2.0: an updated database of manually curated cell markers in human/mouse and web tools based on scRNA-seq data. Nucleic Acids Res. 2023;51 D1:D870-D6. doi:https://doi.org/10.1093/nar/gkac947.

127. Kerje S, Sharma P, Gunnarsson U, Kim H, Bagchi S, Fredriksson R, et al. The Dominant white, Dun and Smoky color variants in chicken are associated with insertion/deletion polymorphisms in the PMEL17 gene. Genetics. 2004;168 3:1507-18. doi:https://doi.org/10.1534/genetics.104.027995.

128. Zhang C, Rabiee M, Sayyari E and Mirarab S. ASTRAL-III: polynomial time species tree reconstruction from partially resolved gene trees. BMC Bioinformatics. 2018;19 Suppl 6:153. doi:https://doi.org/10.1186/s12859-018-2129-y.

129. Rubio AO and Summers K. Neural crest cell genes and the domestication syndrome: A comparative analysis of selection. PLoS One. 2022;17 2:e0263830. doi:https://doi.org/10.1371/journal.pone.0263830.

130. Zhang Z, Jia Y, Almeida P, Mank JE, van Tuinen M, Wang Q, et al. Whole-genome resequencing reveals signatures of selection and timing of duck domestication. Gigascience. 2018;7 4:giy027. doi:https://doi.org/10.1093/gigascience/giy027.

131. Zhou Z, Li M, Cheng H, Fan W, Yuan Z, Gao Q, et al. An intercross population study reveals genes associated with body size and plumage color in ducks. Nat Commun. 2018;9 1:2648. doi:https://doi.org/10.1038/s41467-018-04868-4.

132. Kawagoshi T, Nishida C, Ota H, Kumazawa Y, Endo H and Matsuda Y. Molecular structures of centromeric heterochromatin and karyotypic evolution in the Siamese crocodile (Crocodylus siamensis) (Crocodylidae, Crocodylia). Chromosome Res. 2008;16 8:1119-32. doi:10.1007/s10577-008-1263-1.

133. Jarvis ED, Mirarab S, Aberer AJ, Li B, Houde P, Li C, et al. Whole-genome analyses resolve early branches in the tree of life of modern birds. Science. 2014;346 6215:1320-31. doi:10.1126/science.1253451.

134. Zheng GX, Lau BT, Schnall-Levin M, Jarosz M, Bell JM, Hindson CM, et al. Haplotyping germline and cancer genomes with high-throughput linked-read sequencing. Nat Biotechnol. 2016;34 3:303-11. doi:10.1038/nbt.3432.

135. Barros CP, Derks MFL, Mohr J, Wood BJ, Crooijmans R, Megens HJ, et al. A new haplotype-resolved turkey genome to enable turkey genetics and genomics research.

Gigascience. 2022;12 doi:10.1093/gigascience/giad051.

136. Kaiser VB, van Tuinen M and Ellegren H. Insertion events of CR1 retrotransposable elements elucidate the phylogenetic branching order in galliform birds. Mol Biol Evol. 2007;24 1:338-47. doi:https://doi.org/10.1093/molbev/msl164.

137. Wang N, Kimball RT, Braun EL, Liang B and Zhang Z. Ancestral range reconstruction of Galliformes: the effects of topology and taxon sampling. Journal of Biogeography. 2017;44 1:122-35. doi:https://doi.org/10.1111/jbi.12782.

138. Dong F, Kuo HC, Chen GL, Wu F, Shan PF, Wang J, et al. Population genomic, climatic and anthropogenic evidence suggest the role of human forces in endangerment of green peafowl (Pavo muticus). Proc Biol Sci. 2021;288 1948:20210073. doi:https://doi.org/10.1098/rspb.2021.0073.

139. Hung C-M, Shaner P-JL, Zink RM, Liu W-C, Chu T-C, Huang W-S and Li S-H. Drastic population fluctuations explain the rapid extinction of the passenger pigeon. Proceedings of the National Academy of Sciences. 2014;111 29:10636-41. doi:https://doi.org/10.1073/pnas.1401526111.

140. Prüfer K, Racimo F, Patterson N, Jay F, Sankararaman S, Sawyer S, et al. The complete genome sequence of a Neanderthal from the Altai Mountains. Nature. 2014;505 7481:43-9. doi:https://doi.org/10.1038/nature12886.

141. Zhao Y-P, Fan G, Yin P-P, Sun S, Li N, Hong X, et al. Resequencing 545 ginkgo genomes across the world reveals the evolutionary history of the living fossil. Nature Communications. 2019;10 1:4201. doi:https://doi.org/10.1038/s41467-019-12133-5.

142. Shi S, Shao D, Yang L, Liang Q, Han W, Xue Q, et al. Whole genome analyses reveal novel genes associated with chicken adaptation to tropical and frigid environments. J Adv Res. 2023;47:13-25. doi:https://doi.org/10.1016/j.jare.2022.07.005.

143. Wilson MC, Chen X-Y, Corlett RT, Didham RK, Ding P, Holt RD, et al. Habitat fragmentation and biodiversity conservation: key findings and future challenges. Springer, 2016, p. 219-27.

144. Bai Y, Jiang B, Wang M, Li H, Alatalo JM and Huang S. New ecological redline policy (ERP) to secure ecosystem services in China. Land Use Policy. 2016;55:348-51.

145. Du HY, Zhang XY, Dinh TD, Ma Y, Zong C, Li GL, et al. Identification of hybrid green peafowl using mitochondrial and nuclear markers. Conservation Genetics Resources. 2020;12:669-83. doi:https://doi.org/10.1007/s12686-020-01159-3.

146. Goes F. The status and distribution of green peafowl Pavo muticus in Cambodia. Cambodian Journal of Natural History. 2009;2009:7-15.

147. Yamada PM and Lee K-W. Perspectives in mammalian IGFBP-3 biology: local vs. systemic action. American Journal of Physiology-Cell Physiology. 2009;296 5:C954-C76. doi:https://doi.org/10.1152/ajpcell.00598.2008.

148. Cong R, Qu X, Zhang H, Hu Y, Ye S, Cai D, et al. Maternal high-protein diet modulates hepatic growth axis in weaning piglets by reprogramming the IGFBP-3 gene. European Journal of Nutrition. 2020;59:2497-506. doi:https://doi.org/10.1007/s00394-019-02097-z.

149. Schlee P, Graml R, Schallenberger E, Schams D, Rottmann O, Olbrich-Bludau A and Pirchner F. Growth hormone and insulin-like growth factor I concentrations in bulls of

various growth hormone genotypes. Theoretical and Applied Genetics. 1994;88:497-500. doi:https://doi.org/10.1007/bf00223667.

150. Shen M, Wang W-J, Yang Y-L, Gan S-Q, He Q-H, Zhang Y-S, et al. A novel polymorphism of IGFBP-3 gene and its relationship with several wool traits in Chinese Merino sheep. Yi Chuan= Hereditas. 2008;30 9:1182-6. doi:https://doi.org/10.3724/sp.j.1005.2008.01182.

151. Guo Y, Zhang K, Geng W, Chen B, Wang D, Wang Z, et al. Evolutionary analysis and functional characterization reveal the role of the insulin-like growth factor system in a diversified selection of chickens (Gallus gallus). Poultry Science. 2023;102 3:102411. doi:https://doi.org/10.1016/j.psj.2022.102411.

152. Talha MMH, Mia MM, Momu JM, Rahman MU, Ahmad M, Adnan MR, et al. Morphometric, productive and reproductive traits of Indian peafowl (Pavo cristatus) in Bangladesh. International Journal of Development Research. 2018;8 02:19039-43.

153. Chen N, Cai Y, Chen Q, Li R, Wang K, Huang Y, et al. Whole-genome resequencing reveals world-wide ancestry and adaptive introgression events of domesticated cattle in East Asia. Nature Communications. 2018;9 1:2337. doi:https://doi.org/10.1038/s41467-018-04737-0.

154. Lawal RA, Martin SH, Vanmechelen K, Vereijken A, Silva P, Al-Atiyat RM, et al. The wild species genome ancestry of domestic chickens. BMC biology. 2020;18 1:1-18. doi:https://doi.org/10.1186/s12915-020-0738-1.

155. Gompel N and Prud'Homme B. The causes of repeated genetic evolution. Dev Biol. 2009;332 1:36-47. doi:https://doi.org/10.1016/j.ydbio.2009.04.040.

156. Christin PA, Weinreich DM and Besnard G. Causes and evolutionary significance of genetic convergence. Trends Genet. 2010;26 9:400-5. doi:https://doi.org/10.1016/j.tig.2010.06.005.

157. Wang C, Li H, Guo Y, Huang J, Sun Y, Min J, et al. Donkey genomes provide new insights into domestication and selection for coat color. Nat Commun. 2020;11 1:6014. doi:https://doi.org/10.1038/s41467-020-19813-7.

158. Imsland F, McGowan K, Rubin CJ, Henegar C, Sundstrom E, Berglund J, et al. Regulatory mutations in TBX3 disrupt asymmetric hair pigmentation that underlies Dun camouflage color in horses. Nat Genet. 2016;48 2:152-8. doi:https://doi.org/10.1038/ng.3475.

159. Lopes RJ, Johnson JD, Toomey MB, Ferreira MS, Araujo PM, Melo-Ferreira J, et al. Genetic Basis for Red Coloration in Birds. Curr Biol. 2016;26 11:1427-34. doi:https://doi.org/10.1016/j.cub.2016.03.076.

160. Mundy NI, Stapley J, Bennison C, Tucker R, Twyman H, Kim KW, et al. Red Carotenoid Coloration in the Zebra Finch Is Controlled by a Cytochrome P450 Gene Cluster. Curr Biol. 2016;26 11:1435-40. doi:https://doi.org/10.1016/j.cub.2016.04.047.

161. Vickrey AI, Domyan ET, Horvath MP and Shapiro MD. Convergent Evolution of Head Crests in Two Domesticated Columbids Is Associated with Different Missense Mutations in EphB2. Mol Biol Evol. 2015;32 10:2657-64. doi:https://doi.org/10.1093/molbev/msv140.

Table 1 Click here to access/download;Table;Table 1.xlsx

	Blue peafowl			Green peafowl	
	This study	Liu et al. (2022)	Dhar et al. (2019)	Zhang.et al. (2022)	Dong.et.al. (2021)
Sequencing technology	PacBio (Hifi), Hi-C	Illumina NovaSeq 6000, PacBio (CLR), 10X Genomics, Chicoga	Illumina HiSeq, ONT	PacBio,Hi-C	Illumina HiSeq
Level	Chromosome	Scaffold	Scaffold	Chromosome	Scaffold
Total scaffolds	38	726	179332	115	2446
Scaffolds N50 (Mb)	95	11.4	0.19	75.5	2
Contigs N50 (Mb)	30.6	6.18	0.1	25.4	0.161
Longest scaffold length (Mb)	199.9	38.6	2.5	113.2	13.4
Total sequence length (Gb)	1.041	1.047	1.025	1.049	1.061
Total number of predicted protein-coding genes	37401	19465	23153	14935	15584

Fig 1 Click here to access/download;Figure;Fig 1.tif

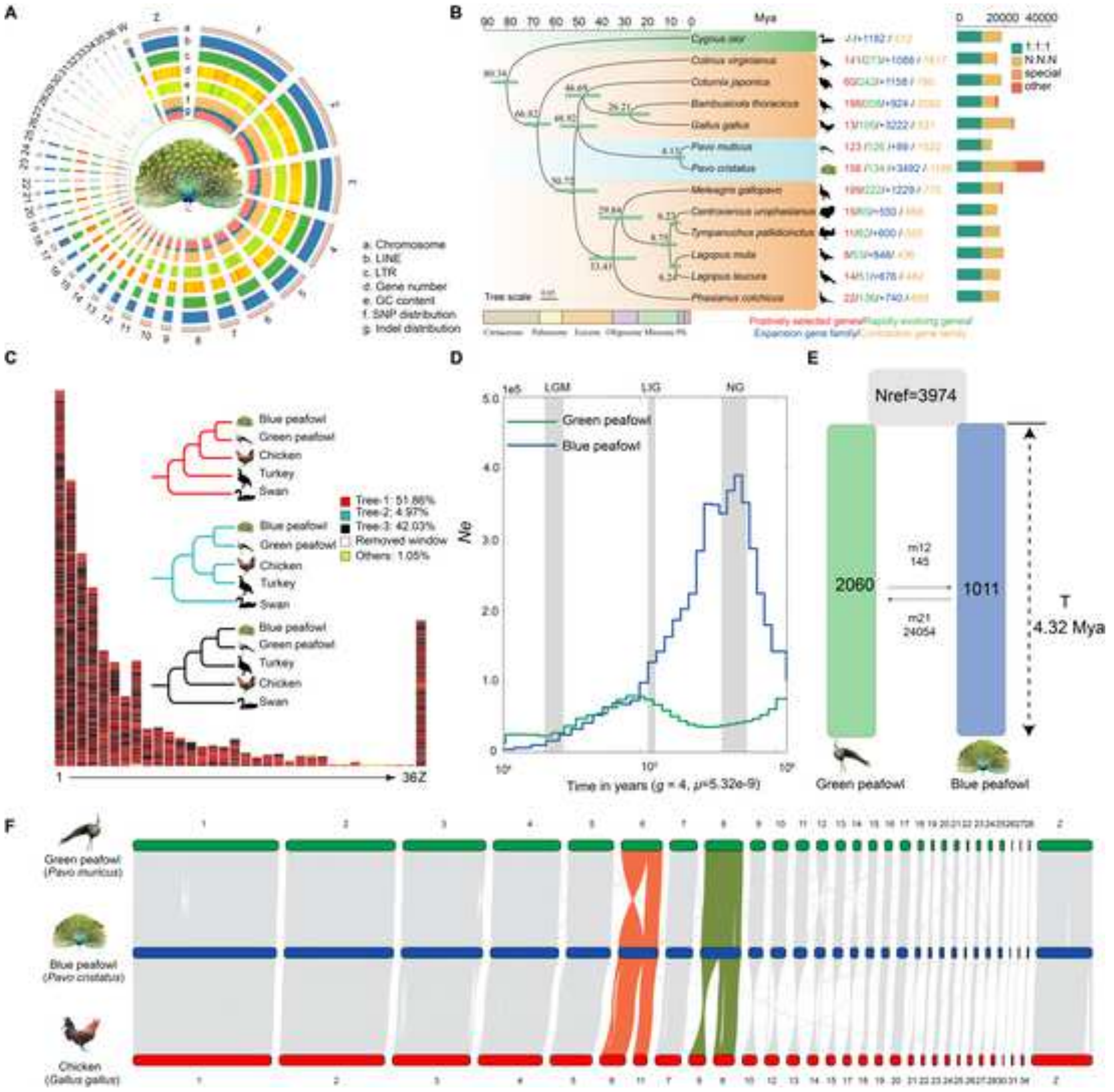

 Click here to access/download;Figure;Fig 2.tif

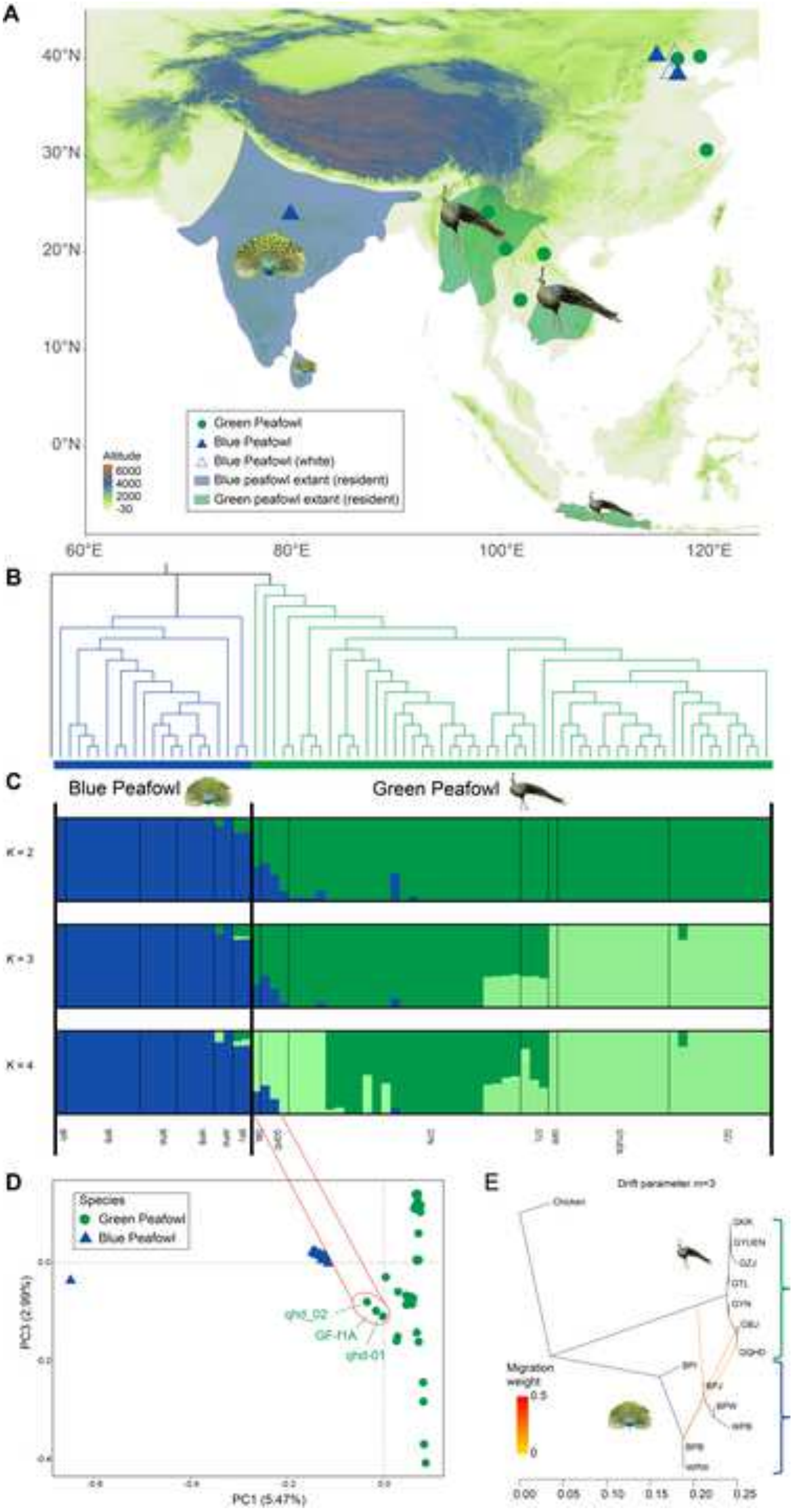

Fig 3

Click here to access/download;Figure;Fig 3.tif

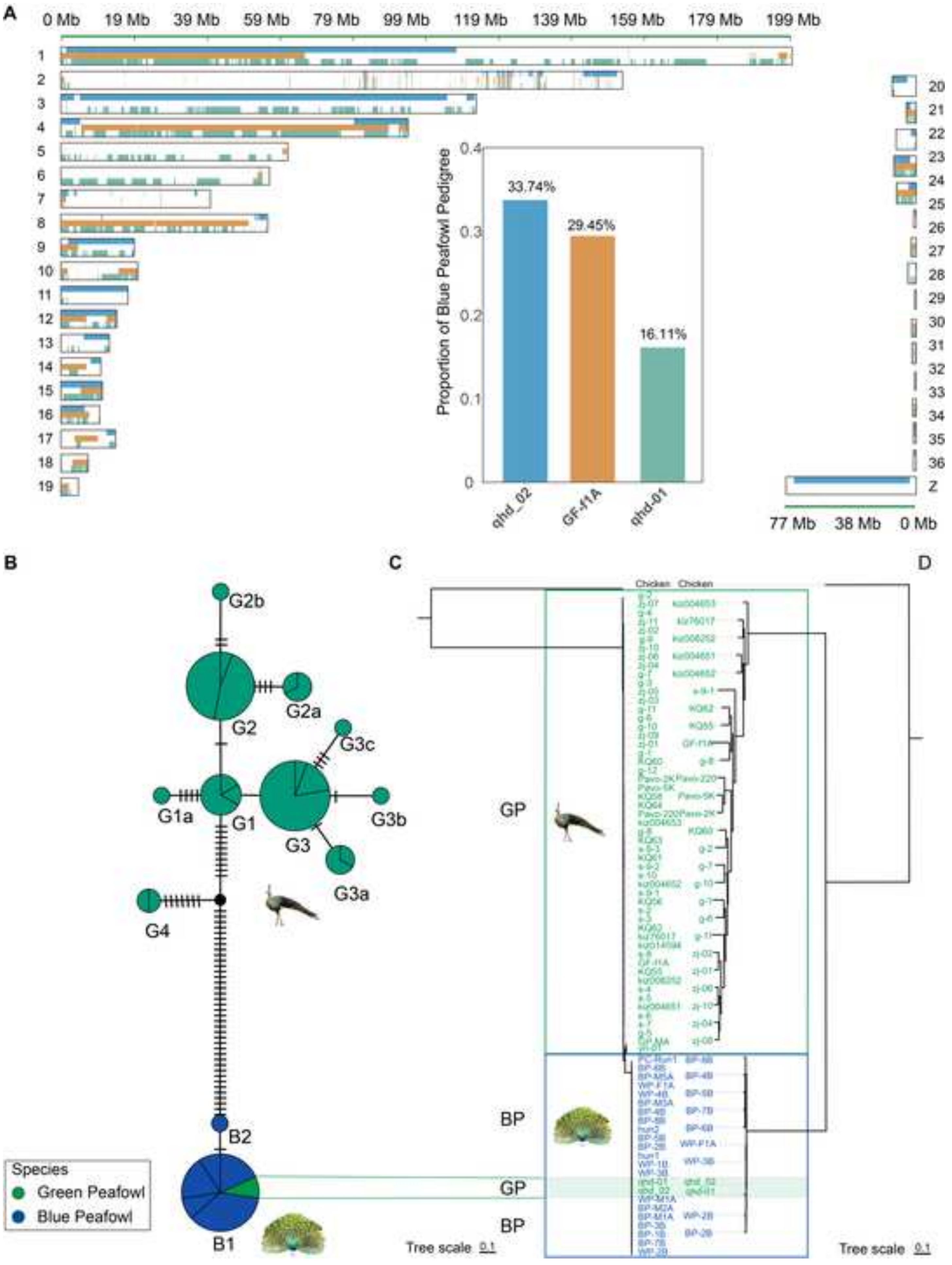

Fig 4 Click here to access/download;Figure;Fig 4.tif

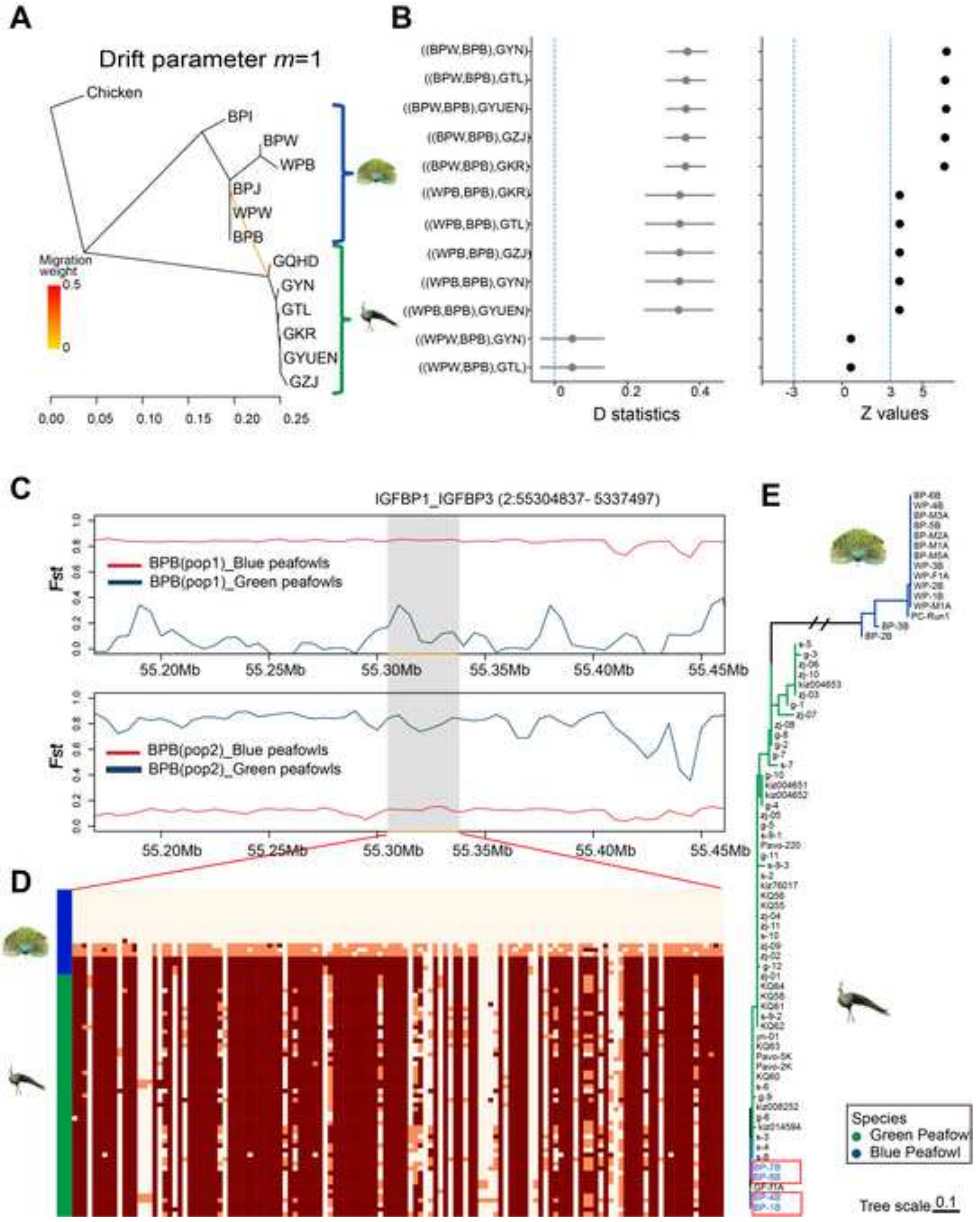

Fig 5

Click here to access/download;Figure;Fig 5.tif

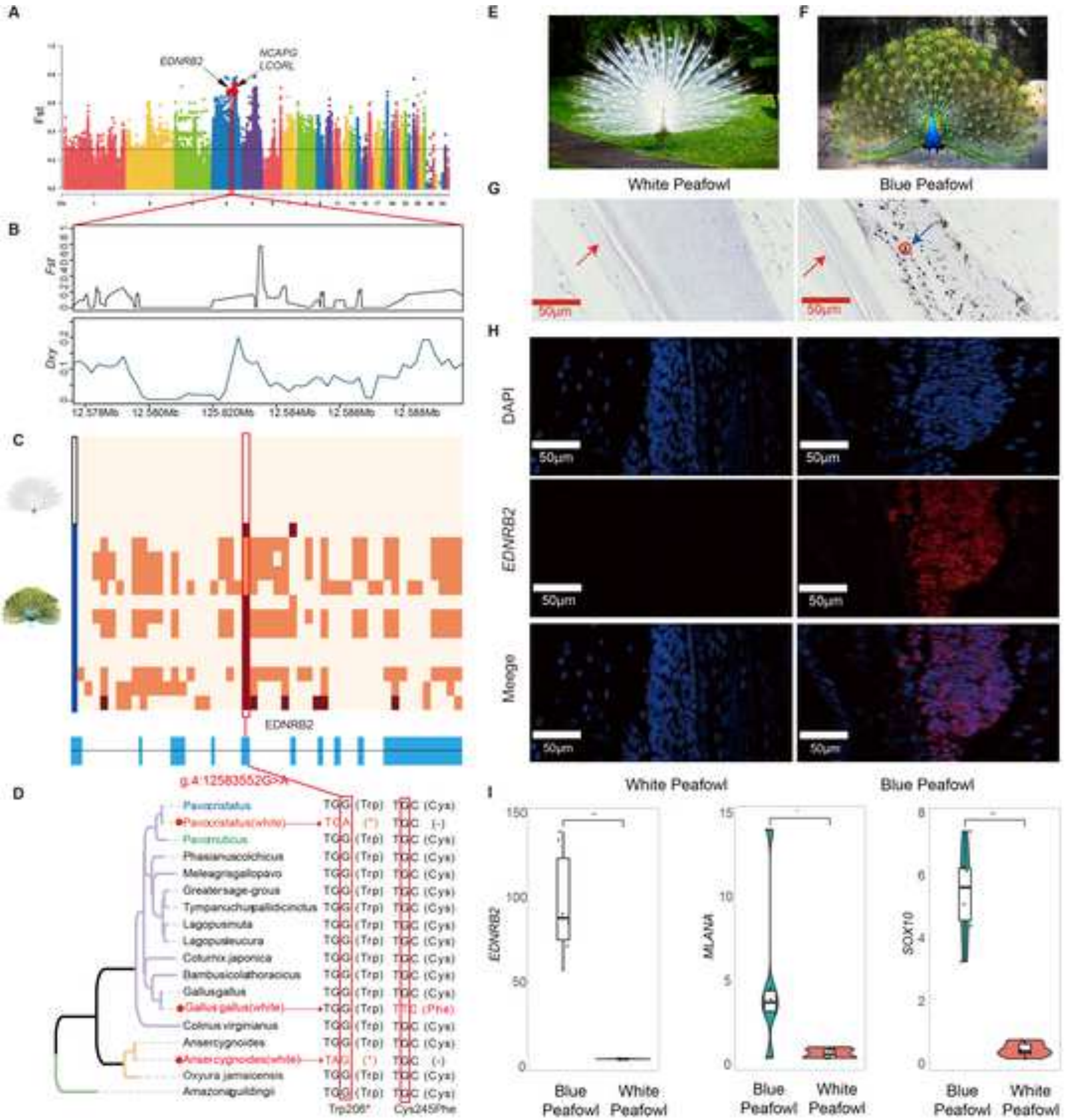

Click here to access/download
Supplementary Material
Supplementary Information1.docx

Click here to access/download
Supplementary Material
Supplementary Information2.xlsx

Coverletter
Click here to access/download;Personal Cover;cover letter.docx

Dear Editors:

Please find enclosed our manuscript entitled "**Genomic evidence for hybridization and introgression between blue peafowl and endangered green peafowl and molecular foundation of peafowl white plumage**", which we would like to submit for publication as a communication in "GigaScience". No conflict of interest exits in the submission of this manuscript, and manuscript is approved by all authors for publication. I would like to declare on behalf of my co-authors that the work described was original research that has not been published previously, and not under consideration for publication elsewhere, in whole or in part. All the authors listed have approved the manuscript that is enclosed.

In this study, we successfully assembled a high-quality chromosome-level reference genome of the blue peafowl, including the autosomes, Z and W sex chromosomes as well as a complete mitochondria DNA sequence. Data from 77 peafowl whole genomes, 76 peafowl mitochondrial genomes and 33 peahen W chromosomes genomes provide the first substantial genetic evidence for recent hybridization between green and blue peafowl. We found three hybrid green peafowls in zoo samples rather than in the wild samples, with blue peafowl genomic content of 16-34%. Maternal genetic analysis showed that two of the hybrid female green peafowls contained complete blue peafowl mitochondrial genomes and W chromosomes. Hybridization of endangered species with its relatives is extremely detrimental to conservation. Some animal protection agencies release captive green peafowls in order to maintain the wild population of green peafowls. Therefore, in order to better protect the endangered green peafowl, we suggest that purebred identification must be carried out before releasing green peafowls from zoos into the wild in order to preventing the hybrid green peafowl from contaminating the wild green peafowl. In addition, we also found that there were historical introgression events of green peafowl to blue peafowl in four Zoo blue peafowl individuals. The introgressed genomic regions contain *IGFBP1* and *IGFBP3* genes that could affect blue peafowl body size. Finally, we identified that the nonsense mutation (g.4:12583552G>A) in the EDNRB2 gene is the genetic causative mutation for white feather color of blue peafowl (also called white peafowl), which prevents melanocytes from being transported into feathers, such that melanin cannot be deposited. Our research provides both theoretical and empirical support for the conservation of the endangered green peafowl, as well as the sustainable management and breeding of the blue peafowl population.

This manuscript was submitted as a preprint on **bioRxiv.** doi: https://doi.org/10.1101/2023.12.27.573425

I hope this paper is suitable for "GigaScience".

We deeply appreciate your consideration of our manuscript, and we look forward to receiving comments from the reviewers. If you have any queries, please don't hesitate to contact me at the address below.

Thank you and best regards.

Yours sincerely,

Lujiang Qu, Ph.D.
College of Animal Science and Technology, China Agricultural University
E-mail: quluj@163.com
Telephone number: +86-13126559446

 Click here to access/download;Graphical Abstract;renamed_4f0cd.tif

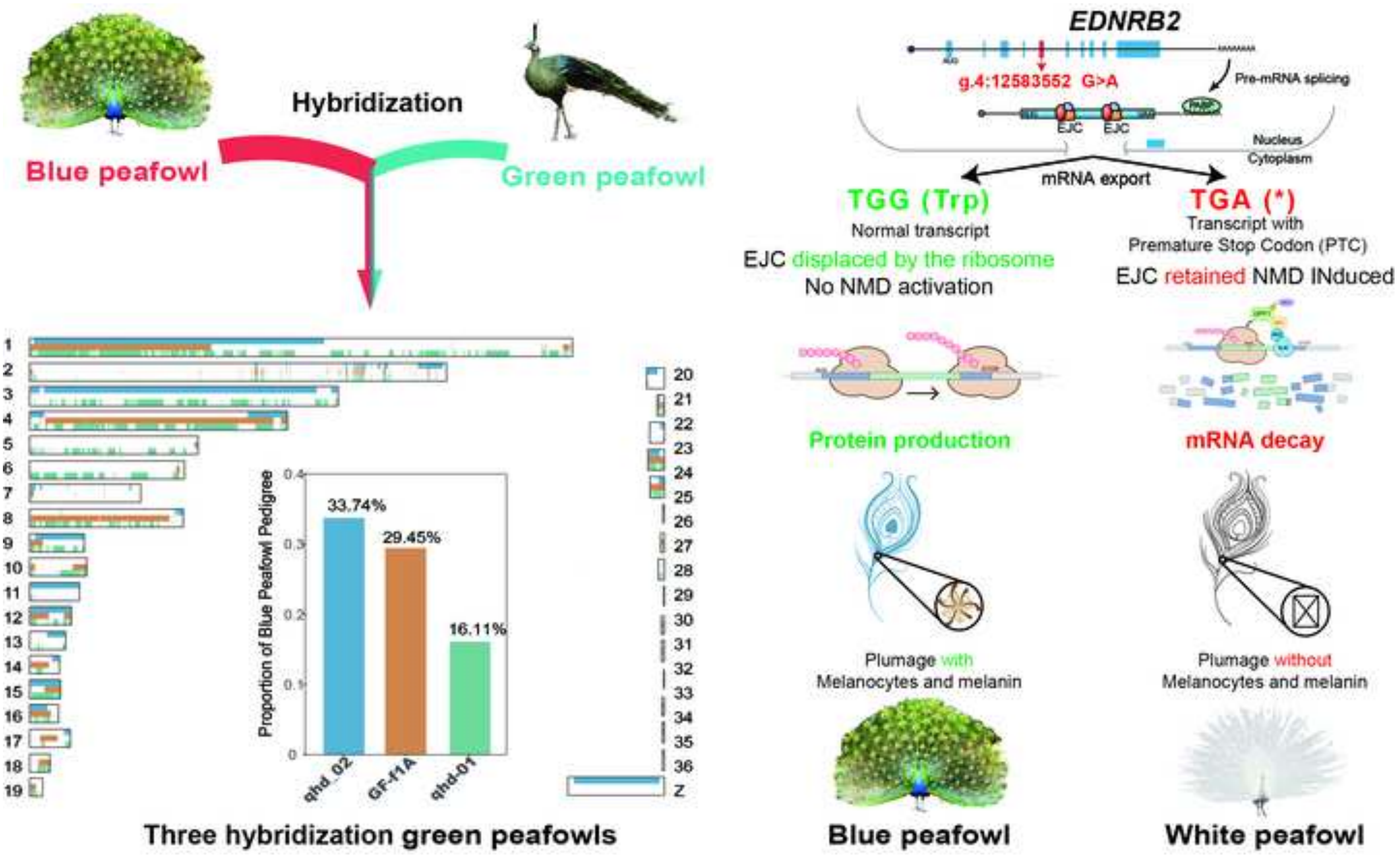

Supplement: giae124_GIGA-D-24-00290_Original_Submission [file giae124_giga-d-24-00290_original_submission.pdf]
