# Supplementary material for: Genomic evidence for hybridization and introgression between blue peafowl and endangered green peafowl and molecular foundation of leucistic plumage of blue peafowl
Source: Gigascience. 2025 Feb 19;14:giae124. doi: 10.1093/gigascience/giae124 (PMC11835448; doi:10.1093/gigascience/giae124)
Supplement: giae124_Response_to_Reviewer_Comments_Original_Submission [file giae124_response_to_reviewer_comments_original_submission.pdf]

# Author's Response To Reviewer Comments

Dear Editors and Reviewers:

Thank you for your letter and for the reviewers' comments concerning our manuscript entitled "Genomic evidence for hybridization and introgression between blue peafowl and endangered green peafowl and molecular foundation of leucism plumage in blue peafowl" (ID: GIGA-D-24-00290). Those comments are all valuable and very helpful for revising and improving our paper. We have studied the comments carefully and have made correction which we hope meet with your approval. We have marked the revised parts in yellow in the newly submitted manuscript.

Reviewer #1:
The authors had finished very systematic and comprehensive research. They obtained a high-quality chromosome-level reference genome of the blue peafowl, including the autosomes, Z and W sex chromosomes as well as a complete mitochondria DNA sequence by combined several sequencing technologies（HiFi sequencing and Hi-C sequencing）. Based on this, they further confirmed the evidence of introgression between blue peafowl and green peafowl. In addition, it is finding the nonsense mutation (g.4:12583552G>A) in the EDNRB2 gene as the causative mutation for white feather color of blue peafowl that identifies an important gap on the genetic mechanism of the white plumage in the peafowl. Overall, The results and resources obtained from this study are valuable further comparative genomic studies in birds. The analyses are also sound and comprehensive. However, before considering acceptance, there are some questions and clarifications needed from the authors to fully substantiate the findings and their implications.
Question 1 i) The "Results" section of the paper contains extensive analysis and discussion, which overlaps significantly with the "Discussion" section. It is recommended to consolidate and streamline these sections.
Answer: Thank you very much for your suggestion. We have revisited the Results and Discussion sections and removed the duplicate content.

Question 2 ii) The authors used 'white feather' peafowl throughout the manuscript. Actually there are scientific terms about these color abnormality, for instance, leucism or albino plumage. Please define whether your samples from leucitic or albino populations. Also please change the term 'white feather' throughout the manuscript.
Answer: Thank you for pointing out the problem with scientific terminology in this article. The white peafowl we used in this are leucistic plumage forms of the blue peafowl. We have revised the description of white peafowl in the article and changed 'white feather' to 'leucistic plumage'.

Question 3 iii) The authors used three types of data (one-to-one orthologs datasets, four-fold degenerate sites datasets and mitochondrial sequence datasets) to study the genetic relationships between peacocks, chickens, and turkeys, and proved that the genetic distance between peacocks and chickens is closer (See Line 859-862). However, from the results section, in Figure 1C, the pattern of tree3 shows that the genetic distance between peacocks and turkeys appears to be closer, suggesting a certain contradiction between the results and the discussion sections.
Answer: Thank you very much for your question about the phylogenetic relationships of peafowls, chickens and turkeys. In this study, we used three types of data to find that the genetic distance between peafowls and chickens is closest. However, previous studies have shown that the genetic distance between peafowls and turkeys is closer [1]. For these two completely different results, we speculate that incomplete lineage sorting (ILS) and introgression caused by the rapid process of species formation are two important reasons for their appearance. Therefore, we used a 100KB non-overlapping window to construct a phylogenetic tree for the alignment data of peafowls, chickens and turkeys in the whole genome, as shown in Figure 1C. Among them, the tree that supports the closer genetic distance between peafowls and chickens (Tree-1: 51.86%) accounts for more than half of the whole genome, which is why we used three types of data to obtain the closer genetic distance between peafowls and chickens. However, we also found that the proportion of trees that support the closer genetic distance between peafowls and turkeys (Tree-3: 42.03%) in the whole genome is very high. This shows that the results of the closer genetic distance

between peafowls and turkeys obtained in previous studies are due to the influence of ILS on the construction of the real species tree. The impact of ILS on the construction of a true species tree has been found in studies of primates and other organisms [2]. We have revised the content of this section.

[1] Liu S, Chen H, Ouyang J, Huang M, Zhang H, Zheng S, et al. A high-quality assembly reveals genomic characteristics, phylogenetic status, and causal genes for leucism plumage of Indian peafowl. Gigascience. 2022;11

[2] Rivas-González I, Rousselle M, Li F, et al. Pervasive incomplete lineage sorting illuminates speciation and selection in primates[J]. Science, 2023, 380(6648): eabn4409.

Question 4 iv) Why were individuals with the "pied" phenotype not selected as controls for the corresponding transcriptomic study to validate the molecular mechanisms of feather formation in blue peacocks using RNA-Seq results?

Answer: Thank you for providing the research idea of using the transcriptome data of peafowl to verify the formation of plumage color in white peafowl. We collected a sample of peafowl, and the genotype of the sample at the EDNRB2 (g.4:12583552G>A) locus was A/G. The white plumage follicle tissue and blue plumage follicle tissue of the same pied peafowl were used for transcriptome sequencing. We found that the EDNRB2 and MLANA genes related to melanin formation in the white plumage follicle tissue were not expressed, while the EDNRB2 and MLANA genes in the blue plumage follicle tissue were expressed normally. The results are presented in Fig.S25.

Question 5 v) The statement in the sentence "Compared with the peafowl, the ROH length of all peafowl populations is short and the total is small (see Line 624-625)" seems to be incorrect.

Answer: Thank you for your careful review of this sentence. We have rewritten it.

Question 6 vi) The entire paper still needs further improvement in terms of writing norms and grammar. （eg. Line 642, "as an outgroup", Line 647 "The mitochondrial phylogenetic" etc ）

Answer: Thank you very much for your valuable comments. We reviewed and seriously revised the manuscript. In addition to the two grammatical issues you mentioned, we also corrected other writing errors.

Reviewer #2:

I read with interest the manuscript " Genomic evidence for hybridization and introgression between blue peafowl and endangered green peafowl and molecular Foundation of peafowl white plumage" by Lujiang et al. . This is a well-drafted, well-executed study that investigated the effect of introgression in shaping the genomic diversity landscape of peafowl. I am glad the authors undertook this much-needed study which is so critical from an evolutionary point of view. I have few queries and clarifications needed :

Question 1. Fig S21 : Manhattan Plot : What is the loci on Chr 4 & Chr 6 that showed above threshold? What are the consequences of IL12b and IL25 ?

Answer: Thank you very much for your question. There are 60 genes above the threshold line of Chr 4 & Chr 6, which are shown in Table S15. The enrichment analysis results of these genes and all introgressed genes are also shown in Figure S23. According to the question 3 you mentioned later, we also discussed the functions of these genes. IL12b and IL25 belong to the interleukin gene family. They are important cytokines in the immune process and regulate the immune response of peafowls. We believe that the infiltration of these genes into blue peafowls is the result of adaptive introgression and plays an important role in improving the survival ability of blue peafowls.

Question 2. Page 50, Line : 929 : " The genes (IGF2BP3, TGBR1, ISPD, MEOX2, GLI3 and MC4R) related to body size in blue peafowl were also found to have introgression areas from green peafowl" What is the evidence for this ? Were these genes absent before the introgression events in blue peafowl? What are the modifications of IGFBP after introgression? Is it under positive selection? If yes why

Answer: Thank you for asking this question. The introgression of these body size-related genes is included in Supplementary Table S14 (D statistics of introgression regions (BPW, BPB, GYN, chicken)). In addition, we have added Figure S22 (Phylogenetic tree of 77 peafowl individuals constructed in IGF2BP3, TGBR1,

ISPD, MEOX2, GLI3 and MC4R gene regions using ML method. ), which shows that blue peafowl individuals cluster with green peafowl in these gene regions. Introgression is the incorporation (usually via hybridization and backcrossing) of novel genes or alleles from one taxon into the gene pool of a second, distinct taxon. Before the introgression event, these genes were present in the blue peafowl. Because of the introgression, the gene frequencies of the blue peafowl individuals that were introgressed are closer to those of the green peafowl than to those of other blue peafowls. The IGFBP1 and IGFBP3 genes did not experience positive selection in blue peafowls (S9 Table). The gene frequencies of the regions where these two genes are located in some blue peafowl individuals are closer to those of green peafowls (Fig. 4 C, D and E).

Question 3. There is not much discussion on Fig S 22 ( Suppl) on the KEGG Pathway hits. What is the significance of ribosome biogenesis? Protein processing in ER, etc
Answer: Thank you very much for your valuable suggestions. We have added a discussion of GO and KEGG in the Results section. We analyzed the relationship between the GO terms and KEGG pathways shown in the figure and the adaptive introgression of blue peafowls. This section has been added to L714-L25, and related content was also added to the Discussion section.

Question 4. The white peafowls were homozygous for the mutant (A/A), resulting in the loss of EDNRB2 transcript. What is the reason for this mutant gene's fixation in white plumage birds?
Answer: Thank you very much for your question. The reasons why the mutation site is fixed by the population mainly include artificial selection and natural selection. If the beneficial allele can give an individual a great survival advantage, it will be fixed quickly under natural conditions. However, although the nonsense mutation of the EDNRB2 gene will not cause fatal damage to the peafowl individual, the white plumage phenotype caused by this mutant does not have a survival advantage for peafowls that mainly live in the jungle on land rather than flying in the sky. Therefore, we are more inclined to believe that the fixation of the nonsense mutation of the EDNRB2 gene in white peafowls is the result of artificial selection, mainly to meet human needs for ornamental purposes.
Question 5. The images, almost all of them, appear very hazy and blurry. It may be an issue with my computer. Please recheck
Answer: We attach great importance to the question you raised. We have further improved the resolution of the pictures and uploaded 300dpi high-definition pictures in the system. If you are still confused about the clarity of the pictures, please feel free to contact me.

Question 6. Please elaborate on the significance of IL6 and other immune-related genes in the discussion.
Answer: Thank you very much for your valuable suggestions. We have rewritten the content about the effects of immune gene introgression on blue peafowl fitness. This part has been added to L945-L951, and the corresponding references have been added.

Finally, I would like to thank the editor and two reviewers again for their valuable comments. If you have any queries, please don’t hesitate to contact me at the address below.

Thank you and best regards.
Yours sincerely,
Lujiang Qu, Ph.D.
College of Animal Science and Technology, China Agricultural University
E-mail: quluj@163.com
Telephone number: +86-13126559446
