# Supplementary material for: Genomic evidence for hybridization and introgression between blue peafowl and endangered green peafowl and molecular foundation of leucistic plumage of blue peafowl
Source: Gigascience. 2025 Feb 19;14:giae124. doi: 10.1093/gigascience/giae124 (PMC11835448; doi:10.1093/gigascience/giae124)
Supplement: giae124_Reviewer_1_Report_Original_Submission — huirong Mao -- 9/3/2024 [file giae124_reviewer_1_report_original_submission.pdf]

Reviewer Report

Title: Genomic evidence for hybridization and introgression between blue peafowl and endangered green peafowl and molecular foundation of leucistic plumage of blue peafowl

Version: Original Submission **Date:** 9/3/2024

Reviewer name: huirong Mao

Reviewer Comments to Author:

The authors had finished very systematic and comprehensive research. They obtained a high-quality chromosome-level reference genome of the blue peafowl, including the autosomes, Z and W sex chromosomes as well as a complete mitochondria DNA sequence by combined several sequencing technologiesï¼ˆHiFi sequencing and Hi-C sequencingï¼‰. Based on this, they further confirmed the evidence of introgression between blue peafowl and green peafowl. In addition, it is finding the nonsense mutation (g.4:12583552G>A) in the EDNRB2 gene as the causative mutation for white feather color of blue peafowl that identifies an important gap on the genetic mechanism of the white plumage in the peafowl. Overall, The results and resources obtained from this study are valuable further comparative genomic studies in birds. The analyses are also sound and comprehensive. However, before considering acceptance, there are some questions and clarifications needed from the authors to fully substantiate the findings and their implications.
i) The "Results" section of the paper contains extensive analysis and discussion, which overlaps significantly with the "Discussion" section. It is recommended to consolidate and streamline these sections.
ii) The authors used 'white feather' peafowl throughout the manuscript. Actually there are scientific terms about these color abnormality, for instance, leucism or albino plumage. Please define whether your samples from leucitic or albino populations. Also please change the term 'white feather' throughout the manuscript.
iii) The authors used three types of data (one-to-one orthologs datasets, four-fold degenerate sites datasets and mitochondrial sequence datasets) to study the genetic relationships between peacocks, chickens, and turkeys, and proved that the genetic distance between peacocks and chickens is closer (See Line 859-862). However, from the results section, in Figure 1C, the pattern of tree3 shows that the genetic distance between peacocks and turkeys appears to be closer, suggesting a certain contradiction between the results and the discussion sections.
iv) Why were individuals with the "pied" phenotype not selected as controls for the corresponding transcriptomic study to validate the molecular mechanisms of feather formation in blue peacocks using RNA-Seq results?
v) The statement in the sentence "Compared with the peafowl, the ROH length of all peafowl populations is short and the total is small (see Line 624-625)" seems to be incorrect.
vi) The entire paper still needs further improvement in terms of writing norms and grammar. ï¼ˆeg. Line 642, "as an outgroup", Line 647 "The mitochondrial phylogenetic" etc ï¼‰

Methods

Are the methods appropriate to the aims of the study, are they well described, and are necessary controls included? Choose an item.

Conclusions

Are the conclusions adequately supported by the data shown? Choose an item.

Reporting Standards

Does the manuscript adhere to the journal's guidelines on minimum standards of reporting? Choose an item.

Choose an item.

Statistics

Are you able to assess all statistics in the manuscript, including the appropriateness of statistical tests used? Choose an item.

Quality of Written English

Please indicate the quality of language in the manuscript: Choose an item.

Declaration of Competing Interests

Please complete a declaration of competing interests, considering the following questions:

- Have you in the past five years received reimbursements, fees, funding, or salary from an organisation that may in any way gain or lose financially from the publication of this manuscript, either now or in the future?
- Do you hold any stocks or shares in an organisation that may in any way gain or lose financially from the publication of this manuscript, either now or in the future?
- Do you hold or are you currently applying for any patents relating to the content of the manuscript?
- Have you received reimbursements, fees, funding, or salary from an organization that holds or has applied for patents relating to the content of the manuscript?
- Do you have any other financial competing interests?
- Do you have any non-financial competing interests in relation to this paper?

If you can answer no to all of the above, write 'I declare that I have no competing interests' below. If your reply is yes to any, please give details below.

I declare that I have no competing interests.

I agree to the open peer review policy of the journal. I understand that my name will be included on my report to the authors and, if the manuscript is accepted for publication, my named report including any attachments I upload will be posted on the website along with the authors' responses. I agree for my report to be made available under an Open Access Creative Commons CC-BY license (http://creativecommons.org/licenses/by/4.0/). I understand that any comments which I do not wish to be included in my named report can be included as confidential comments to the editors, which will not be published.

Choose an item.

To further support our reviewers, we have joined with Publons, where you can gain additional credit to further highlight your hard work (see: https://publons.com/journal/530/gigascience). On publication of this paper, your review will be automatically added to Publons, you can then choose whether or not to claim your Publons credit. I understand this statement.

Yes Choose an item.
