# Supplementary material for: Genomic evidence for hybridization and introgression between blue peafowl and endangered green peafowl and molecular foundation of leucistic plumage of blue peafowl
Source: Gigascience. 2025 Feb 19;14:giae124. doi: 10.1093/gigascience/giae124 (PMC11835448; doi:10.1093/gigascience/giae124)
Supplement: giae124_Reviewer_2_Report_Original_Submission — Subhradip Karmakar, PhD -- 9/16/2024 [file giae124_reviewer_2_report_original_submission.pdf]

Reviewer Report

Title: Genomic evidence for hybridization and introgression between blue peafowl and endangered green peafowl and molecular foundation of leucistic plumage of blue peafowl

Version: Original Submission **Date:** 9/16/2024

Reviewer name: Subhradip Karmakar, PhD

Reviewer Comments to Author:

I read with interest the manuscript " Genomic evidence for hybridization and introgression between blue peafowl and endangered green peafowl and molecular Foundation of peafowl white plumage" by Lujiang et al. . This is a well-drafted, well-executed study that investigated the effect of introgression in shaping the genomic diversity landscape of peafowl. I am glad the authors undertook this much-needed study which is so critical from an evolutionary point of view. I have few queries and clarifications needed :

1. Fig S21 : Manhattan Plot : What is the loci on Chr 4 & Chr 6 that showed above threshold? What are the consequences of IL12b and IL25 ?
2. Page 50, Line : 929 : " The genes (IGF2BP3, TGBR1, ISPD, MEOX2, GLI3 and MC4R) related to body size in blue peafowl were also found to have introgression areas from green peafowl" What is the evidence for this ? Were these genes absent before the introgression events in blue peafowl? What are the modifications of IGFBP after introgression? Is it under positive selection? If yes why
3. There is not much discussion on Fig S 22 (Suppl) on the KEGG Pathway hits. What is the significance of ribosome biogenesis? Protein processing in ER, etc
4. The white peafowls were homozygous for the mutant (A/A), resulting in the loss of EDNRB2 transcript. What is the reason for this mutant gene's fixation in white plumage birds?
5. The images, almost all of them, appear very hazy and blurry. It may be an issue with my computer. Please recheck
6. Please elaborate on the significance of IL6 and other immune-related genes in the discussion.

Methods

Are the methods appropriate to the aims of the study, are they well described, and are necessary controls included? Choose an item.

Conclusions

Are the conclusions adequately supported by the data shown? Choose an item.

Reporting Standards

Does the manuscript adhere to the journal's guidelines on [minimum standards of reporting?] Choose an item.

Choose an item.

Statistics

Are you able to assess all statistics in the manuscript, including the appropriateness of statistical tests used? Choose an item.

Quality of Written English

Please indicate the quality of language in the manuscript: Choose an item.

Declaration of Competing Interests

Please complete a declaration of competing interests, considering the following questions:

- Have you in the past five years received reimbursements, fees, funding, or salary from an organisation that may in any way gain or lose financially from the publication of this manuscript, either now or in the future?
- Do you hold any stocks or shares in an organisation that may in any way gain or lose financially from the publication of this manuscript, either now or in the future?
- Do you hold or are you currently applying for any patents relating to the content of the manuscript?
- Have you received reimbursements, fees, funding, or salary from an organization that holds or has applied for patents relating to the content of the manuscript?
- Do you have any other financial competing interests?
- Do you have any non-financial competing interests in relation to this paper?

If you can answer no to all of the above, write 'I declare that I have no competing interests' below. If your reply is yes to any, please give details below.

NO

I agree to the open peer review policy of the journal. I understand that my name will be included on my report to the authors and, if the manuscript is accepted for publication, my named report including any attachments I upload will be posted on the website along with the authors' responses. I agree for my report to be made available under an Open Access Creative Commons CC-BY license (http://creativecommons.org/licenses/by/4.0/). I understand that any comments which I do not wish to be included in my named report can be included as confidential comments to the editors, which will not be published.

Choose an item.

To further support our reviewers, we have joined with Publons, where you can gain additional credit to further highlight your hard work (see: https://publons.com/journal/530/gigascience). On publication of this paper, your review will be automatically added to Publons, you can then choose whether or not to claim your Publons credit. I understand this statement.

Yes Choose an item.
